# Age-related dysregulation of homeostatic control in neuronal microcircuits

**DOI:** 10.1038/s41593-023-01451-z

**Published:** 2023-11-02

**Authors:** Carola I. Radulescu, Nazanin Doostdar, Nawal Zabouri, Leire Melgosa-Ecenarro, Xingjian Wang, Sadra Sadeh, Pavlina Pavlidi, Joe Airey, Maksym Kopanitsa, Claudia Clopath, Samuel J. Barnes

**Affiliations:** 1 UK Dementia Research Institute, Department of Brain Sciences, Imperial College London, Hammersmith Hospital Campus, London, UK; 2 Department of Biomedical Engineering, Imperial College London, South Kensington Campus, London, UK; 3 The Francis Crick Institute, London, UK; 4 Present Address: Department of Brain Sciences, Imperial College London, Hammersmith Hospital Campus, London, UK; 5 Present Address: Department of Pharmacology, Medical School, National and Kapodistrian University of Athens, Athens, Greece

**Keywords:** Synaptic plasticity, Cognitive ageing

## Abstract

Neuronal homeostasis prevents hyperactivity and hypoactivity. Age-related hyperactivity suggests homeostasis may be dysregulated in later life. However, plasticity mechanisms preventing age-related hyperactivity and their efficacy in later life are unclear. We identify the adult cortical plasticity response to elevated activity driven by sensory overstimulation, then test how plasticity changes with age. We use in vivo two-photon imaging of calcium-mediated cellular/synaptic activity, electrophysiology and c-Fos-activity tagging to show control of neuronal activity is dysregulated in the visual cortex in late adulthood. Specifically, in young adult cortex, mGluR5-dependent population-wide excitatory synaptic weakening and inhibitory synaptogenesis reduce cortical activity following overstimulation. In later life, these mechanisms are downregulated, so that overstimulation results in synaptic strengthening and elevated activity. We also find overstimulation disrupts cognition in older but not younger animals. We propose that specific plasticity mechanisms fail in later life dysregulating neuronal microcircuit homeostasis and that the age-related response to overstimulation can impact cognitive performance.

## Abstract (web summary)

Radulescu et al. show that homeostatic mechanisms that reduce cortical activity following overstimulation are dysregulated later in life, such that overstimulation results in synaptic strengthening, elevated activity and cognitive impairment.

## Main

Homeostatic regulation of neuronal activity promotes stable neural circuit function by preventing extreme spiking^1^. Regulation of neuronal activity occurs through homeostatic plasticity mechanisms^2,3^ operating at the network, cell and synapse^1–4^. However, the efficacy of such mechanisms in later life is unclear^5–7^. Regulation of activity in the aged brain is important, as neural hyperactivity may increase susceptibility to neurodegeneration^8,9^ and impair cognition^10^. Age-related hyperactivity occurs in multiple species and brain regions, suggesting that regulatory plasticity mechanisms preventing hyperactivity in young adult cortex may fail in later life^8,10–14^. Understanding such early age-related changes may facilitate targeting of vulnerable processes.

Current understanding of plasticity mechanisms preventing neural hyperactivity in the adult cortex is limited. The majority of in vivo work investigating mature regulatory plasticity processes has focused on adaptation to reduced activity^1–3,15^. However, work in neuronal culture finds plasticity processes can modify the synaptic excitatory-to-inhibitory (E:I) ratio to compensate for prolonged chemically induced hyperactivity^16,17^. The E:I ratio can be modified through processes such as synaptic downscaling^17–20^. This cell-autonomous mechanism broadly reduces synaptic strength and could prevent saturation of synaptic weights and maintain neuronal firing within a stable dynamic range^21^. These population-level changes in synaptic strength can be described by a single multiplicative or additive scaling factor^17,22^. Population-level weakening of excitatory synapses can involve group I metabotropic glutamate receptors (mGluR1 and mGluR5) and the mGluR1/mGluR5–Homer1 signaling pathway in juvenile and adult cortex^23–25^. However, the expression/efficacy of such regulatory plasticity during aging remains unclear.

Plastic changes in excitatory synaptic strength may work with other regulatory mechanisms, such as changes in synaptic inhibition or modifications of intrinsic excitability, to modulate activity levels following elevated activity^20^. Whether different processes are more susceptible to aging remains unclear^5^, as do the implications for network function and cognition^26^. Age-related downregulation of one plasticity mechanism may be compensated by another^27^. For example, some animal models express an age-related shift from homeostatic to more Hebbian-like plasticity^28^. Thus, rather than a general decline, plasticity may be differentially regulated across mechanisms, pathways, cell types and/or brain regions during aging^5^.

We found age-related changes in the regulation of neuronal activity between young (3 months) and late (12 months) adulthood in mouse primary visual cortex (V1). This age range tested plasticity differences before widespread neuronal hyperactivity and pronounced sensorimotor deficits^5^. We used visual overstimulation to drive elevated cortical activity and combined two-photon (2-P) calcium imaging with c-Fos-activity tagging and electrophysiology, to identify plasticity mechanisms in young adult cortex and investigate age-related changes. We found age-related dysregulation of population-level multiplicative excitatory synaptic weakening and formation of inhibitory inputs onto excitatory neurons. In young adult cortex, these mechanisms reduced synaptic E:I ratios and dampened cortical activity following overstimulation. Neuronal and subcellular calcium imaging found overstimulation reduced dendritic spine activity and strengthened functional associations between excitatory and inhibitory neuronal assemblies in young adult cortex. Older animals exhibited failures in identified plasticity mechanisms, showing greater synaptic strengthening after overstimulation, which increased the E:I ratio and strengthened associations between excitatory neurons. Overstimulation also disrupted subsequent cognitive performance in older but not younger animals. A positive allosteric modulator (PAM) of mGluR5 signaling prevented the negative impact of overstimulation on cognition in older animals, while blockade of mGluR5-dependent processes promoted the negative impact of overstimulation on cognition in young adult animals. Our results find specific synaptic plasticity mechanisms fail in later life leading to dysregulation of neuronal activity homeostasis. We propose that disrupted homeostatic plasticity has consequences for network plasticity and that the age-related response to sensory overstimulation can impact cognitive performance.

## Results

### Neuronal overstimulation response in young adult cortex

Age-related hyperactivity may be due to declining regulatory plasticity mechanisms^1,8,10–14,29,30^. To capture emerging changes and age-related plasticity differences, we focused on ages preceding the classical definition of rodent old age, widespread neuronal hyperactivity and pronounced sensorimotor deficits^5^. We therefore tested for plasticity mechanisms triggered by elevated cortical activity in young adult mice (3 months old) and the efficacy of plasticity in later life (8–12 months old).

**Fig. 1:**
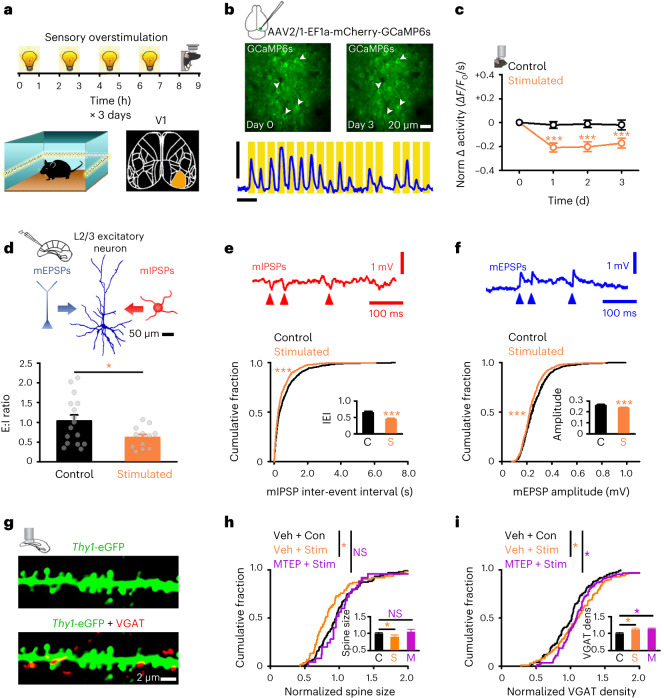
Cortical adaptation to sensory overstimulation in young adults. **a**, Schematic of overstimulation timeline and imaged region (V1). **b**, Example 2-P calcium imaging in V1 at day 0 (before overstimulation, at baseline) and day 3 (after 3 d of overstimulation). Arrows show neurons active in both days. *ΔF/F*_0_ trace from a representative V1 L2/3 neuron in response to light stimulation (yellow bars). Scale bars, 100% *ΔF/F*_0_, 10 s and 20 µm. **c**, Normalized change (versus day 0) in calcium-mediated neuronal activity (*ΔF/F*_0_ integral per second) from neurons in overstimulated (orange) and control (black) anesthetized mice (two-way analysis of variance (ANOVA), control (Con) versus stimulated (Stim) day 1, *P* < 0.001; day 2, *P* < 0.001; day 3, *P* < 0.001). **d**–**f**, mEPSP/mIPSP recordings from V1 L2/3 pyramidal neurons measuring E:I synaptic ratios (Welch’s *t*-test, Con versus Stim, *P* = 0.023) (**d**), mIPSP inter-event interval (Mann-Whitney Rank Sum Test (MWRST) Con versus Stim, *P* < 0.001) (**e**) and mEPSP amplitude (mV) (MWRST, Con versus Stim, *P* < 0.001) (**f**) in overstimulated (orange) and control (black) mice. Top, filled neuron (**d**) and traces showing mIPSPs (red arrowheads; **e**) and mEPSPs (blue arrowheads; **f**). Scale bars, 50 µm (**d**), 1 mV and 100 ms (**e** and **f**). **g**, *Thy1*-eGFP-positive dendrite from V1 L2/3 excitatory neuron (top, green) and VGAT immunofluorescence staining (bottom, red). Scale bar, 2 µm. **h**,**i**, Normalized spine size (Kruskal–Wallis one-way ANOVA, vehicle (Veh) + Stim versus Veh + Con, *P* = 0.018; Veh + Con versus MTEP + Stim, *P* = 0.911) (**h**) and VGAT puncta density (Kruskal–Wallis one-way ANOVA, Veh + Stim versus Veh + Con, *P* = 0.036; and Veh + Con versus MTEP + Stim, *P* = 0.043) (**i**) calculated per branch at V1 L2/3 neurons of *Thy1*-eGFP mice that received vehicle and no stimulation (black, ‘C’), vehicle and overstimulation (orange, ‘S’) or MTEP and overstimulation (purple, ‘M’). Insets show average spine size (**h**) and VGAT density (**i**) per dendrite normalized to vehicle and no stimulation (black, ‘C’) for all conditions. Data were obtained from 7 animals for **c**, 19 animals for **d**–**f** and 9 animals for panels **g**–**i**. *P < 0.05, ***P < 0.001; NS, not significant. Two-sided tests were used throughout. Detailed statistics are reported in Supplementary Table 1. Data are presented as the mean ± s.e.m. with individual data points (gray dots) or as a cumulative distribution. Source data

We developed a visual overstimulation paradigm to elevate activity of layer 2/3 (L2/3) excitatory neurons in V1, a locus of adult plasticity^2,3^ (Fig. 1a,b and Supplementary Fig. 1a,b). Overstimulation involved repeated 1-h sessions (S) with a 40-Hz light flicker in the home cage (four times per day, 1 h on/1 h off) across the light cycle (Methods and Fig.1a). We found that two 1-h sessions of overstimulation acutely elevated cortical c-Fos activity (Methods and Supplementary Fig. 1a,b). We then performed longitudinal in vivo 2-P recordings of calcium-mediated activity at L2/3 excitatory neurons expressing GCaMP6s in anesthetized mice (Methods and Fig. 1a,b). We imaged before (day 0) and after each overstimulation day (days 1–3) (Methods and Fig. 1a–c). Recordings were made during light-flicker (matching overstimulation stimulus) and resting-state activity (Methods and Fig.1a,b). Neuronal activity was estimated using the area under the curve (AUC) of the calcium fluoresence response (*ΔF/F*_0_) per second and normalized to baseline (day 0; Methods). We found a sustained (days 1–3) reduction in cortical activity after overstimulation in young adult (3 months old) cortex compared to control animals (Fig. 1c).

We next measured more fine-scale activity changes in L2/3 neurons by recording after each hour-long overstimulation session in anesthetized mice. We again quantified the AUC of *ΔF/F*_0_ calcium signal per second (Supplementary Fig. 1c) and, consistent with c-Fos measures (Supplementary Fig. 1a,b), found an initial elevation in total activity across S1–3 of day 1 (Supplementary Figs. 1c and 2f). The acute elevation was followed by a reduction to values less than baseline by S4 of day 1 (Supplementary Figs. 1c and 2f). More prolonged overstimulation (days 2–3) resulted in total activity levels being less than baseline after each day of overstimulation (Supplementary Figs. 1c and 2f). The overstimulation response was driven by changes in *ΔF/F*_0_ amplitude, frequency or both (Supplementary Fig. 2f,h,j,l,n and Supplementary Table 8) and was evident in both visually evoked and spontaneous activity (Supplementary Fig. 2f,h,j,l,n and Supplementary Table 8).

Experience-dependent changes in neuronal activity can occur independently of *N*-methyl-d-aspartate receptors (NMDARs)^22^. To test if the overstimulation response was NMDAR dependent, we used 3-(2-carboxypiperazin-4-yl)propyl-1-phosphonic acid (CPP), an NMDAR antagonist blocking some forms of Hebbian plasticity for up to 24 h without affecting visual function^31–33^. We administered CPP 24 h before and immediately before overstimulation, and then we performed imaging (Methods and Supplementary Fig. 1d,e). The acute increase (S3 of day 1) and subsequent decrease in activity were NMDAR independent (Supplementary Fig. 1e). Our results suggest overstimulation drives an NMDAR-independent adaptation of neuronal activity in young adult V1.

### Synaptic plasticity after overstimulation in young adult cortex

Changes in neuronal activity may occur through shifts in the synaptic E:I ratio^2^. We measured the E:I ratio with electrophysiological recordings of miniature excitatory postsynaptic potentials (mEPSPs) and miniature inhibitory postsynaptic potentials (mIPSPs) from L2/3 excitatory neurons in brain slices containing V1 in overstimulated and control mice (Fig.1d–f and Supplementary Fig. 1f–i). Mice received 3 d of overstimulation, with tissue collected 24 h later so that plasticity, rather than the immediate impact of overstimulation, could be assessed (Supplementary Fig. 1f). The E:I ratio was estimated using the AUC of mEPSPs/mIPSPs (Methods). Overstimulation reduced the E:I ratio (Fig. 1d) due to increased mIPSP frequency (Fig. 1e and Supplementary Fig. 1g) and reduced mEPSP amplitude (Fig. 1f). Increased synaptic inhibition and decreased synaptic excitation were also evident in spontaneous EPSPs/IPSPs (sEPSPs/sIPSPs) (Supplementary Fig. 1j,m). Overstimulation did not modify mIPSP amplitude, sIPSP amplitude (Supplementary Fig. 1h, k), mEPSP frequency, sEPSP frequency (Supplementary Fig. 1i,l), action potential rheobase or membrane properties (Supplementary Table 7). Thus, overstimulation reduced the E:I ratio in young adult V1 through specific synaptic mechanisms.

To test for structural correlates of functional synaptic changes, we first measured dendritic spine size as a proxy for synaptic strength^3^. We tested whether overstimulation impacted spine size in L2/3 V1 neurons in *Thy1*-eGFP mice^34^ (Methods and Fig. 1g). To assay inhibitory circuitry, we used immunofluorescence approaches and labeled vesicular γ-aminobutyric acid transporter (VGAT) at *Thy1*-eGFP-positive neurons (Methods and Fig. 1g). Overstimulation decreased spine size, and this was blocked by the mGluR5 inhibitor MTEP^23,25^ (Fig. 1h). Reduced spine size was accompanied by increased density of VGAT puncta at L2/3 *Thy1*-eGFP^+^ neurons of overstimulated mice (Fig. 1i). VGAT and spine density were not modified by MTEP treatment (Fig. 1i and Supplementary Fig. 1n). Our results suggest that mGluR5-dependent synaptic weakening occurs following overstimulation.

We next asked if weakened excitatory synaptic strength is captured by multiplicative scaling factors, similarly to reports during downward firing rate homeostasis^22^. We used a standard approach^3,35^ to test if multiplicative values could scale control distributions down to measures made after overstimulation (Methods and Supplementary Fig. 1o–y). For mEPSP amplitude (Supplementary Fig. 1o,p) and spine size (Supplementary Fig. 1q,r), control values could be scaled to the overstimulation distribution. We next tested if overstimulation modified expression of the AMPA receptor subunit GluA2, the synaptic scaffolding molecules GRIP1 and HOMER1 and the Ca^2+^/calmodulin-dependent kinase eEF2K, as these synaptic molecules have been linked to both population-level multiplicative synaptic plasticity^3,36,37^ and long-term depression (LTD)^38,39^. Immunofluorescence experiments^3^ measured expression levels per unit of spine size of GluA2 (Supplementary Fig. 1s,t), GRIP1 (Supplementary Fig. 1u,v), HOMER1 (Supplementary Fig. 1w,x) and eEF2k (Supplementary Fig. 1y) at *Thy1*-eGFP^+^ dendrites in L2/3 of V1 in control and overstimulated young adult animals (Methods). GluA2 (Supplementary Fig. 1s,t), GRIP1 (Supplementary Fig. 1u,v) and HOMER1 (Supplementary Fig. 1w,x) decreased after overstimulation, and control distributions could be scaled to those measured after overstimulation (Supplementary Fig. 1s–x). However, the expression of eEF2k, a protein thought to be critical for mGluR5-dependent LTD^39^, was not modified by overstimulation (Supplementary Fig. 1y). In summary, functional (Supplementary Fig. 1o,p), structural (Supplementary Fig. 1q,r) and molecular (Supplementary Fig. 1s–x) weakening of excitatory synaptic strength after overstimulation is captured by multiplicative scaling values, suggesting shared features with synaptic downscaling-like plasticity reported by others^22,25^ (Discussion).

### Age-related dysregulation of response to overstimulation

**Fig. 2:**
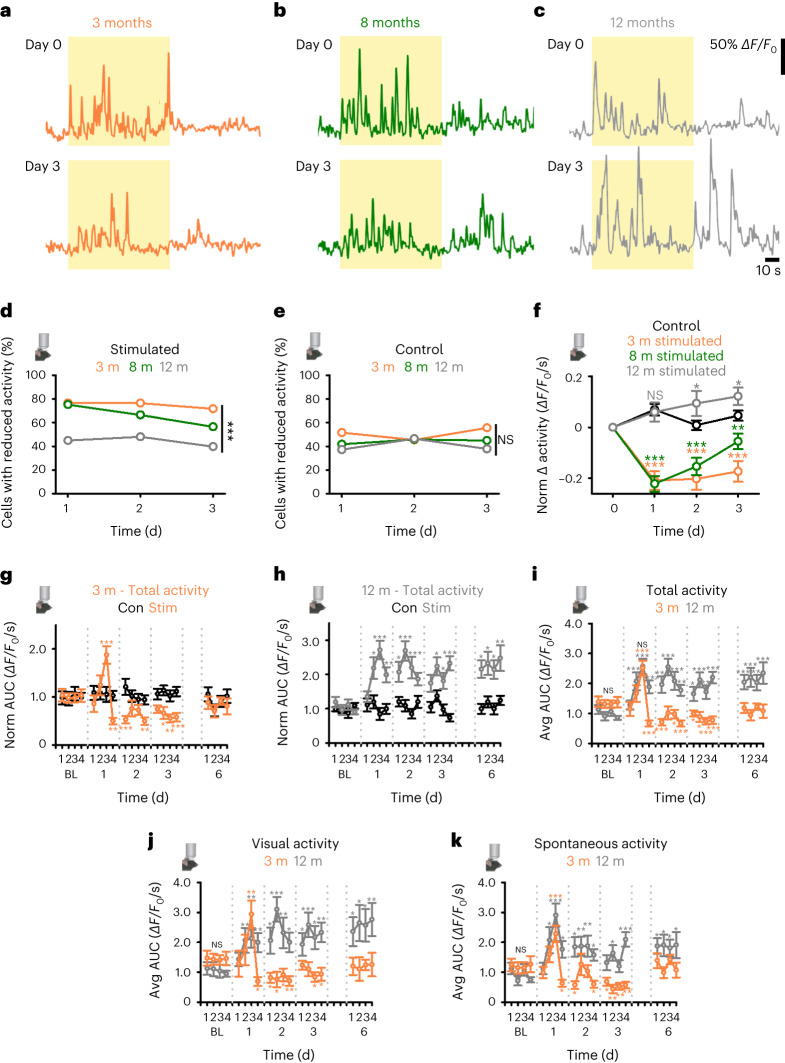
Age-related changes in the functional plasticity of the neuronal activity response to sensory overstimulation. **a**–**c,** *ΔF/F*_0_ Calcium traces from V1 L2/3 neurons before (day 0/baseline (BL), top) and after 3 d of overstimulation (day 3, bottom) in anesthetized animals at 3 months (**a**, orange), 8 months (**b**, green) and 12 months (**c**, gray). Yellow background depicts light stimulation; white background denotes spontaneous activity. Scale bars, 50% *ΔF/F*_0_ and 10 s. **d**,**e**, Neurons with reduced response in overstimulated (**d**) and control (**e**) mice at 3 months (orange), 8 months (green) and 12 months (gray) (Chi-squared test, Stim 3 months versus Stim 12 months, *P* < 0.001; Chi-squared test, Con 3 months versus Con 12 months, *P* = 0.227). **f**, Normalized change (to day 0, BL) in total calcium-mediated neuronal activity (*ΔF/F*_0_ integral per second) for overstimulated mice at 3 months (orange), 8 months (green) and 12 months (gray) and pooled control groups (black) (see Supplementary Fig. 2 for individual age-matched comparisons). Asterisks or NS represent comparisons to control for each day. (Two-way ANOVA, Con versus Stim, day 1: 3 months, *P* < 0.001; 8 months, *P* < 0.001; 12 months, *P* = 0.791; day 2: 3 months, *P* < 0.001; 8 months, *P* < 0.001; 12 months, *P* = 0.015; day 3: 3 months, *P* < 0.001; 8 months, *P* = 0.005; 12 months, *P* = 0.029). **g**,**h**, Normalized (to average BL) activity (*ΔF/F*_0_ integral per second; total activity) for overstimulated (3 months, orange, **g**; 12 months, gray, **h**) and age-matched anesthetic control (non-stimulated) mice (black) across more fine-scale time course. **i**, Average activity (*ΔF/F*_0_ integral per second; total activity) for overstimulated mice at 3 months (orange) and 12 months (gray) across fine-scale time course. **j**,**k**, Visual (**j**) and spontaneous (**k**) activity (*ΔF/F*_0_ integral per second) for overstimulated mice at 3 months (orange) and 12 months (gray). Absence of orange (3 months) or gray (12 months) asterisks denote no significant differences from BL (**g**–**k**), while NS denotes no significant differences between 3- and 12-month-old animals in BL sessions or in the acute response (S3) to overstimulation (**i**–**k**). Data were obtained from 19 animals and 31 regions for **d**–**f** and 6 animals (3 months Stim: 73; 3 months Con: 136; 12 months Stim: 142; 12 months Con: 150 neurons) for **g**–**k**. *P < 0.05, **P < 0.01, ***P < 0.001. Two-sided tests were used throughout. Detailed statistics are reported in Supplementary Table 2. Data are presented as the mean ± s.e.m. Source data

We next asked if the neuronal activity response to overstimulation (Fig.1c and Supplementary Figs. 1c and 2f) changed with age by repeating our in vivo calcium imaging experiments in adult mice aged 3–12 months (Methods and Fig. 2a–c). We found an age-related decline in neurons expressing reduced activity after overstimulation (Methods, Fig. 2d,e and Supplementary Fig. 2a). In 3-month-old mice, ~70% of neurons had reduced activity after overstimulation (Fig. 2d and Supplementary Fig. 2a). This percentage declined with age, reaching values similar to those of controls by 12 months (~45%; Fig. 2d,e and Supplementary Fig. 2a). At the population level, animals aged 3–8 months showed reduced activity after day 1 of overstimulation, while 12-month-old animals did not (Fig. 2f). In older animals (aged 8–12 months), activity increased across further overstimulation sessions (Fig. 2f); as a result, in 12-month-old animals, activity was greater than control values on day 3 of overstimulation (Fig. 2f and Supplementary Fig. 2b). In contrast, a consistent reduction in activity was maintained across overstimulation sessions in 3-month-old animals (Fig. 2f and Supplementary Fig. 2b). Activity levels in age-matched anesthetic control animals were stable during imaging (Fig. 2g,h, Supplementary Fig. 2b and Supplementary Table 8). However, in agreement with previous work^8,29^, the percentage of neurons with high-frequency calcium-mediated neuronal activity increased with age (Methods and Supplementary Fig. 2c).

We investigated more fine-scale age-dependent activity changes recording from active neurons after each overstimulation session (Fig. 2g–k and Supplementary Fig. 2d–s). Both young animals (3 months) and animals in later adulthood (12 months) showed an acute (S1–3, day 1) elevation in total activity (Fig. 2g–i) that was not evident in anesthetic controls (Fig. 2g,h). The acute elevation was evident, and similar, for both age groups, for measures of visually evoked (Fig. 2j) and spontaneous activity (Fig. 2k) but occurred slightly earlier in older animals (Fig. 2h–k and Supplementary Fig. 2f–o). After overstimulation, twelve-month-old animals had total activity values greater than baseline (Fig. 2h–k and Supplementary Fig. 2g), with similar changes in visually evoked (Fig. 2j and Supplementary Fig. 2i,k) and spontaneous (Fig. 2k and Supplementary Fig. 2m,o) activity. Increased activity was driven by changes in *ΔF/F*_0_ amplitude, frequency or both (Supplementary Fig. 2g,i,k,m,o and Supplementary Table 8). Age-related differences after overstimulation may be due to slower rates of adaptation in older animals. However, this was not the case, as animals in later adulthood continued to have elevated activity levels even after extended (6 d) periods of overstimulation (Methods, Fig. 2h–k and Supplementary Fig. 2g,i,k,m,o). Activity levels in young adults returned to baseline values following prolonged overstimulation (Fig. 2g,i,j,k and Supplementary Fig. 2f,h,j,l,n). In late adults, activity levels remained elevated 3 d after cessation of overstimulation but did decline relative to measures over days 3–6 (Supplementary Fig. 2p,q). Overstimulation did not modify orientation (Supplementary Fig. 2r) or direction (Supplementary Fig. 2s) selectivity in young or late adult animals, suggesting basic features of sensory encoding are unaffected despite elevated activity. Our results find the adaptive response to overstimulation becomes dysregulated between early and late adulthood (Fig. 2a–k and Supplementary Fig. 2f–o), across a period when more neurons exhibit high-frequency activity (Supplementary Fig. 2c). Furthermore, the overstimulation response in animals in later adulthood is tenacious and not due to slower adaptive plasticity.

### Age-related dysregulation of overstimulation-driven synaptic plasticity

**Fig. 3:**
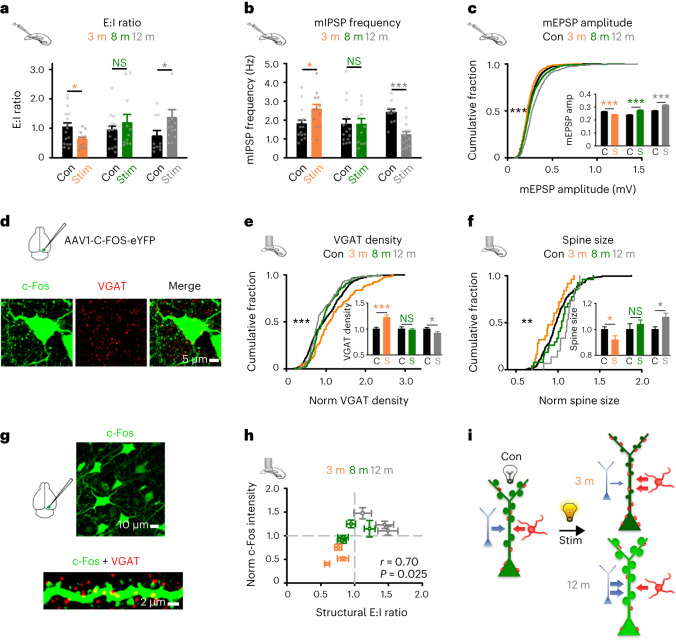
Age modifies the synaptic plasticity response to overstimulation. **a**–**c**, E:I ratio (Welch’s *t*-test, Con versus Stim, 3 months: *P* = 0.022; MWRST, 8 months: *P* = 0.795; MWRST, 12 months: *P* = 0.038) (**a**), mIPSP frequency (Hz) (*t*-test, Con versus Stim, 3 months: *P* = 0.030; MWRST, 8 months: *P* = 0.959; *t*-test, 12 months:, *P* < 0.001) (**b**) and mEPSP amplitude (mV) (MWRST, Con versus Stim, 3 months: *P* < 0.001; MWRST, 8 months: *P* < 0.001; MWRST, 12 months: *P* < 0.001) (**c**) for overstimulated mice at 3 (orange), 8 (green) and 12 (gray) months after 3 d of overstimulation versus age-matched controls (black). **d**, Images showing a c-Fos-positive V1 L2/3 neuron (left, green), VGAT puncta (middle, red) and a merged image (right) of mice expressing AAV1-C-FOS-eYFP. Scale bar, 5 µm. **e**, Density of VGAT puncta at c-Fos-positive neurons for overstimulated mice at 3 (orange), 8 (green) and 12 (gray) months normalized to age-matched controls (black) (MWRST, Con versus Stim, 3 months: *P* < 0.001; MWRST, 8 months: *P* = 0.497; *t*-test, 12 months: *P* = 0.043). **f**, Spine size from c-Fos-positive dendrites in overstimulated mice at 3 (orange), 8 (green) and 12 (gray) months normalized to age-matched controls (black) (*t*-test, Con versus Stim, 3 months: *P* = 0.032; *t*-test, 8 months: *P* = 0.495; *t*-test, 12 months: *P* = 0.015). **g**, Example of c-Fos-positive neurons in V1 (top), and colocalization of VGAT puncta with c-Fos-positive dendrite (bottom). Scale bars, 10 µm (top) and 2 µm (bottom). **h**, Correlation between dendritic structural E:I ratio and c-Fos intensity per dendrite across stimulated age groups; each point represents an animal average ± s.e.m. (3 months, orange; 8 months, green; 12 months, gray; Pearson correlation, E:I ratio versus c-Fos intensity, *r* = 0.70, *P* = 0.025). **i**, Schematic showing overstimulation results in lower c-Fos levels (green), reduced spine size and increased VGAT (red dots) density in 3-month-old animals, while in 12-month-old animals overstimulation drove higher c-Fos levels, larger spines and decreased VGAT density. Data were obtained from 54 animals for **a**–**c**, 18 animals for **e** and **f** and 10 animals for **h**. *P < 0.05, **P < 0.01, ***P < 0.001. Two-sided tests were used throughout. Detailed statistics are reported in Supplementary Table 3. Cumulative distributions and comparisons for overstimulated groups and age-matched controls for **c**, **e** and **f** are shown in Supplementary Fig. 3. Data are presented as the mean ± s.e.m., with individual data points (gray dots), or as a cumulative distribution. Source data

We next tested whether changes in the plasticity mechanisms identified in young adult animals accounted for the age-related response to overstimulation. We performed electrophysiological recordings of mEPSPs/mIPSPs from L2/3 excitatory neurons in control and overstimulated mice across our age range and calculated the E:I ratio (Methods and Fig. 3a). Older animals (8–12 months) exhibited a different synaptic plasticity response to overstimulation than young adults (3 months; Fig. 3a). By 12 months, excitatory neurons exhibited an increased E:I ratio after overstimulation relative to controls, rather than the decreased E:I ratio measured in young adults (Fig. 3a). Age impacted the inhibitory and excitatory synaptic plasticity response to overstimulation (Fig. 3a–c and Supplementary Fig. 3a–d). In 12-month-old mice, mIPSP frequency was no longer increased after overstimulation, and instead showed a decrease relative to controls (Fig. 3b and Supplementary Fig. 3b). This effect occurred in concert with a progressive age-related increase in mEPSP amplitude after overstimulation, to values greater than control levels in 8-month-old and 12-month-old overstimulated mice (Fig. 3c and Supplementary Fig. 3c,d). Similar changes in synaptic excitation and inhibition were found after overstimulation in measures of spontaneous EPSPs/IPSPs from 12-month-old animals (Supplementary Fig. 3g,h). We saw no age-related effects on mEPSP frequency (Supplementary Fig. 3e), mIPSP amplitude (Supplementary Fig. 3f), rheobase or passive membrane properties (Supplementary Table 9) after overstimulation, suggesting age impacts specific synaptic mechanisms.

Overstimulation drives elevated activity (Fig. 2a–k) and an increased synaptic E:I ratio in older animals (Fig. 3a–c). We tested for structural synaptic correlates at neurons recently active in vivo (Methods and Fig. 3d). The immediate early gene *cFos* can approximate in vivo neuronal activity and allow recently active neurons to be fluorescently tagged^2,40^. We therefore adopted two c-Fos-based strategies. First, an in vivo viral expression approach that labeled the entire neuron, and second, an ex vivo staining method to validate our viral strategy. The virally expressed C-FOS-eYFP construct labeled the dendritic and axonal arborization of neurons expressing c-Fos (Methods and Fig. 3d) and reported similar c-Fos expression to immunofluorescence measures (Supplementary Fig. 3i). We prepared brain slices from animals expressing C-FOS-eYFP and measured VGAT puncta and dendritic spines at c-Fos-positive neurons in 3-month-old, 8-month-old and 12-month-old control and overstimulated animals (Fig. 3d–h and Supplementary Figs. 1f and 3j–w). We observed increased VGAT density with age in control animals, suggesting inhibitory synaptogenesis persists into late adulthood (Supplementary Fig. 3j). In 3-month-old mice, overstimulation resulted in increased VGAT density at c-Fos-positive neurons (Fig. 3e and Supplementary Fig. 3k). The inhibitory response to overstimulation declined with age (Fig. 3e and Supplementary Fig. 3k–m), so that 12-month-old overstimulated mice exhibited a small but significant reduction in VGAT density relative to controls (Fig. 3e and Supplementary Fig. 3m). In 3-month-old overstimulated animals, increased VGAT density was evident at neurons expressing both low and higher levels of c-Fos (Supplementary Fig. 3n–p and Methods). However, in overstimulated late adult animals, reduced VGAT density was specific to neurons with higher levels of c-Fos expression (Supplementary Fig. 3q), suggesting that loss of VGAT puncta in older animals may contribute to the elevated neuronal activity following overstimulation (Supplementary Fig. 3q and Fig. 2a–k). We next measured dendritic spines at c-Fos-positive branches (Fig. 3f and Supplementary Fig. 3r–t), and found a progressive age-related increase in spine size after overstimulation (Fig. 3f and Supplementary Fig. 3r–t), without a change in density (Supplementary Fig. 3u). Spine density and size were stable across our chosen age range in control animals (Supplementary Fig. 3v–w).

Increased excitatory synaptic strength without a balanced change in inhibition may drive the heightened neuronal activity we measured in vivo in late adult animals after overstimulation (Fig. 2a–k). We tested the correlation between synaptic plasticity (structural E:I ratio; Methods) and estimates of neuronal activity (using c-Fos) on a cell-by-cell basis. We found an age-related increase in c-Fos intensity after overstimulation positively correlated with the structural synaptic E:I ratio (Fig. 3h). Our results suggest that in 12-month-old animals, overstimulation increases the E:I ratio, as opposed to the decrease seen in young adult animals (Fig. 3a). The increased E:I ratio is due to reduced inhibitory synaptic plasticity and a shift from excitatory synaptic weakening to strengthening after overstimulation (Fig. 3i).

### Age-related dysregulation of in vivo dendritic spine plasticity

**Fig. 4:**
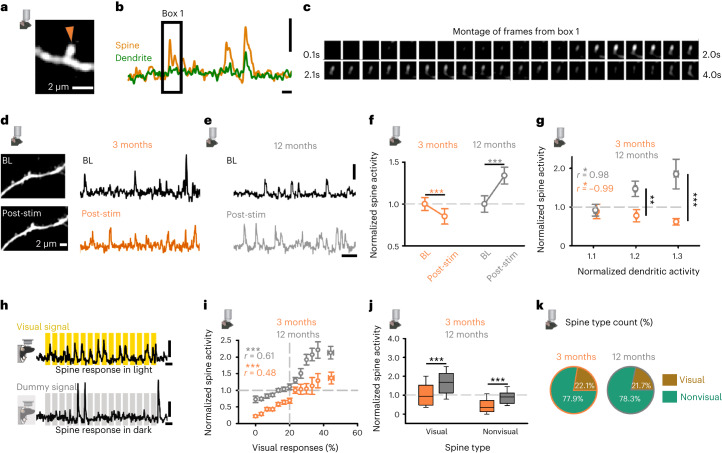
Age modifies functional plasticity response of dendritic spines in vivo. **a**–**c**, Spine from 2-P in vivo calcium imaging in anesthetized mice (**a**). *ΔF/F*_0_ calcium trace from spine (orange) and adjacent dendrite (green) in **a** (**b**). Montage of frames (collected at 30 Hz, shown at 10 Hz) from ‘Box 1’ in **b** (**c**). Scale bars, 2 µm (**a**), 50% *ΔF/F*_0_ and 2 s (**b**). **d**,**e**, Longitudinal *ΔF/F*_0_ calcium traces of spine activity for 3 months (**d**, orange) and 12 months (**e**, gray) animals at BL (top, black) and after overstimulation (post-stim, bottom, orange, gray). Scale bars, 2 µm (**d**), 50% *ΔF/F*_0_ and 10 s (**e**). **f**, Normalized (to baseline) spine activity (*ΔF/F*_0_ integral) at BL and after overstimulation (post-stim) for mice at 3 months (left, orange) and 12 months (right, gray). (Wilcoxon signed-rank test, BL versus post-stim, 3 months: *P* < 0.001; 12 months: *P* < 0.001). **g**, Normalized (to BL) change in activity (*ΔF/F*_0_ integral) of spine and home dendrite for branches with increased activity in animals at 3 m (orange) and 12 m (gray) animals (Pearson correlation, normalized dendritic activity versus norm spine activity, 3 months *r* = −0.99, *P* = 0.012; 12 months *r* = 0.98, *P* = 0.021). **h**, *ΔF/F*_0_ calcium traces of spine activity during overstimulation-like visual stimuli (top, yellow), or in the dark, aligned with time-matched dummy visual stimuli (bottom, gray). Scale bars, 50% *ΔF/F*_0_ and 4 s. **i**, Spine visual responsivity to overstimulation against normalized (to BL) spine activity (*ΔF/F*_0_ integral) after overstimulation for mice at 3 months (orange) and 12 months (gray). The vertical dashed line denotes threshold for visual responsivity (Supplementary Fig. 4f), and the horizontal dashed line denotes no change in activity following overstimulation. (Pearson correlation, visual responses versus normalized spine activity, 3 months *r* = 0.48, *P* < 0.001; 12 m *r* = 0.61, *P* < 0.001). **j**, Normalized (to BL) change in spine activity (*ΔF/F*_0_ integral) for visual (left) and nonvisual (right) spines in mice at 3 months (orange) and 12 months (gray). (MWRST, 3 months versus 12 months, Visual: *P* < 0.001; nonvisual: *P* < 0.001). **k**, Percentage of visual (brown) and nonvisual (green) spines for mice at 3 months (left, orange) and 12 months (right, gray). (Chi-squared test, 3 months vs 12 months, visual: *P* = 0.976; Non-visual, *P* = 0.963). Data from 8 animals (13 regions) for **a**–**k**. *P < 0.05, **P < 0.01, ***P < 0.001. Two-sided tests were used throughout. Detailed statistics are reported in Supplementary Table 4. Data are presented as the mean ± s.e.m., or as the median and interquartile range (in the box plots, the box represent quartiles and whiskers indicate the 10th and 90th percentiles). Source data

We investigated the functional identity of synapses driving the age-related overstimulation response from population-level weakening to synaptic strengthening. We used in vivo 2-P imaging to measure calcium signals at dendritic spines of L2/3 excitatory cells before and after overstimulation in young (3 months) and late (12 months) adult mice (Methods and Fig. 4a–e). We collected a baseline session (Fig. 4d,e) and then imaged after a day of overstimulation (four sessions, post-stimulation; Methods and Fig. 4d,e). We extracted spine responses using published methods^41,42^ (Supplementary Fig. 4a–c) and compared activity before and after overstimulation (Fig. 4d–f).

We measured the population response of calcium signals at spines (Fig. 4f and Supplementary Fig. 4d–e) during light-flicker stimuli similar to the home-cage overstimulation. In 3-month-old animals, overstimulation decreased population-level spine activity in vivo (Fig. 4f and Supplementary Fig. 4d). In contrast, overstimulation increased spine activity in 12-month-old animals (Fig. 4f and Supplementary Fig. 4e). These results mirrored electrophysiological and spine size measures (Figs. 1f,h and 3c,f) and suggest the overstimulation response shifts from population-level excitatory synaptic weakening to strengthening with age in vivo.

To better understand how functional spine plasticity relates to neuronal activity in vivo, we compared the spine plasticity response to changes in activity at the adjacent dendrite following overstimulation. Dendritic activity measured in this way is thought to approximate global cellular activity^3^. Similar dendritic activity changes were accompanied by markedly different changes in spine activity after overstimulation in young and late adult animals (Fig. 4g). In young animals, spine activity was reduced at branches with elevated activity after overstimulation (Fig. 4g). In contrast, the activity of spines in older animals was positively correlated with changes in the activity of dendritic branches (Fig. 4g). These results suggest that similar changes in dendritic activity are accompanied by synaptic weakening in young adults and strengthening in older animals.

We tested whether the shift from synaptic weakening to strengthening occurred at all spines, or those most sensitive to overstimulation (Fig. 4h). We measured spine responsivity during the overstimulation stimulus and compared to the number of events during spontaneous activity^2,43^ (Methods, Fig. 4h and Supplementary Fig. 4f). We defined visually responsive spines as those with time-locked responses to >20% of visual stimulus trials and classified remaining spines as nonvisual (that is, non-responsive to the overstimulation stimulus; Supplementary Fig. 4f). Both visual and nonvisual spines were present on all imaged branches and did not exhibit clustering (Supplementary Fig. 4g,h).

In young and late adult animals, visual responsivity positively correlated with the change in synaptic strength after overstimulation (Fig. 4i,j). In young adult animals, most spines exhibited reduced activity after overstimulation (435/531, 81.9%) (Fig. 4i). In late adult animals, ~30% fewer spines exhibited a weakened response (218/428, 50.9%; Fig. 4i). Weakening after overstimulation was most prominent at nonvisual spines in both young (332/366, 90.7%) and late adult (188/303, 62.1%) animals (Fig. 4i). However, the degree of weakening expressed by nonvisual spines after overstimulation was more prominent in younger animals (Fig. 4i,j). Strengthening at visually responsive spines was much greater in older (95/125, 76.0%) than younger (62/165, 37.6%) animals after overstimulation (Fig. 4i). Age-related effects couldn’t be explained by baseline spine activity (Supplementary Fig. 4i) or the proportion of spine types (Fig. 4k). Our results suggest that reduced weakening at nonvisual spines combines with greater sensory-driven strengthening at spines responsive to overstimulation to drive the population-level increase in excitatory synaptic strength in older animals after overstimulation (Fig. 4f).

### Identified mechanisms account for age-related overstimulation response

We next modeled the overstimulation response using plastic excitatory and inhibitory mechanisms and feedforward excitatory input (Methods and Supplementary Fig. 4j). The plasticity of synaptic weights was governed by Hebbian learning and modulated by inhibition and homeostatic adaptation, which scaled excitatory synaptic weights (Supplementary Fig. 4j). This model was compared to a late adulthood simulation where excitatory homeostasis and inhibitory plasticity were reduced during the overstimulation phase (Methods and Supplementary Fig. 4j).

In the young adult simulation, overstimulation and Hebbian plasticity strengthened weights between neurons with a coincident increase of presynaptic and postsynaptic activity and weakened weights between neurons with uncorrelated activity. The impact of increased inhibition and excitatory synaptic homeostasis gave weight changes (Supplementary Fig. 4k) aligned with experimental observations (Fig. 4i). Reducing excitatory synaptic homeostasis and the plasticity of inhibition resulted in weight changes akin to those measured in older animals (Fig. 4i,j and Supplementary Fig. 4k,l). Specifically, our results show increased synaptic strengthening in the most visually responsive inputs and reduced synaptic weakening in less visually responsive inputs (Supplementary Fig. 4k,l).

We investigated the contribution of excitatory and inhibitory plasticity by changing each process independently (Supplementary Fig. 4m–r). Starting conditions matched our late adulthood simulation (Supplementary Fig. 4m,p), and then inhibitory (Supplementary Fig. 4m–o,s) and excitatory (Supplementary Fig. 4p–r,t) plasticity mechanisms were progressively reinstated, and the impact of overstimulation on synaptic weights was assessed. Increasing inhibitory plasticity, during reduced excitatory homeostasis, reduced the strengthening of all weights, but with most prominent changes at more visually responsive weights (Supplementary Fig. 4m–o,s). Increasing levels of excitatory synaptic homeostasis, during reduced inhibitory plasticity, led to a weakening of all weights relative to the late adulthood simulation (Supplementary Fig. 4p–r), with most prominent changes at the least visually responsive inputs (Supplementary Fig. 4t). These results were explained by Hebbian plasticity having most impact on the highly visual synapses, which have highest covariance with postsynaptic activity (Methods and equation (3)). As a result, increasing inhibitory plasticity most strongly suppresses the potentiation of visual synapses. In contrast, the homeostatic term weakens all synapses, but weakening of nonvisual synapses is more pronounced, because Hebbian potentiation at these synapses is small. In summary, both plasticity processes are required to capture features of our experimental findings.

### Age modifies assembly plasticity to overstimulation

**Fig. 5:**
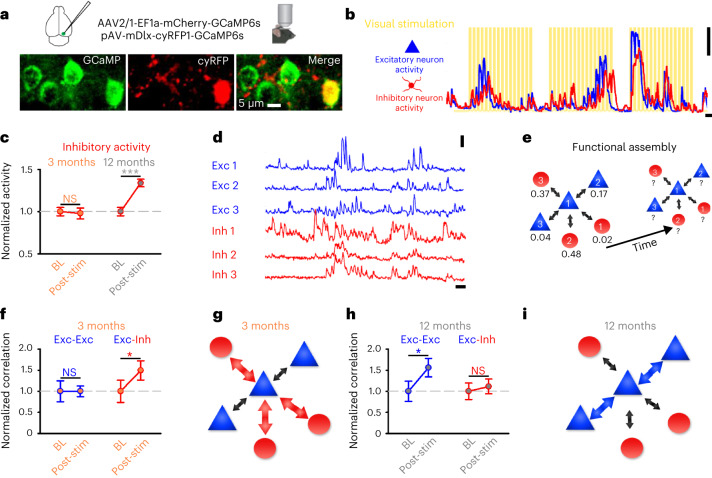
Age modifies network plasticity response to sensory overstimulation in vivo. **a**, Co-injection strategy (top) and example region (bottom) showing GCaMP6s positive excitatory and inhibitory neurons (left, green), inhibitory neurons expressing cyRFP under the mDlx enhancer (middle, red) and a merged image of both channels (right; Methods). Scale bar, 5 µm. **b,** *ΔF/F*_0_ calcium traces from excitatory (blue) and inhibitory (red) neurons in response to visual stimulation (yellow). Scale bars, 100% *ΔF/F*_0_ and 10 s. **c**, Inhibitory (red) neuronal activity (*ΔF/F*_0_ integral) in 3-month-old (left, filled orange) and 12-month-old (right, filled gray) anesthetized mice following overstimulation (post-stim) normalized to BL (Wilcoxon signed-rank test, inhibitory activity, BL versus post-stim, 3 months: *P* = 0.732; Paired *t*-test, 12 months: *P* < 0.001). **d**, Representative *ΔF/F*_0_ calcium traces for excitatory (blue) and inhibitory neurons (red) from an imaged region. Scale bars, 100% *ΔF/F*_0_ and 10 s. **e**, Diagram depicting functional assembly association changes monitored over time for an individual excitatory neuron (1) between local excitatory (blue, 2 and 3) and inhibitory (red, 1, 2 and 3) neurons. The numbers inside cells correspond to the traces in **d**, while the numbers below the cells depict functional association strength values. **f**,**h**, Normalized (to BL) change in average correlation coefficient between excitatory and, either excitatory (Exc-Exc, blue), or inhibitory (Exc-Inh, red) cells after overstimulation (post-stim) compared to BL at 3 (**f**, orange) and 12 (**h**, gray) months. (Wilcoxon signed-rank test, Exc-Exc norm correlations, BL versus post-stim, 3 months: *P* = 0.465; 12 months: *P* = 0.046). (Paired *t*-test, Exc-Inh normalized correlations, BL versus post-stim, 3 months: *P* = 0.011; 12 months: *P* = 0.680). **g**,**i**, Diagram showing representative changes in functional assembly associations after overstimulation at 3 (**g**) and 12 (**i**) months, with the size of arrows denoting the change in average correlation strength after overstimulation. Data were obtained from 8 animals imaged at BL and after overstimulation (Post-stim). *P < 0.05, ***P < 0.001. Two-sided tests were used throughout. Detailed statistics are reported in Supplementary Table 5. Data are presented as the mean ± s.e.m. Source data

The age-related overstimulation response may have consequences for network-level plasticity^2^. We therefore conducted 2-P calcium imaging measurements in L2/3 of young (3 months) and late (12 months) adult V1 following co-injection with two viral constructs. The first construct used the mDlx enhancer system to label a heterogeneous population of inhibitory neurons with GCaMP6s and cyRFP (pAV-mDlx-Kz-f-cyRFP1-GSG-P2A-GCaMP6s-WPRE-pA; Fig. 5a and Supplementary Fig. 5a–c)^44^. The second construct labeled excitatory neurons with GCaMP6s and mCherry (AAV2/1-EF1a-mCherry-GSG-P2A-HIS-GCaMP6s-WPRE; Fig. 5a)^41^. Very few (0.37%, 2/539 neurons) labeled neurons expressed both constructs, suggesting separate neuronal populations were labeled (Supplementary Table 11)^44^. We then simultaneously imaged and separated the calcium-mediated neuronal activity of excitatory and inhibitory neurons sharing local cortical areas (Methods and Fig. 5a,b).

We investigated whether inhibitory neuronal activity following overstimulation was modified by age. Overstimulation did not modify the inhibitory population activity in young adults (Fig. 5c). However, inhibitory activity increased after overstimulation in older animals (Fig. 5c), but this did not dampen the activity of excitatory neurons (Supplementary Fig. 5d). Our earlier experiments found reduced VGAT density at excitatory neurons in older animals after overstimulation (Fig. 3e). We wondered whether a reduction in VGAT may occur at inhibitory neurons in older animals after overstimulation, possibly facilitating elevated inhibitory activity (Fig. 5c). We measured VGAT puncta at GAD-positive neurons in young and late adult animals (Methods and Supplementary Fig. 5e–h) and found the size (Supplementary Fig. 5e), but not density (Supplementary Fig. 5f), of VGAT puncta was reduced in late adult animals after overstimulation. In young adult animals, the size and density of VGAT puncta were similar to those at control levels in GAD-positive neurons after overstimulation (Supplementary Fig. 5g,h). Our results suggest that overstimulation may lead to weakening of synaptic inhibition onto inhibitory neurons in late adult animals, possibly contributing to the elevated inhibitory activity in vivo.

We investigated the age-related network-level response to overstimulation at assemblies of excitatory and inhibitory neurons (Fig. 5d–i), and estimated functional associations via pairwise calcium correlations (Methods and Fig. 5d,e)^2^. We considered neurons correlated if coefficients were significant and positive^2,42,43^. For each excitatory neuron, we calculated average pairwise correlation strength with other excitatory or inhibitory neurons in the local cortical region during visual stimulation (Methods and Fig. 5e). In young adult animals, overstimulation increased association strength between excitatory and inhibitory neurons but had little impact on association strength between only excitatory cells (Fig. 5f,g). In contrast, overstimulation increased association strength between excitatory neurons in late adult animals, but not between excitatory and inhibitory cells (Fig. 5h,i). Overstimulation did not impact associations between inhibitory neurons in either young or late adult animals (Supplementary Fig. 5i). Our results suggest that age modifies the network-level plasticity response of functional assemblies to overstimulation, so that rather than developing stronger associations with inhibitory cells, excitatory neurons in older animals become more strongly associated with each other.

### Overstimulation drives age-related disruption of cognition

**Fig. 6:**
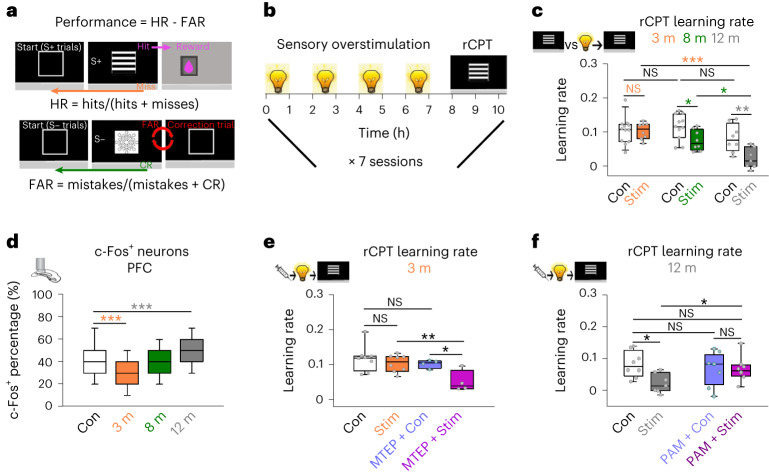
Sensory overstimulation drives age-related impairments in cognitive performance that are rescued by mGluR5 modulation. **a**, Schematic of the rCPT behavioral task involving discrimination between a rewarded (top, ‘S+’ grating icon) and unrewarded (bottom, ‘S−’ snowflake icon) visual stimuli. Equations describe main metrics to calculate task performance from which learning rates were calculated. **b**, Experimental timeline for mice that received overstimulation (light bulb icons) followed by rCPT testing (grating icon) during seven daily sessions. **c**, Learning rates for overstimulated mice at 3 months (orange), 8 months (green), 12 months (gray) and age-matched controls (white). (Two-way ANOVA, learning rates, Con versus Stim, 3 months: *P* = 0.951; 8 months: *P* = 0.019; 12 months: *P* = 0.002). **d**, Percentage of c-Fos-positive neurons in overstimulated mice at 3 (orange), 8 (green), 12 (gray) months and controls (white) in the prefrontal cortex (PFC). (Kruskal–Wallis one-way ANOVA, c-Fos percentage, Con versus 3 months: *P* < 0.001; Con versus 12 months: *P* < 0.001). **e**, Learning rates for 3-month-old mice that received no stimulation (Con, white), overstimulation (Stim, orange), MTEP and no stimulation (MTEP + Con, blue) or MTEP and overstimulation (MTEP + Stim, purple). (Two-way ANOVA, learning rates, Con versus Stim: *P* = 0.369; MTEP + Con versus MTEP + Stim: *P* = 0.025; Con versus MTEP + Con: *P* = 0.416; Stim versus MTEP + Stim: *P* = 0.009). **f**, Learning rates for 12-month-old mice that received no stimulation (Con, white), overstimulation (Stim, gray), the mGluR5 PAM (VU0409551) and no stimulation (PAM + Con, blue) or the mGluR5 PAM and overstimulation (PAM + Stim, purple). (Two-way ANOVA, learning rates, Con versus Stim: *P* = 0.010; PAM + Con versus PAM + Stim: *P* = 0.960; Con versus PAM + Con: *P* = 0.517; Stim versus PAM + Stim: *P* = 0.047). Data were obtained from 54 animals for **c**, 18 animals for **d**, 24 animals for **e** and 29 animals for **f**. *P < 0.05, **P < 0.01, ***P < 0.001. Two-sided tests were used throughout. Detailed statistics are reported in Supplementary Table 6. Data are presented as the median and interquartile range (in the box plots, the box indicates quartiles, and whiskers represent the 10th and 90th percentiles), with individual data points (gray dots). Source data

We next tested if overstimulation impacted cognitive performance at different ages (3–12 months) using a touchscreen task involving visual discrimination (rodent continuous performance task (rCPT))^45^ (Fig. 6a and Supplementary Fig. 6a). In this task, animals make a correct nose poke to a rewarded visual stimulus (Hit), withhold responses to unrewarded stimuli (correct rejection (CR)), fail to respond (miss) or respond to an unrewarded stimulus (mistake). These behaviors generate the hit rate (HR) ($$\mathrm{{HR}={Hits}/({Hits}+{Misses})}$$), false alarm rate (FAR) ($$\mathrm{{FAR}={Mistakes}/({Mistakes}+{Correct\; rejections})}$$) and performance ($${HR}-{FAR})$$ metrics. The slope of a linear fit to performance scores across sessions gave a learning rate (Methods, Fig. 6a and Supplementary Fig. 6b).

All ages (3–12 months) learnt the task (Fig.6a,c, Supplementary Fig. 6a,b and Table 6) and thus we tested for an interaction between overstimulation and cognitive performance. Animals received the overstimulation paradigm and were tested after a rest period (~1 h; Methods and Fig. 6b). Overstimulation did not impact rCPT performance in 3-month-old mice (Fig. 6c). However, as animals aged, overstimulation resulted in progressively worse performance and elevated c-Fos (Fig. 6c,d). The results could not be explained by age-related sensorimotor deficits, reward association or motivation (Supplementary Fig. 6c–e).

Young adult plasticity mechanisms may confer resilience to overstimulation by preventing elevated neuronal activity, which can impair rodent cognition^10^. In young cortex, we found excitatory synaptic weakening after overstimulation is mGluR5 dependent (Fig. 1h). We therefore administered the noncompetitive mGluR5 inhibitor MTEP to young adult mice, to block this form of excitatory synaptic weakening, as well as other mGluR5-dependent processes^46^ (Fig. 1h) and repeated the overstimulation and behavioral experiments (Methods and Fig. 6e). MTEP alone did not impact performance (Fig. 6e); however, MTEP plus overstimulation reduced performance of young adult animals (Fig. 6e). All animals showed similar reward latency (Supplementary Fig. 6f), correct choice latency (Supplementary Fig. 6g) and trial completion (Supplementary Fig. 6h). However, although MTEP alone did not modify these variables, MTEP plus overstimulation led to a small increase in the reward collection latency (Supplementary Fig. 6f) and decreased trial number (Supplementary Fig. 6h), suggesting a possible reduction in motivation.

We next asked if positive allosteric modulation of mGluR5 could reduce the negative impact of overstimulation on cognitive performance in older animals. We injected late adult animals with VU0409551 (refs. ^47,48^; Methods), a PAM of mGluR5 acting independently of NMDARs^48^, which stabilizes mGluR5 at the plasma membrane^47,48^. Treatment with VU0409551 alone did not impact rCPT performance in unstimulated older animals (Fig. 6f). However, the negative impact of overstimulation on cognitive performance in older animals was reduced if animals received VU0409551 (Fig. 6f). This suggests that certain PAMs of mGluR5 signaling may protect against the negative impact of overstimulation on cognition in older animals (Fig. 6f).

## Discussion

We find synaptic plasticity mechanisms triggered by sensory overstimulation in young adults are dysregulated in late adulthood. In young adult cortex, overstimulation results in excitatory synaptic weakening and elevated synaptic inhibition, reducing the synaptic E:I ratio and dampening neuronal activity. This is accompanied by weakening of dendritic spine activity and strengthened associations between excitatory and inhibitory neurons after overstimulation in vivo. In contrast, overstimulation led to excitatory synaptic strengthening, modified network plasticity and a failure to recruit increased synaptic inhibition in late adult animals. Overstimulation also disrupted cognitive performance in older but not younger animals. The negative impact of overstimulation on cognition could be activated by blocking mGluR5-dependent processes in young adult animals and alleviated by a PAM of mGluR5 signaling in older animals. We find specific plasticity mechanisms are associated with age-related dysregulation of neuronal microcircuit homeostasis, and that the age-related response to sensory overstimulation impacts cognitive performance.

### Age-related impairments in plasticity processes

Aging may cause a general decline in plasticity processes; alternatively, specific forms of plasticity may be more vulnerable to aging^5^. We asked whether specific synaptic plasticity processes, triggered by elevated neuronal activity, exhibit an age-related decline, focusing on early-to-late adulthood time points to capture onset of mechanistic changes in plasticity^5^. We find age-related plasticity dysregulation can be unmasked by overstimulation. Age-related deficits occurred in two specific processes, namely, population-level excitatory synaptic weakening and increased synaptic inhibition onto excitatory neurons. Dysregulation of these mechanisms meant that repeated overstimulation drove elevated neuronal activity in older animals. Our work is supported by others reporting age-related neuronal hyperactivity can impair neural circuit function^49^, have negative consequences for cognition^10^ and occur across brain regions and species, including humans^8,10–14,29,30,50^.

### Alignment with theories of plasticity

Age-related failures in regulatory plasticity may have consequences for other forms of plasticity, or trigger compensatory processes^51^. In addition, multiple forms of homeostatic and Hebbian plasticity may be driven by overstimulation^23,52,53^. Our modeling found age-related plasticity effects were consistent with dysregulated homeostasis, leading to unconstrained Hebbian synaptic strengthening. However, such synaptic strengthening in older animals may occur due to an age-related shift in the synaptic potentiation threshold^52^. This scenario aligns with metaplasticity theories^52^. In fact, work in younger animals (postnatal day (P) 21–35) found excitatory synaptic weakening after sensory manipulation follows metaplasticity rules^23,52^. However, we found activity changes in young adult animals did not involve classical NMDAR-dependent plasticity mechanisms. Furthermore, metaplasticity theories do not fully capture the inhibitory synaptic changes we measured in older animals, although there are few studies in this area. The strengthening of spines in older animals may involve an independent plasticity process. Alternatively, the population-wide weakening we measured may be a global effect acting against the synaptic strengthening that would occur in its absence. Future work may consider how multiple plasticity mechanisms are triggered, both in adulthood and later senescence. For example, one study on sensory deprivation in juvenile mice (P24–30) found NMDAR-dependent and NMDAR-independent plasticity in response to the level of activity manipulation^53^.

Previous work describes homeostatic adaptation during sensory-evoked activity, typically following sensory deprivation^54–57^. Few studies have investigated the response to sensory-driven increases in activity, and those that have typically induce deprivation first^22,23^. We find sensory-evoked and spontaneous activity show an acute increase, then downward adaptation in young adults, with both phases independent of NMDARs. Overstimulation also reduced the E:I ratio in young adult animals, via mGluR5-dependent weakening of population-level excitatory synaptic strength and increased synaptic inhibition. Functional, structural and molecular measures of excitatory synaptic weakening occurred at the population level and were captured by multiplicative scaling rules, consistent with previous work^3,22,35^. Population-level excitatory synaptic weakening is dependent on dephosphorylation of the GluA2 AMPA receptor subunit, via Homer1a–mGluR1/mGluR5-mediated signaling cascades^18,23,25^. We found reduced GluA2, GRIP1 and HOMER1 expression in young adults following overstimulation. A key molecular feature of mGluR5-dependent LTD (eEf2k expression) was unaffected by overstimulation in young adult animals^39^. Together, the molecular, synaptic and cellular changes are consistent with homeostatic adaptation^20,21,25^. However, we cannot exclude that other non-NMDAR, mGluR5-dependent processes may weaken excitatory synapses and work in concert with increased synaptic inhibition.

### Possible molecular mechanisms

In older animals, overstimulation led to increased neuronal activity and a reduced inhibitory plasticity response. Brain-derived neurotrophic factor^58^ and nitric oxide signaling^59^ are known to increase inhibitory synaptic drive after elevated excitatory spiking. Brain-derived neurotrophic factor signaling is driven by activity-dependent transcription factors, such as *Npas4*, which coordinate E:I balance and decline with age^60,61^. The Npas4–Homer1a signaling pathway mediates homeostatic plasticity mechanisms^62^ and, in aged mice, elements of this pathway decrease^63^. Recruitment of synaptic inhibition may also be modified by age-related changes in synaptic adhesion proteins, such as neuroligins, which recruit inhibitory synapses onto excitatory neurons^64^ and show alterations during aging^65^. Future work may investigate if the inhibitory response is affected by age-related alterations to such molecular processes.

### Time course of plasticity processes

The plasticity time course likely depends on age, manipulation and studied system. In juvenile animals, homeostatic plasticity involving cellular or synaptic changes occurs 24–72 h after deprivation^66–68^. Elevated activity, following eyelid closure then reopening, also triggers plasticity across 24–48 h (ref. ^22^). In adults, retinal lesions drive upscaling within 24 h, with in vivo recovery of activity evident 18 h after deprivation^69^. Such approaches also trigger upscaling-like plasticity at inhibitory neurons^3^ by 6 h (ref. ^70^). Adult monocular enucleation drives spine size increases (8–24 h) preceding recovery of activity (48 h)^2,3^. Molecular changes underpinning scaling can occur within 4 h of pharmacological challenge in culture systems^71^. We find relatively fast functional spine changes, occurring after overstimulation (~10 h). Overstimulation may drive plasticity at a different rate to deprivation paradigms. For example, whisker overstimulation (24 h) drives rapid increases in inhibitory synapses^72^. In addition, juvenile mice (P21–35) subjected to dark exposure followed by light reexposure showed weakened synaptic strength within 2 h^23^. Future work may test plasticity timescales across manipulations of differing severity^53^.

### Overstimulation and cognition

Age-related impairments in Hebbian plasticity can reduce cognitive performance^73^. How dysregulated homeostasis impacts cognition is less clear^26^. Overstimulation impaired cognitive performance in older, but not younger, animals. Eight-month-old animals showed a loss of E:I plasticity, without elevated E:I ratios, but still exhibited reduced performance after overstimulation. This, in combination with measures in 12-month-old animals, suggests age dysregulates plasticity. Short bouts of visual stimulation (1 h per day at 40 Hz) can improve cognition in Alzheimer’s disease models^74^. We used prolonged and repeated overstimulation to drive a strong plasticity response. Thus, one possibility is that the duration of overstimulation has different effects on plasticity and, ultimately, cognition. For example, a shorter repeated stimulus may drive Hebbian-like potentiation and/or neuroprotective processes^74,75^, while prolonged stimulation could upregulate homeostatic plasticity.

Age-related neuronal hyperactivity correlates with impaired rodent cognition^10^. Antiepileptic drugs improve cognition in aged rodents and humans with mild cognitive impairment^76^. We found blockade of mGluR5-dependent processes rendered young adult animals susceptible to the negative impact of overstimulation on cognition, while a PAM of mGluR5 reduced the negative impact of overstimulation on cognition in older animals. Thus, mGluR5-dependent mechanisms may modulate the interaction between overstimulation and cognition. However, further work is required to determine if mGluR5 is directly involved in the age-related alteration to overstimulation. We propose that age-related impairments in regulatory homeostatic plasticity processes, when challenged with sensory overstimulation, promote elevated cortical activity and may subsequently impair cognition.

## Methods

### Animals

Experiments were conducted according to the United Kingdom Animals (Scientific Procedures) Act 1986 and approved by the UK Home Office. We used male and female adult (P60–360) C57BL/6J mice, or adult (P60–100) *Thy1*-eGFP mice on a C57BL/6J background (JAX 007788, The Jackson Laboratory; Fig. 1g–i and Supplementary Fig. 1n,q–y). Ages were defined as: young adult (3 months), mature adult (8 months) and late adult (12 months)^5^. Mice were matched for sex and age within experimental groups, but randomly assigned to conditions, and housed (temperature, 21 ± 2 °C; humidity, 55% ± 10%) with littermates on a 12 -h light–dark cycle. Longitudinal imaging data were matched for time within that animal’s light cycle. All mice had access to water and food ad libitum, except during the rCPT (‘Behavioral experiments’). For animal numbers in each experiment, see figure legends and Supplementary Tables 1–12.

### Visual overstimulation

LED strips around the home cage emitted warm white light (~2,200 K) at 40 Hz and a 50% duty cycle from the start of the light cycle for four bouts of 1 h on, alternated with 1 h off, over 8 h. Stimulation covered 3–6 d (Results). Controls received matched handling and sham stimulation. To test acute impact of overstimulation, AAV1-C-FOS-eYFP (Vigene Biosciences, 1.67 × 10^13^ genome copies (GC) per ml) was injected into V1, and after recovery, in vivo 2-P imaging (‘Functional and structural imaging’) measured c-Fos expression after two 1 h bouts of overstimulation (Supplementary Fig. 1a,b).

### Drug administration

For mGluR5 manipulations (Fig. 1h,i), young adult (P60–100) *Thy1*-eGFP mice were intraperitonially (i.p.) injected at the start of the light cycle with MTEP hydrochloride (10 mg per kg body weight; 2.5 mg ml^−1^ in PBS containing 5% Tween-80; Bio-Techne, 2921/10) or vehicle (PBS containing 5% Tween-80) for 2 d before starting overstimulation, and daily for 3 d before each overstimulation session (see ‘Behavioral experiments’). To investigate PAM of mGluR5, C57BL/6J mice were i.p. injected with VU0409551 (Tocris, 5693; 3 mg per kg body weight; 1 mg ml^−1^ in 20% β-cyclodextrin; Sigma-Aldrich, C4767) in saline. The NMDAR antagonist CPP^31–33^ was administered 24 h before and 1 h before the start of overstimulation in young adult animals (Supplementary Fig. 1d,e). C57BL/6J mice were i.p. injected with CPP (Tocris, 0173; 15 mg per kg body weight; 2.5 mg ml^−1^) in sterilized saline. Drug dosing and timing of delivery followed published reports^22,23,25,47,48^.

### Surgery

Cranial windows were implanted over the right hemisphere of the visual cortex^3,42^. Mice were anesthetized using ketamine and xylazine (i.p. 0.1 mg per gram body weight and 0.01 mg per gram body weight, respectively), followed by subcutaneous injection of carprofen (5 mg per kg body weight) and dexamethasone (1.26 mg per kg body weight). A craniotomy was performed, the skull was replaced with a coverslip and a head bar was attached with dental cement. Where described, mice were injected with: AAV1-C-FOS-eYFP (Vigene Biosciences, 1.67 × 10^13^ GC per ml), AAV2/1-EF1a-mCherry-GSG-P2A-HIS-GCaMP6s-WPRE (Vector Biolabs, 7.0 × 10^12^ GC per ml) and/or pAV-mDlx-Kz-f-cyRFP1-GSG-P2A-GCaMP6S-WPRE-pA (Vigene Biosciences, 8.97 × 10^12^ GC per ml) into superficial V1. Injections were as follows: for cellular imaging, 100 nl of virus at 15 nl min^−1^; for spine imaging, 50–70 nl of virus at 5 nl min^−1^; and for C-FOS-eYFP experiments, 200–300 nl at 15–20 nl min^−1^. Mice recovered for >28 d before imaging.

### Functional and structural imaging

Calcium imaging used a custom 2-P microscope (INSS) with a resonance scanner (Cambridge Technology) and a high-power objective Z-piezo stage (Physik Instrumente), using a Chameleon Vison II laser (Coherent) and a water immersion ×16, 0.8 NA, 3.0 mm WD objective (Nikon). Data were acquired with an 800 MHz digitizer (National Instruments) and pre-processed with a custom-programmed field programmable gate array (National Instruments). Mesoscopic imaging localized the visual cortex^2^. Average laser power was <50 mW and acquisition used ScanImage (Vidrio Technologies). Acquisition parameters for excitatory neurons were: 512 × 512 pixels (340 × 340 µm; Figs. 1 and 2 and Supplementary Figs. 1 and 2), spanning six sections with a 40-µm *z*-step at 5–7 Hz (Figs. 1 and 2). Spine imaging involved a single time series across 512 × 512 pixels (130 × 130 µm; Fig. 4) at 30 Hz, as did excitatory and inhibitory cell recordings (Fig. 5 and Supplementary Fig. 5). The excitation wavelength was set to 920 nm to excite GCaMP6s. At an excitation wavelength of 920 nm, cyRFP (only expressed in inhibitory neurons) is also excited. Emitted light from cyRFP was filtered by an ET605/70M band-pass filter. After functional imaging, the excitation wavelength was set to 1,080 nm to excite mCherry in excitatory neurons^41^. For imaging of C-FOS-eYFP, excitation wavelength was 920 nm (Supplementary Fig. 1a,b). For the in vivo imaging, mice were briefly anesthetized with oxygen and isoflurane (1–1.5%) mix and head-fixed. For all 2-P calcium imaging experiments, mice were anesthetized (0.5% isoflurane) and anesthetic level was the same for all age groups. Mice habituated to the setup before imaging sessions.

A coarse timeline determined time points of interest (Figs. 1c and 2d–f). A more fine-scale time course then investigated within sessions and across days (Fig. 2g–k and Supplementary Fig. 2). For the coarse timeline, data were acquired during a baseline before overstimulation (day 0) and after each day of overstimulation or control: 24 h (day 1), 48 h (day 2) and 72 h (day 3; Figs. 1a–c and 2a–f). For the fine-scale time-course analysis, data were collected during baseline (day 0, S1–4) and after each 1 h overstimulation session (four times per day for 3 d), so that S4 (Fig. 2g–k and Supplementary Fig. 2f–o) was equivalent to the coarse imaging (Figs. 1c and 2f). Data were also collected across S1–4 on day 6 (Fig. 2g–k and Supplementary Fig. 2f–o). In a subset of 12-month-old mice, data were collected 3 d after overstimulation (Supplementary Fig. 2p,q). For spine imaging, data were acquired during baseline (Fig. 4), and the next day, 2 h after the 8-h-long overstimulation protocol (Fig. 4). For functional assemblies (Fig. 5), mice were imaged at baseline (Fig. 5), and then after 2 d of overstimulation (Fig. 5). Recordings consisted of 6–12 trials including three to six trials of 85 s of LED stimulation at 40 Hz for 3 s on, interspersed by 2 s off. This was alternated with three to six 85 s of recording in the dark. Measures of orientation and direction selectivity indices (OSI and DSI) were collected and analyzed in response to randomly presented drifting-grating stimuli^2,43^.

### Calcium imaging analysis

For cellular and spine analysis, full-frame data were registered (Moco, ImageJ v1.53t) (ref. ^77^) and analyzed by at least three experimenters who were blind to condition and time course. Cellular (and background) regions of interest (ROIs) were drawn. Cells with filled nuclei due to GCaMP overexpression were excluded. Signal processing removed slow changes in fluorescence, normalized to the background and thresholded (15%). For excitatory and inhibitory neurons, activity was estimated as the AUC of the *ΔF/F*_0_ trace per second^2,42,43,78^. Measures of *ΔF/F*_0_ amplitude and frequency were made using peak detection in MATLAB^3^. Total cellular activity was taken as activity recorded during LED stimulation and in the dark. In a subset of analyses, activity was normalized to baseline levels, by either subtracting the AUC of each cell’s activity from its baseline (Figs.1c and 2f), or by normalizing the AUC of active cells and spines to the mean of the baseline (Figs. 2g,h, 4 and 5 and Supplementary Fig. 2). Deconvolution of calcium traces used standard approaches^79^. Analysis estimating highly active neurons (Supplementary Fig. 2c) was adapted from published approaches^8^. Calcium transient frequency was calculated and the percentage of neurons falling into the low (minimum–Q1), middle (Q2–Q3) and high (>Q3) range based on the 3-month-old control distribution was counted.

Spine signal extraction was performed as described^41,42,80^. After registration via ‘Moco’^77^ and manual translation for drift or rotation, stacks were visually inspected for *z*-axis movement and discarded if a stable dendrite was not evident. Dendritic sections were excluded if there was evidence of crossing axons or dendrites. Calcium responses from individual spines were isolated from global dendritic signals using a subtraction procedure^41,42,80^. Circular ROIs were drawn over spines during a period of resting state and visual stimulation to compute *ΔF/F*_0_spine_. Circular ROIs of the same size were drawn around the adjacent parent dendrite to calculate the local dendritic signal, *ΔF/F*_0_dendrite_. The dendrite-related component was removed from the spine signals by subtracting a scaled version of the dendritic signal (Fig. 4a–c and Supplementary Fig. 4a–c). Major results were then confirmed by additional analysis, subtracting the dendrite from spine signal.

Spine events were analyzed using peak detection (15% *ΔF/F*_0_ threshold), the AUC and frequency. To estimate visual responsivity, visually evoked spine responses were calculated as the fraction of times a 40-Hz light flicker was presented (48 events) and evoked a time-locked response (Fig. 4h). Spine activity was then collected in the dark and aligned to a dummy visual stimulus to give a false positive rate. The 80th percentile of the false positive rate was taken as a threshold and spines with more time-locked responses than this cutoff were considered visually responsive to the presented stimulus (Fig. 4h and Supplementary Fig. 4f) with others labeled as nonvisual (Fig. 4h and Supplementary Fig. 4f).

Spine activity before and after overstimulation was compared by normalizing the AUC to the baseline mean. For spatial clustering analysis, we calculated the probability of a spine ‘*n*’ positions away from a ${\mathrm{{spine}}}_{0}$ matching in functional property^3^. A match was given a value of 1 and a non-match a value of 0. We repeated this measure for each spine on a branch serving as ${\mathrm{{spine}}}_{0}$ (Supplementary Fig. 4g,h) and compared a shuffled distribution to the experimental distribution using Mann–Whitney rank-sum *t*-test.

Functional assembly dynamics were estimated using positive and significant pairwise correlations between thresholded calcium signals^2,43,44^. For each neuron, we calculated the average correlation strength with other neurons in the same region^2^. Values were calculated at baseline and after overstimulation (Post-stim; Fig. 5) and normalized to baseline.

### Electrophysiology

Whole-cell patch-clamp recordings were based on previous methods^2,3^. Anesthetized mice were transcardially perfused with 10 ml of 4 °C oxygenated artificial cerebrospinal fluid (ACSF; 108 mM choline-Cl, 3 mM KCl, 26 mM NaHCO_3_, 1.25 mM NaHPO_4_, 25 mM d-glucose, 3 mM Na pyruvate, 2 mM CaCl_2_ and 1 mM MgSO_4_ saturated with 95% O_2_/5% CO_2_). Coronal brain slices (300 µm thick) were prepared (Campden Instruments) containing visual cortex (Allen mouse brain atlas; https://portal.brain-map.org/) and incubated in a holding chamber before recordings at room temperature (RT) in ACSF (126 mM NaCl, 3.5 mM KCl, 25 mM NaHCO_3_, 1 mM NaHPO_4_, 25 mM d-glucose, 2 mM CaCl_2_ and 1 mM MgSO_4_ saturated with 95% O_2_/5% CO_2_). L2/3 pyramidal neurons were identified by spiking and soma shape (SlicePro3000, Scientifica). Recordings were made in current clamp (Multiclamp 700B, Molecular Devices), using acquisition software (Wavesurfer v2.1.0, Janelia). Patch pipettes (4–7 MΩ) contained the following: 130 mM KMeSO_4_, 8 mM NaCl, 2 mM KH_2_PO_4_, 2 mM d-glucose and 10 mM HEPES. mEPSP/mIPSP recordings were made at –70 mV or +10 mV respectively with 1 µM tetrodotoxin. Recordings were discarded if series resistance varied (±15%) or resting membrane potential or input resistance varied (±10%). mEPSP/mIPSP recordings were filtered at 3 kHz, digitized at 20 kHz and analyzed blind to experimental condition using MiniAnalysis (Synaptosoft v6.0.7) (ref. ^2^), with a threshold of 2.5 times the root mean square of the noise. The variables extracted here were the mEPSP/mIPSP amplitude and frequency, and mIPSP inter-event interval. Spontaneous EPSP/IPSP recordings were made at −70 mV or −55 mV^81^, and analyzed using similar approaches. Neuronal excitability was assessed via somatic injection of a 500 ms depolarizing current step with 10 pA increments until an action potential was elicited. Passive membrane properties were estimated from −100 pA current injections, based on membrane deflection and decay time course. The synaptic E:I ratio was calculated as: ${\mathrm{E}}:{\mathrm{I}}\,{\mathrm{{ratio}}}=({mEPSP}_{{amp}}\times {{mEPSP}}_{{freq}})/({mIPSP}_{{amp}}\times {{mIPSP}}_{{freq}})$ (Figs. 1d and 3a).

### Immunofluorescence

Animals were transcardially perfused with 10 ml of ice-cold (4 °C) oxygenated dissection ACSF (‘Electrophysiology’). Brains were post-fixed (4% paraformaldehyde) at 4 °C overnight and washed in 1× PBS and transferred to 30% sucrose and 0.1% NaN_3_ for 2 d. Coronal sections were made using a freezing microtome at a thickness of 80 µm, washed in 1× TBS, incubated for 1 h in blocking solution (10% normal horse serum; Jackson ImmunoResearch, 000008-000-121), 1% Triton X-100 in 1× TBS) at RT, and then in primary antibody at 4 °C overnight. Sections were washed in 1× TBS-0.25% Triton X-100 at RT and incubated for 2 h in secondary antibody in blocking solution at RT. Sections were mounted (Vectashield Non-Hardening Mounting Media, Vectorlabs, H1000) with coverslips. For antibody details, see Supplementary Table 13. Sections were imaged with a confocal microscope (SP8 Lightning, Leica, Leica Application Suite X, v3.5.7) using Diode 405 and 638, and OPSL 488 and 552 lasers. For quantification, images were taken with a HC PL APO CS2 ×20/0.75 dry objective (Leica), using a 2% digital zoom (290 µm^2^ per tile), and a *z*-step of 1 µm. For spine and synaptic protein analysis, images were taken with an HC PL APO CS2 ×40/1.30 oil objective (Leica), using a 5% digital zoom (58 µm^2^ per tile) and *z*-step of 0.5 µm.

### c-Fos and synaptic measures

Brain sections from *Thy1*-eGFP animals or animals injected with viral C-FOS-eYFP (‘Surgery’; Fig. 3d–h) underwent immunofluorescence protocols for VGAT, GluA2, GRIP1, HOMER1 and eEF2K (‘Immunofluorescence’). Spine size and density were measured at eGFP/c-Fos-YFP-positive dendritic branches by drawing fluorescence intensity profiles along dendrites (ImageJ^3^) and then normalizing profiles to the background fluorescence. Peak (>15% of background) width was taken as a proxy for spine size and peak number divided by length for density (Figs. 1 and 3 and Supplementary Figs. 1 and 3). A similar approach measured synaptic proteins at eGFP (Fig. 1 and Supplementary Fig. 1), c-Fos-YFP-positive (Fig. 3 and Supplementary Fig. 3) and GAD-positive (Supplementary Fig. 5) neurons. To identify colocalization, we took peaks of synaptic protein expression overlapping with spine peaks and calculated the ratio of the peak protein intensity over the spine width. To test for multiplicative scaling values^3,22,35,69^ (Supplementary Fig. S1o–x), an initial scaling factor value was generated using the median values from control and post-overstimulation distributions. We then tested scaling values around this seed point using the Kolmogorov–Smirnov distance metric between the scaled control distribution and the synaptic measures from overstimulated animals. To quantify c-Fos expression for structural synaptic E:I ratio analysis (Fig. 3h), fluorescence intensity profiles from dendrites were normalized to background and the AUC was calculated. The structural synaptic E:I ratio was calculated as: $\mathrm{{Structural}\,E:I\,{ratio}={{Spine}}_{{integral}}/{{VGAT}}_{{integral}}}$ (Fig. 3h).

Immunofluorescence labeling of c-Fos on sections labeled with C-FOS-eYFP construct tested if the viral activity tagging strategy reported similar c-Fos expression to that obtained using staining (Supplementary Fig. 3i). For quantification of c-Fos expression, we took the percentage of c-Fos-positive neurons 20% greater than background. In PFC sections, we calculated c-Fos percentage values using a bootstrap approach and sampled with replacement 500 times in batches of 10 neurons. We also compared VGAT density at dendrites from neurons with weak c-Fos expression to VGAT density at dendrites with greater c-Fos levels (Supplementary Fig. 3n–q). We normalized c-Fos values from control animals to the mean of each distribution within age group and pooled across ages (Supplementary Fig. 3n). We considered c-Fos levels less than or equal to the 30th percentile of the control distribution to be weakly expressing c-Fos (Supplementary Fig. 3n) and compared VGAT density values at these dendrites with greater levels (>30th percentile) of c-Fos expression (Supplementary Fig. 3o–q).

### Computational modeling

We simulated a two-compartment somatic and dendritic model (Supplementary Fig. 4j). Neuronal activity depends on somatic input (${I}_{\mathrm{{soma}}}$) and excitatory and inhibitory dendritic inputs (${I}_{\mathrm{{dend}}}$) according to equation (1):

1 $$\tau \,{dr}/{dt}=-r+[{I}_{\mathrm{soma}}+[I_{\mathrm{dend}}]_{+}]_{+}$$

Here, $\tau$ is the time constant of network integration $(\tau =10)$, and ${[]}_{+}$ denotes rectification. Each input component is given, respectively, as per equation (2):

2 $$\begin{array}{c}{I}_{\mathrm{{soma}}}={I}_{{ffw}}+{r}_{\mathrm{{exc}}}-{r}_{\mathrm{{inh}}}\\ {I}_{\mathrm{{dend}}}=\frac{1}{N}\mathop{\sum }\limits_{i=1}^{N}{w}_{i}{r}_{i}-{I}_{\mathrm{{inh}}}\end{array}$$

${I}_{{ffw}}$ is the feedforward input to the neuron ${(I}_{{ffw}}=0.5)$ and ${r}_{\mathrm{{exc}}}$ and ${r}_{\mathrm{{inh}}}$ are the aggregate rate of excitatory and inhibitory inputs impinging on the somatic compartment, which are kept in balance (${r}_{\mathrm{{exc}}}={r}_{\mathrm{{inh}}}$). $N$ is the number of excitatory inputs from presynaptic sources, while ${r}_{i}$ and ${w}_{i}$ denote the activity and the connection weight of the *i*-th source, respectively. Total inhibitory input at this component, ${I}_{\mathrm{{inh}}}$, is modeled as a single aggregate input, summarizing the combined effect of the activity and weight of presynaptic inhibitory sources. Overstimulation changes the activity of the network, due to primarily feedforward mechanisms that depend on the visual responsiveness of the neuron. The initial firing rate of the *i*-th neuron in the network is given by ${r}_{i}={r}_{0}+{r}_{m}$, where ${r}_{0}$ is the baseline firing rate before overstimulation (${r}_{0}=1$), and ${r}_{m}$ is the modulation of activity by visual stimulation. ${r}_{m}$ alternates between on and off states, with the level of modulation depending on the visual responsiveness of the neuron ($v$): ${r}_{m}={v}$ for ${T}_{\mathrm{on}}$ and ${r}_{m}=0$ for ${T}_{\mathrm{{off}}}$. ${T}_{\mathrm{{on}}}={T}_{\mathrm{{off}}}=100{\mathrm{ms}}$. Visual responsiveness for each neuron, ${v}_{i}$, is drawn randomly from a uniform distribution between 0 (nonvisual) and 1 (the most visual). The postsynaptic neuron receives input from presynaptic neurons with levels of visual responsiveness. The initial weight of connections is set as: ${w}_{i}=1+\xi$, where $\xi$ is an independent and identically distributed random variable drawn from a normal distribution with $N(\mathrm{0,0.1})$.

Evolution of the activity of the neuron in equation (1) depends on the plasticity of its weights ($w$ in equation (2)). The weight of the dendritic input from the *i*-th presynaptic excitatory neuron, ${w}_{i}$, is evolving, in turn, according to equation (3):

3 $$\frac{d{w}_{i}}{{dt}}={A}_{1}{r}_{i}\,\left({r}_{i}-{r}_{0}\right)r\,\left(1-{I}_{\mathrm{{inh}}}\right)-{A}_{2}{\rm{r}}$$

where $r$ is the postsynaptic activity described in equation (1) and ${r}_{0}$ is the baseline activity. The first plasticity term describes the Hebbian component of learning, which comprises presynaptic rates (${r}_{i}$) and postsynaptic activity (${\rm{r}}$). The strength of this learning is controlled by the parameter ${A}_{1}$, which is set to ${A}_{1}=1\times 1{0}^{-4}$ for both young and old animals and is modulated by inhibition ($1\mbox{--}{I}_{\mathrm{{inh}}}$). In both young and old animals, at a baseline state ${{I}^{\mathrm{{young}}}}_{\mathrm{{inh}}}={{I}^{\mathrm{{old}}}}_{\mathrm{{inh}}}=0.2$. The second term on the right-hand side in equation (3) is a homeostasis term, which downscales the weights as a function of the postsynaptic activity of the neuron, and its strength is controlled by the downscaling parameter ${A}_{2}$. Following the experimental results, we find that in young adult animals, both inhibitory synaptic mechanisms and excitatory synaptic weakening may regulate the network activity after overstimulation (Figs. 2f and 3a–c). Therefore, after overstimulation, inhibition is upregulated in the model to a higher value by allowing ${{I}^{\,\mathrm{{young}}}}_{\mathrm{{inh}}}=0.4$, while the downscaling parameter is set to ${{A}^{\mathrm{young}}}_{2}=0.56\times 1{0}^{-4}$. Experimental findings show that in old animals excitatory synaptic weakening and the increased inhibitory response to sensory overstimulation are reduced (Figs. 3a–c and 4f). This is modeled by a lack of increased inhibition (fixed), with ${{I}^{\mathrm{{old}}}}_{\mathrm{{inh}}}$ remaining at the same baseline value of $0.2$, and a reduction in levels of downscaling in old animals, where ${{A}^{\mathrm{{old}}}}_{2}=0.48\times {10}^{-4}$. Each neuron receives input from $N=200$ presynaptic excitatory sources. The evolution of weights and the postsynaptic response is modeled for $T={10,000}$, and simulations are performed with time steps of ${dt}=1$. The response of excitatory presynaptic sources projecting to dendrites evolves in time according to equation (4):

4 $${{r}_{i}(t)=r}_{0}+{v}_{i}+\alpha \,r(t)+\zeta (t)$$

where ${v}_{i}$ is the visual responsivity of the respective presynaptic neuron, as described above, and $\zeta (t)$ is an independent and identically distributed random variable drawn from $N(\mathrm{0,0.1})$. $\alpha$ is the feedback parameter, which changes the activity of presynaptic neurons as a function of the postsynaptic activity. We chose $\alpha =0.1$. This models how the change in the activity of postsynaptic neurons is reflected in the activity of presynaptic neurons, which are connected in a recurrent network. The activity of the postsynaptic neuron in turn would change as a result. Excitatory and inhibitory inputs to the somatic compartment are modeled as ${r}_{\mathrm{{exc}}}(t)={r}_{0}+\zeta (t)$ and ${r}_{\mathrm{{inh}}}(t)={r}_{0}+\zeta (t)$, where $\zeta (t)$ is drawn from a uniform distribution of $(\mathrm{0,0.1})$.

To test the contribution of plasticity mechanisms (Supplementary Fig. 4m–r), initial conditions matched our late adult simulation (Supplementary Fig. 4m,p). We then progressively reinstated plasticity mechanisms individually and measured the impact on synaptic weights of simulated overstimulation (Supplementary Fig. 4m–r). Specifically, we increased the parameter controlling the strength of dendritic inhibition (${I}_{\mathrm{{inh}}}$) from its late adult value (0.2) to 0.5 (corresponding to strong inhibition in young adult): ${I}_{\mathrm{{inh}}}=\left[0.2,0.3,0.4,0.5\right]$ (Supplementary Fig. 4m–o). We then kept dendritic inhibition fixed at late adult values and modulated the downscaling homeostatic term (${A}_{2}$). The strength of downscaling was increased from its weak value in the late adult to stronger values: $\left[0.6,0.7,0.8,0.9\right]$ (Supplementary Fig. 4p–r). For these simulations, we quantified the normalized change in the plasticity of the synaptic weights following modulation of either inhibition (Supplementary Fig. 4s) or downscaling (Supplementary Fig. 4t) as a function of the late adulthood simulation.

### Behavioral experiments

Behavioral tests used Bussey-Saksida touchscreen operant chambers (Campden Instruments). ABET II (Campden Instruments) and WhiskerServer (Cambridge University Technical Services) software were used to run the boxes. Behavioral testing was conducted as previously described^45^. Three days before testing, animals were food restricted to 85–90% of free feeding weight. This weight was maintained throughout testing and water was available ad libitum^45^. Following habituation to the setup, mice underwent three stages of the rCPT (Fig. 6a and Supplementary Fig. 6a)^45^. Each session lasted 45 min a day, or 100 rewards. Stage 1 and 2 criteria required mice to earn 100 rewards within the 45-min session. For stage 1, mice were trained to touch a white-outlined square on the screen. For stage 2, the white frame was replaced by a target (S+) stimulus (horizontal or vertical line) presented for 5 s. For stage 3, a non-target (S−) stimulus (a ‘snowflake’) was introduced and randomly presented on 50% of the trials.

For stages 2–3, entry into the reward magazine initiated a delay period of 2 s before proceeding to the next intertrial interval (3–5 s). A touch to the non-target (S−) resulted in an intertrial interval period before a correction trial. On correction trials, the stimulus was always S−, and consecutive correction trials continued until no response to S− was made. For stage 3, animals had to touch the screen when S+ was presented (hit) or withhold their response to S− (correct rejection). Failing to respond to S+ (miss) or responding to S− (mistake) were also measured. These metrics were used to calculate HR, $\mathrm{{HR}={hits}/({hits}+{misses})}$, and FAR, $\mathrm{{FAR}={mistakes}/({mistakes}+{correct\; rejections})}$, which in turn calculated $\mathrm{{Performance}={HR}-{FAR}}$. Learning rate was calculated by taking a linear fit to the performance data acquired over sessions and estimating the slope (Supplementary Fig. 6b). The reward latency, correct choice latency and total number of trials were also measured.

To assess overstimulation on cognitive performance, 3-month-old, 8-month-old and 12-month-old animals underwent overstimulation. After a rest period (~1 h), performance on the rCPT was assessed (Fig. 6b,c and Supplementary Fig. 6b). We administered MTEP to young adult mice (3 months old), or the mGluR5 PAM VU0409551 to late adult animals (12 months old) and tested performance on the rCPT. In these experiments, all animals underwent habituation and stages 1–2. Animals then had a 2-d break from the rCPT, during which they received daily i.p. injections of MTEP/vehicle, or the mGluR5 PAM VU0409551. These were administered at the start of their light cycle (‘Drug administration’). Stage 3 of rCPT testing then ran over 7 d. For stage 3, daily i.p. administration of the drugs was given, followed by daily overstimulation or sham (control), and then rCPT testing (Fig. 6e,f). Dosing and time of delivery was based on previously published reports^23,25,47,48^.

### Statistical analysis

No statistical methods were used to predetermine sample sizes, but our sample sizes are similar to those reported in previous publications^2,3,42,82^. Data underwent tests of normality to inform the use of parametric and non-parametric tests. Statistical analyses were performed in MATLAB (version R2019b) or SigmaPlot (version 14.0, Systat Software) using parametric or non-parametric tests, as required: Student’s *t*-test, Mann–Whitney rank-sum *t*-test, Welch’s *t*-test, paired Student’s *t*-test, Wilcoxon signed-rank test, Chi-squared test, one-way ANOVA with Holm–Šidák post hoc test, Kruskal–Wallis one-way ANOVA with Dunn’s test, repeated-measures ANOVA with Holm–Šidák post hoc test, Friedman repeated-measures ANOVA with Tukey’s test or a two-way ANOVA with Holm–Šidák post hoc test. Correlation coefficients were calculated with a Pearson’s correlation coefficient. Specific statistical tests used for all figures along with the number of samples can be found in Supplementary Tables 1–12.

### Reporting summary

Further information on research design is available in the Nature Portfolio Reporting Summary linked to this article.

## Online content

Any methods, additional references, Nature Portfolio reporting summaries, source data, extended data, supplementary information, acknowledgements, peer review information; details of author contributions and competing interests; and statements of data and code availability are available at 10.1038/s41593-023-01451-z.

### Supplementary information

Supplementary Information Supplementary Tables 1–6 and 13, and Figs. 1–6 and legends

Reporting Summary

Supplementary Tables 7–12 Statistics tables for Supplementary Figs. 1–6.

Supplementary Data Source data for supplementary figures.

Supplementary Code Modeling code.

### Source data

Source Data Fig. 1 Raw data and statistical source data.

Source Data Fig. 2 Raw data and statistical source data.

Source Data Fig. 3 Raw data and statistical source data.

Source Data Fig. 4 Raw data and statistical source data.

Source Data Fig. 5 Raw data and statistical source data.

Source Data Fig. 6 Raw data and statistical source data.

## Data Availability

Raw data used in this study and requests for resources and reagents are available from the corresponding author upon reasonable request. Source data are provided with this paper.

## Code availability

The code that supports the computational modeling in this paper is available in the Supplementary Information. Additional relevant code is available from the corresponding author upon reasonable request.

## References

[1] Turrigiano GG (2017). The dialectic of Hebb and homeostasis. Philos. Trans. R. Soc. B Biol. Sci..

[2] Barnes SJ (2015). Subnetwork-specific homeostatic plasticity in mouse visual cortex in vivo. Neuron.

[3] Barnes SJ (2017). Deprivation-induced homeostatic spine scaling in vivo is localized to dendritic branches that have undergone recent spine loss. Neuron.

[4] Bridi MCD (2020). Daily oscillation of the excitation–inhibition balance in visual cortical circuits. Neuron.

[5] Radulescu, C. I., Cerar, V., Haslehurst, P., Kopanitsa, M. & Barnes, S. J. The aging mouse brain: cognition, connectivity and calcium. *Cell Calcium*10.1016/j.ceca.2021.102358 (2021). 10.1016/j.ceca.2021.102358 33517250

[6] Mahoney RE, Rawson JM, Eaton BA (2014). An age-dependent change in the set point of synaptic homeostasis. J. Neurosci..

[7] Nahmani M, Turrigiano GG (2014). Adult cortical plasticity following injury: recapitulation of critical period mechanisms?. Neuroscience.

[8] Lerdkrai C (2018). Intracellular Ca^2+^ stores control in vivo neuronal hyperactivity in a mouse model of Alzheimer’s disease. Proc. Natl Acad. Sci. USA.

[9] Styr, B. & Slutsky, I. Imbalance between firing homeostasis and synaptic plasticity drives early-phase Alzheimer’s disease. *Nat. Neurosci*. 10.1038/s41593-018-0080-x (2018). 10.1038/s41593-018-0080-x PMC6533171 29403035

[10] Haberman RP, Koh MT, Gallagher M (2017). Heightened cortical excitability in aged rodents with memory impairment. Neurobiol. Aging.

[11] Voytek B (2015). Age-related changes in 1/*f* neural electrophysiological noise. J. Neurosci..

[12] Hua T, Kao C, Sun Q, Li X, Zhou Y (2008). Decreased proportion of GABA neurons accompanies age-related degradation of neuronal function in cat striate cortex. Brain Res. Bull..

[13] Fu Y, Yu S, Ma Y, Wang Y, Zhou Y (2013). Functional degradation of the primary visual cortex during early senescence in rhesus monkeys. Cereb. Cortex.

[14] Schmolesky MT, Wang Y, Pu M, Leventhal AG (2000). Degradation of stimulus selectivity of visual cortical cells in senescent rhesus monkeys. Nat. Neurosci..

[15] Kaneko M, Stellwagen D, Malenka RC, Stryker MP (2008). Tumor necrosis factor-alpha mediates one component of competitive, experience-dependent plasticity in developing visual cortex. Neuron.

[16] De Gois S (2005). Homeostatic scaling of vesicular glutamate and GABA transporter expression in rat neocortical circuits. J. Neurosci..

[17] Turrigiano GG, Leslie KR, Desai NS, Rutherford LC, Nelson SB (1998). Activity-dependent scaling of quantal amplitude in neocortical neurons. Nature.

[18] Dörrbaum AR, Alvarez-Castelao B, Nassim-Assir B, Langer JD, Schuman EM (2020). Proteome dynamics during homeostatic scaling in cultured neurons. eLife.

[19] Siddoway B, Hou H, Xia H (2014). Molecular mechanisms of homeostatic synaptic downscaling. Neuropharmacology.

[20] Turrigiano G (2011). Too many cooks? Intrinsic and synaptic homeostatic mechanisms in cortical circuit refinement. Annu. Rev. Neurosci..

[21] Turrigiano GG (2008). The self-tuning neuron: synaptic scaling of excitatory synapses. Cell.

[22] Torrado Pacheco A, Bottorff J, Gao Y, Turrigiano GG (2021). Sleep promotes downward firing rate homeostasis. Neuron.

[23] Chokshi V (2019). Input-specific metaplasticity in the visual cortex requires Homer1a-mediated mGluR5 signaling. Neuron.

[24] de Vivo L (2017). Ultrastructural evidence for synaptic scaling across the wake/sleep cycle. Science.

[25] Diering GH (2017). Homer1a drives homeostatic scaling-down of excitatory synapses during sleep. Science.

[26] Wu C-H, Ramos R, Katz DB, Turrigiano GG (2021). Homeostatic synaptic scaling establishes the specificity of an associative memory. Curr. Biol..

[27] Maffei A, Turrigiano GG (2008). Multiple modes of network homeostasis in visual cortical layer 2/3. J. Neurosci..

[28] Ranson A, Cheetham CEJ, Fox K, Sengpiel F (2012). Homeostatic plasticity mechanisms are required for juvenile, but not adult, ocular dominance plasticity. Proc. Natl Acad. Sci. USA.

[29] Ding Y (2017). Changes in GABAergic markers accompany degradation of neuronal function in the primary visual cortex of senescent rats. Sci. Rep..

[30] Wang H, Xie X, Li X, Chen B, Zhou Y (2006). Functional degradation of visual cortical cells in aged rats. Brain Res..

[31] Villarreal DM, Do V, Haddad E, Derrick BE (2002). NMDA receptor antagonists sustain LTP and spatial memory: active processes mediate LTP decay. Nat. Neurosci..

[32] Sato M, Stryker MP (2008). Distinctive features of adult ocular dominance plasticity. J. Neurosci..

[33] Toyoizumi T, Kaneko M, Stryker MP, Miller KD (2014). Modeling the dynamic interaction of Hebbian and homeostatic plasticity. Neuron.

[34] Feng G (2000). Imaging neuronal subsets in transgenic mice expressing multiple spectral variants of GFP. Neuron.

[35] Kim J, Tsien RW, Alger BE (2012). An improved test for detecting multiplicative homeostatic synaptic scaling. PLoS ONE.

[36] Gainey MA, Tatavarty V, Nahmani M, Lin H, Turrigiano GG (2015). Activity-dependent synaptic GRIP1 accumulation drives synaptic scaling up in response to action potential blockade. Proc. Natl Acad. Sci. USA.

[37] Gainey MA, Hurvitz-Wolff JR, Lambo ME, Turrigiano GG (2009). Synaptic scaling requires the GluR2 subunit of the AMPA receptor. J. Neurosci..

[38] Diering GH, Huganir RL (2018). The AMPA receptor code of synaptic plasticity. Neuron.

[39] Park S (2008). Elongation factor 2 and fragile X mental retardation protein control the dynamic translation of Arc/Arg3.1 essential for mGluR-LTD. Neuron.

[40] Barth AL, Gerkin RC, Dean KL (2004). Alteration of neuronal firing properties after in vivo experience in a FosGFP transgenic mouse. J. Neurosci..

[41] Chen T-W (2013). Ultrasensitive fluorescent proteins for imaging neuronal activity. Nature.

[42] Doostdar, N. et al. Multi-scale network imaging in a mouse model of amyloidosis. *Cell Calcium*10.1016/j.ceca.2021.102365 (2021). 10.1016/j.ceca.2021.102365 33610083

[43] Knöpfel T (2019). Audio-visual experience strengthens multisensory assemblies in adult mouse visual cortex. Nat. Commun..

[44] Wilson DE (2017). GABAergic neurons in ferret visual cortex participate in functionally specific networks. Neuron.

[45] Kim CH (2015). The continuous performance test (rCPT) for mice: a novel operant touchscreen test of attentional function. Psychopharmacol..

[46] Lüscher C, Huber KM (2010). Group 1 mGluR-dependent synaptic long-term depression: mechanisms and implications for circuitry and disease. Neuron.

[47] Doria JG (2018). The mGluR5-positive allosteric modulator VU0409551 improves synaptic plasticity and memory of a mouse model of Huntington’s disease. J. Neurochem..

[48] Rook JM (2015). Biased mGlu5 positive allosteric modulators provide in vivo efficacy without potentiating mGlu5 modulation of NMDAR currents. Neuron.

[49] Lehmann K, Schmidt K-F, Löwel S (2012). Vision and visual plasticity in ageing mice. Restor. Neurol. Neurosci..

[50] Vossel KA (2016). Incidence and impact of subclinical epileptiform activity in Alzheimer’s disease. Ann. Neurol..

[51] Aziz W (2019). Multi-input synapses, but not LTP-strengthened synapses, correlate with hippocampal memory storage in aged mice. Curr. Biol..

[52] Bienenstock EL, Cooper LN, Munro PW (1982). Theory for the development of neuron selectivity: orientation specificity and binocular interaction in visual cortex. J. Neurosci..

[53] Bridi MCD (2018). Two distinct mechanisms for experience-dependent homeostasis. Nat. Neurosci..

[54] Glazewski S, Greenhill S, Fox K (2017). Time-course and mechanisms of homeostatic plasticity in layers 2/3 and 5 of the barrel cortex. Philos. Trans. R. Soc. Lond. B. Biol. Sci..

[55] Greenhill SD, Ranson A, Fox K (2015). Hebbian and homeostatic plasticity mechanisms in regular spiking and intrinsic bursting cells of cortical layer 5. Neuron.

[56] Mrsic-Flogel TD (2007). Homeostatic regulation of eye-specific responses in visual cortex during ocular dominance plasticity. Neuron.

[57] Teichert M, Liebmann L, Hübner CA, Bolz J (2017). Homeostatic plasticity and synaptic scaling in the adult mouse auditory cortex. Sci. Rep..

[58] Inagaki T (2008). Brain-derived neurotrophic factor-mediated retrograde signaling required for the induction of long-term potentiation at inhibitory synapses of visual cortical pyramidal neurons. Neurosci. Res..

[59] Xue M, Atallah BV, Scanziani M (2014). Equalizing excitation–inhibition ratios across visual cortical neurons. Nature.

[60] Qiu J (2016). Decreased *Npas4* and *Arc* mRNA levels in the hippocampus of aged memory‐impaired wild‐type but not memory preserved 11β‐HSD1 deficient mice. J. Neuroendocrinol..

[61] Spiegel I (2014). Npas4 regulates excitatory–inhibitory balance within neural circuits through cell-type-specific gene programs. Cell.

[62] Shan W (2018). Neuronal PAS domain protein 4 (Npas4) controls neuronal homeostasis in pentylenetetrazole-induced epilepsy through the induction of Homer1a. J. Neurochem..

[63] Kaja S (2013). Homer-1a immediate early gene expression correlates with better cognitive performance in aging. Age.

[64] Horn ME, Nicoll RA (2018). Somatostatin and parvalbumin inhibitory synapses onto hippocampal pyramidal neurons are regulated by distinct mechanisms. Proc. Natl Acad. Sci. USA.

[65] Kumar D, Thakur MK (2015). Age-related expression of Neurexin1 and Neuroligin3 is correlated with presynaptic density in the cerebral cortex and hippocampus of male mice. Age.

[66] Hengen KB, Lambo ME, Van Hooser SD, Katz DB, Turrigiano GG (2013). Firing rate homeostasis in visual cortex of freely behaving rodents. Neuron.

[67] Lambo ME, Turrigiano GG (2013). Synaptic and intrinsic homeostatic mechanisms cooperate to increase L2/3 pyramidal neuron excitability during a late phase of critical period plasticity. J. Neurosci..

[68] Ma Z, Turrigiano GG, Wessel R, Hengen KB (2019). Cortical circuit dynamics are homeostatically tuned to criticality in vivo. Neuron.

[69] Keck T (2013). Synaptic scaling and homeostatic plasticity in the mouse visual cortex in vivo. Neuron.

[70] Keck T (2011). Loss of sensory input causes rapid structural changes of inhibitory neurons in adult mouse visual cortex. Neuron.

[71] Ibata K, Sun Q, Turrigiano GG (2008). Rapid synaptic scaling induced by changes in postsynaptic firing. Neuron.

[72] Knott GW, Quairiaux C, Genoud C, Welker E (2002). Formation of dendritic spines with GABAergic synapses induced by whisker stimulation in adult mice. Neuron.

[73] Burke SN, Barnes CA (2006). Neural plasticity in the ageing brain. Nat. Rev. Neurosci..

[74] Adaikkan C (2019). Gamma entrainment binds higher-order brain regions and offers neuroprotection. Neuron.

[75] Cooke SF, Bear MF (2010). Visual experience induces long-term potentiation in the primary visual cortex. J. Neurosci..

[76] Koh MT, Haberman RP, Foti S, McCown TJ, Gallagher M (2010). Treatment strategies targeting excess hippocampal activity benefit aged rats with cognitive impairment. Neuropsychopharmacology.

[77] Dubbs A, Guevara J, Yuste R (2016). moco: fast motion correction for calcium imaging. Front. Neuroinform.

[78] Sammons RP, Clopath C, Barnes SJ (2018). Size-dependent axonal bouton dynamics following visual deprivation in vivo. Cell Rep..

[79] Friedrich J, Zhou P, Paninski L (2017). Fast online deconvolution of calcium imaging data. PLoS Comput. Biol..

[80] Iacaruso MF, Gasler IT, Hofer SB (2017). Synaptic organization of visual space in primary visual cortex. Nature.

[81] Petrache AL (2019). Aberrant excitatory–inhibitory synaptic mechanisms in entorhinal cortex microcircuits during the pathogenesis of Alzheimer’s disease. Cereb. Cortex.

[82] Barnes SJ, Keller GB, Keck T (2022). Homeostatic regulation through strengthening of neuronal network-correlated synaptic inputs. eLife.

