# Supplementary material for: Age-related dysregulation of homeostatic control in neuronal microcircuits
Source: Nat Neurosci. 2023 Nov 2;26(12):2158–70. doi: 10.1038/s41593-023-01451-z (PMC10689243; doi:10.1038/s41593-023-01451-z)
Supplement: Supplementary file 1 — Supplementary Tables 1–6 and 13, and Figs. 1–6 and legends [file 41593_2023_1451_MOESM1_ESM.pdf]

nature neuroscience

Article https://doi.org/10.1038/s41593-023-01451-z

Age-related dysregulation of homeostatic control in neuronal microcircuits

In the format provided by the authors and unedited

Supplementary information

Table S1. Statistical comparisons for Fig. 1.

Statistical comparisons for Fig. 1			Comparisons	Result	
Panel	Description	Test		p value	n value
1c	Average change (normalized to Day 0) in activity (*ΔF/F₀/s*) for control and stimulated groups	Two-way ANOVA with Holm- Šidák<br><br>Group comparisons	Day 1: Con (-0.02±0.03) vs Stim (-0.21±0.03)	$p < 0.001$	Neurons # Con = 193 Stim = 186
			Day 2: Con (-0.01±0.03) vs Stim (-0.20±0.03)	$p < 0.001$	
			Day 3: Con (-0.02±0.03) vs Stim (-0.17±0.03)	$p < 0.001$	
	Average change in activity (*ΔF/F₀/s*) over time compared to baseline (Day 0)	Session comparisons	Control:<br>Day 0 vs 1 (-0.02±0.03)<br>Day 0 vs 2 (-0.01±0.03)<br>Day 0 vs 3 (-0.02±0.03)	$p = 0.998$<br>$p = 0.996$<br>$p = 0.998$	
			Stim:<br>Day 0 vs 1 (-0.21±0.03)<br>Day 0 vs 2 (-0.20±0.03)<br>Day 0 vs 3 (-0.17±0.03)	$p < 0.001$<br>$p < 0.001$<br>$p < 0.001$	
1d	E:I ratio (the integral of mEPSP to mIPSP) for control vs stimulated groups	Welch's t-test	Con (1.04±0.16) vs Stim (0.62±0.08)	$p = 0.023$	Neurons # Con = 16 Stim = 12
1e main	mIPSP inter-event interval (s) for stimulated vs control groups	Mann-Whitney rank sum test	Con (0.34; 0.13-0.84) vs Stim (0.25; 0.09-0.59)	$p < 0.001$	
1e inset			Con (0.66±0.03) vs Stim (0.46±0.02)		
1f main	mEPSP amplitude (mV) for stimulated vs control groups	Mann-Whitney rank sum test	Con (0.24; 0.19-0.31) vs Stim (0.22; 0.18-0.28)	$p < 0.001$	
1f inset			Con (0.26±0.01) vs Stim (0.24±0.01)		
1h main	Normalized (to control) dendritic spine size in *Thy1*-eGFP mice that have undergone intervention (average per dendritic branch)	Kruskal-Wallis (KW) One-Way ANOVA with Dunn's test	Vehicle + Con (1.00±0.03) vs Vehicle + Stim (0.91±0.05)	$p = 0.018$	Dendrites # Veh + Con = 103 Veh + Stim = 62 MTEP + Stim = 25
1h inset			Vehicle + Con vs MTEP + Stim (1.05±0.07)	$p = 0.911$	
1i main	Normalized (to control) VGAT density in *Thy1*-eGFP mice that have undergone intervention. (density per dendritic branch)	KW One-Way ANOVA with Dunn's test	Vehicle + Con (1.00±0.03) vs Vehicle + Stim (1.12±0.04)	$p = 0.036$	Dendrites # Veh + Con = 102 Veh + Stim = 100 MTEP + Stim = 91
1i inset			Vehicle + Con vs MTEP + Stim (1.13±0.04)	$p = 0.043$	

The data for Fig. 1 were obtained from 7 animals for panel c, 19 animals for panels d-f and 9 animals for panels h-i. Data in panel c were normalized to Day 0. Data in panels h-i were normalized to the non-stimulated control group. Two-sided tests were used throughout. Data are presented as mean ± standard error of the mean (S.E.M), or as median and interquartile range (IQR).

Table S2. Statistical comparisons for Fig. 2.

Statistical comparisons for Fig. 2			Comparisons	Result	
**Panel**	**Description**	**Test**		**p value**	**n value**
2d	Percentage (%) of neurons showing reduced activity compared to baseline (BL, Day 0) in the stimulated groups	Chi-square	Average over 3 days:<br>3m (75±2 %) vs 8m (66±5 %)	p = 0.384	Neurons #<br><br>Stim<br>3m = 186<br>8m = 496<br>12m = 445<br><br>Con<br>3m = 193<br>8m = 717<br>12m = 195
			8m vs 12m (44±2 %)	p < 0.001	
			3m vs 12m	p < 0.001	
2e	Percentage (%) of neurons showing reduced activity compared to BL (Day 0) in the control groups	Chi-square	Average over 3 days:<br>3m (51±3 %) vs 8m (44±1 %)	p = 0.338	
			8m vs 12m (40±3 %)	p = 0.592	
			3m vs 12m	p = 0.227	
2f	Average change (normalized to Day 0) in activity ($\Delta F/F_0/s$) for each stimulated age group vs pooled control groups	Two-Way ANOVA with Holm-Šidák test<br><br>Group comparisons	Day 1: Con (0.07±0.02) vs<br>3m (-0.21±0.04)<br>8m (-0.22±0.03)<br>12m (0.06±0.04)	p < 0.001<br>p < 0.001<br>p = 0.791	
			Day 2: Con (0.01±0.02) vs<br>3m (-0.20±0.04)<br>8m (-0.15±0.03)<br>12m (0.09±0.03)	p < 0.001<br>p < 0.001<br>p = 0.015	
			Day 3: Con (0.05±0.02) vs<br>3m (-0.17±0.04)<br>8m (-0.05±0.03)<br>12m (0.12±0.03)	p < 0.001<br>p = 0.005<br>p = 0.029	
	Average change (normalized to Day 0) in activity ($\Delta F/F_0/s$) over time compared to BL (Day 0) in stimulated groups	Session comparisons	3m: Day 0 vs<br>Day 1 (-0.21±0.04)<br>Day 2 (-0.20±0.04)<br>Day 3 (-0.17±0.04)	p = 0.004<br>p = 0.004<br>p = 0.008	
			8m: Day 0 vs<br>Day 1 (-0.22±0.03)<br>Day 2 (-0.15±0.03)<br>Day 3 (-0.05±0.03)	p < 0.001<br>p < 0.001<br>p = 0.163	
			12m: Day 0 vs<br>Day 1 (0.06±0.04)<br>Day 2 (0.09±0.03)<br>Day 3 (0.12±0.03)	p = 0.148<br>p = 0.048<br>p = 0.010	
2g	Norm AUC for total activity in 3 m controls and stimulated animals<br><br>Change (normalized to average BL) – comparison between stimulated and control for each session	Two-Way ANOVA with Holm-Šidák test	BL (0)<br>S1: Stim (1.04±0.13) vs<br>Con (0.98±0.13)<br>S2: Stim (0.97±0.10) vs<br>Con (0.96±0.14)<br>S3: Stim (0.98±0.09) vs<br>Con (1.07±0.14)<br>S4: Stim (1.03±0.13) vs<br>Con (0.99±0.09)	p = 0.942	Neurons #<br><br>Stim = 73<br><br>Con = 136
			Day1<br>S1: Stim (0.86±0.17) vs<br>Con (1.08±0.15)<br>S2: Stim (1.24±0.21) vs<br>Con (1.07±0.14)<br>S3: Stim (1.87±0.18) vs<br>Con (1.05±0.15)<br>S4: Stim (0.49±0.07) vs<br>Con (1.03±0.10)	p = 0.317<br><br>p = 0.432<br><br>p < 0.001<br><br>p = 0.002	
			Day2<br>S1: Stim (0.53±0.08) vs<br>Con (1.21±0.17)<br>S2: Stim (0.76±0.17) vs<br>Con (1.02±0.11)<br>S3: Stim (0.73±0.11) vs<br>Con (0.95±0.12)<br>S4: Stim (0.50±0.07) vs<br>Con (0.94±0.10)	p < 0.001<br><br>p = 0.196<br><br>p = 0.190<br><br>p = 0.004	

			Day3<br>S1: Stim (0.74±0.08) vs Con (1.06±0.11)<br>S2: Stim (0.66±0.13) vs Con (1.14±0.10)<br>S3: Stim (0.55±0.08 vs Con (1.02±0.11)<br>S4: Stim (0.57±0.09) vs Con (1.11±0.09)	$p = 0.062$<br>$p = 0.013$<br>$p = 0.006$<br>$p < 0.001$	
			Day6<br>S1: Stim (0.88±0.13) vs Con (1.06±0.15)<br>S2: Stim (0.70±0.13) vs Con (0.72±0.12)<br>S3: Stim (0.91±0.08) vs Con (0.90±0.11)<br>S4: Stim (0.81±0.17) vs Con (1.01±0.14)	$p = 0.778$	
2h	Norm AUC for total activity in 12 m controls and stimulated animals<br><br>Change (normalized to average BL) – comparison between stimulated and control for each session	Two-Way ANOVA with Holm-Šidák test	BL (0)<br>S1: Stim (1.19±0.16) vs Con (0.98±0.06)<br>S2: Stim (0.96±0.15) vs Con (0.99±0.12)<br>S3: Stim (1.12±0.15) vs Con (0.86±0.13)<br>S4: Stim (0.90±0.07) vs Con (1.09±0.08)	$p = 0.126$	Neurons #<br><br>Stim = 142<br><br>Con = 150
			Day1<br>S1: Stim (1.35±0.16) vs Con (1.11±0.19)<br>S2: Stim (2.18±0.32) vs Con (1.20±0.19)<br>S3: Stim (2.71±0.27) vs Con (1.84±0.17)<br>S4: Stim (1.99±0.22) vs Con (0.99±0.15)	$p = 0.560$<br>$p = 0.023$<br>$p < 0.001$<br>$p = 0.005$	
			Day2<br>S1: Stim (2.07±0.29) vs Con (1.20±0.15)<br>S2: Stim (2.69±0.28) vs Con (1.08±0.16)<br>S3: Stim (2.20±0.32) vs Con (0.85±0.14)<br>S4: Stim (1.87±0.19) vs Con (1.20±0.18)	$p = 0.026$<br>$p < 0.001$<br>$p < 0.001$<br>$p = 0.081$	
			Day3<br>S1: Stim (1.74±0.25) vs Con (1.05±0.11)<br>S2: Stim (2.23±0.26) vs Con (1.37±0.10)<br>S3: Stim (1.81±0.24) vs Con (0.97±0.11)<br>S4: Stim (2.32±0.21) vs Con (0.74±0.09)	$p = 0.091$<br>$p = 0.021$<br>$p = 0.041$<br>$p < 0.001$	
			Day6<br>S1: Stim (2.15±0.25) vs Con (0.98±0.15)<br>S2: Stim (2.32±0.34) vs Con (1.20±0.16)<br>S3: Stim (2.14±0.27) vs Con (0.99±0.16)<br>S4: Stim (2.47±0.38) vs Con (1.24±0.14)	$p = 0.018$<br>$p = 0.033$<br>$p = 0.016$<br>$p = 0.003$	
2i	Average AUC for total activity in 3 m and 12 m stimulated animals	One-Way ANOVA	3m<br>BL<br>S1 (1.39±0.18)<br>S2 (1.32±0.14)<br>S3 (1.31±0.12)<br>S4 (1.38±0.17)	$p = 0.974$	Neurons #<br><br>3m = 73<br>12m = 142

	Calcium-mediated neuronal activity ($\Delta F/F_0/s$) – comparison of each session with the average BL (S1-4) within each age group	One-Way ANOVA with Holm-Šidák	3m<br>BL S1-4 (1.34±0.07)<br>Vs<br>Day 1<br>S1 (1.15±0.23)<br>S2 (1.67±0.28)<br>S3 (2.51±0.24)<br>S4 (0.67±0.09)<br>Day 2<br>S1 (0.72±0.11)<br>S2 (1.02±0.22)<br>S3 (0.98±0.15)<br>S4 (0.66±0.10)<br>Day 3<br>S1 (1.05±0.11)<br>S2 (0.94±0.17)<br>S3 (0.74±0.11)<br>S4 (0.77±0.13)<br>Day 6<br>S1 (1.18±0.17)<br>S2 (0.94±0.18)<br>S3 (1.22±0.11)<br>S4 (1.08±0.22)	 <br><br><br>p = 0.375<br>p = 0.187<br>p < 0.001<br>p < 0.001<br><br>p < 0.001<br>p = 0.097<br>p = 0.039<br>p < 0.001<br><br>p = 0.036<br>p = 0.033<br>p < 0.001<br>p < 0.001<br><br>p = 0.196	
		One-Way ANOVA	12m<br>BL<br>S1 (1.13±0.15)<br>S2 (0.91±0.14)<br>S3 (1.06±0.14)<br>S4 (0.85±0.07)	 p = 0.288	
		One-Way ANOVA with Holm-Šidák	12m<br>BL S1-4 (0.95±0.06)<br>vs<br>Day 1<br>S1 (1.28±0.15)<br>S2 (2.02±0.29)<br>S3 (2.57±0.26)<br>S4 (1.79±0.21)<br>Day 2<br>S1 (1.96±0.28)<br>S2 (2.55±0.27)<br>S3 (2.08±0.30)<br>S4 (1.77±0.18)<br>Day 3<br>S1 (1.65±0.24)<br>S2 (2.12±0.25)<br>S3 (1.72±0.23)<br>S4 (2.20±0.20)<br>Day 6<br>S1 (2.04±0.24)<br>S2 (2.20±0.32)<br>S3 (2.03±0.26)<br>S4 (2.34±0.36)	 <br><br><br>p = 0.221<br>p < 0.001<br>p < 0.001<br>p < 0.001<br><br>p = 0.002<br>p < 0.001<br>p < 0.001<br>p = 0.001<br><br>p = 0.010<br>p < 0.001<br>p = 0.006<br>p < 0.001<br><br>p < 0.001<br>p < 0.001<br>p = 0.001<br>p < 0.001	
	Calcium-mediated neuronal activity ($\Delta F/F_0/s$) comparison between 3m and 12m	Two-Way ANOVA with Holm-Šidák test	BL (0)<br>S1: 3m (1.39±0.18) vs 12m (1.13±0.15)<br><br>S2: 3m (1.32±0.14) vs 12m (0.91±0.14)<br><br>S3: 3m (1.31±0.12) vs 12m (1.06±0.14)<br><br>S4: 3m (1.38±0.17) vs 12m (0.85±0.07)	p = 0.690	

			Day 1<br>S1: 3m (1.15±0.23) vs 12m (1.28±0.15)<br><br>S2: 3m (1.67±0.28) vs 12m (2.07±0.29)<br><br>S3: 3m (2.51±0.24) vs 12m (2.57±0.26)<br><br>S4: 3m (0.67±0.09) vs 12m (1.88±0.21)	p = 0.751<br><br>p = 0.362<br><br>p = 0.888<br><br>p = 0.001	
			Day 2<br>S1: 3m (0.72±0.11) vs 12m (1.96±0.28)<br><br>S2: 3m (1.02±0.22) vs 12m (2.55±0.27)<br><br>S3: 3m (0.98±0.15) vs 12m (2.08±0.30)<br><br>S4: 3m (0.66±0.10) vs 12m (1.77±0.18)	p = 0.030<br><br>p = 0.013<br><br>p = 0.006<br><br>p = 0.004	
			Day 3<br>S1: 3m (1.05±0.12) vs 12m (1.65±0.24)<br><br>S2: 3m (0.94±0.17) vs 12m (2.12±0.25)<br><br>S3: 3m (0.77±0.11) vs 12m (1.72±0.23)<br><br>S4: 3m (0.77±0.13) vs 12m (2.20±0.20)	p = 0.210<br><br>p = 0.017<br><br>p = 0.039<br><br>p < 0.001	
			Day 6<br>S1: 3m (1.18±0.17) vs 12m (2.04±0.24)<br><br>S2: 3m (0.94±0.18) vs 12m (2.20±0.32)<br><br>S3: 3m (1.22±0.11) vs 12m (2.03±0.26)<br><br>S4: 3m (1.08±0.22) vs 12m (2.34±0.36)	p = 0.063<br><br>p < 0.001<br><br>p = 0.011<br><br>p = 0.004	
2j	Average AUC for visual activity in 3 m and 12 m stimulated animals	One-Way ANOVA	3m<br>BL<br>S1 (1.48±0.25)<br>S2 (1.43±0.18)<br>S3 (1.41±0.16)<br>S4 (1.46±0.22)	p = 0.995	

	Calcium-mediated neuronal activity ($\Delta F/F_0/s$) – comparison of each session with the average BL (S1-4) within each age group	One-Way ANOVA with Holm-Šidák	3m<br>BL S1-4 (1.44±0.10)<br>vs<br>Day 1<br>S1 (1.28±0.43)<br>S2 (1.74±0.51)<br>S3 (2.93±0.45)<br>S4 (0.70±0.15)<br>Day 2<br>S1 (0.82±0.16)<br>S2 (0.76±0.26)<br>S3 (0.92±0.21)<br>S4 (0.71±0.16)<br>Day 3<br>S1 (1.23±0.15)<br>S2 (1.11±0.24)<br>S3 (0.84±0.16)<br>S4 (0.94±0.21)<br>Day 6<br>S1 (1.20±0.29)<br>S2 (1.11±0.39)<br>S3 (1.25±0.17)<br>S4 (1.26±0.40)	 <br><br><br>p = 0.660<br>p = 0.561<br>p = 0.001<br>p = 0.017<br><br>p = 0.035<br>p = 0.038<br>p = 0.022<br>p = 0.002<br><br>p = 0.399<br>p = 0.360<br>p = 0.035<br>p = 0.046<br><br>p = 0.741	
		One-Way ANOVA	12m<br>BL<br>S1 (1.13±0.21)<br>S2 (1.11±0.23)<br>S3 (1.04±0.21)<br>S4 (0.95±0.09)	 p = 0.854	
		One-Way ANOVA with Holm-Šidák	12m<br>BL S1-4 (1. 03±0.08)<br>vs<br>Day 1<br>S1 (1.44±0.22)<br>S2 (2.07±0.41)<br>S3 (2.13±0.30)<br>S4 (1.99±0.32)<br>Day 2<br>S1 (2.06±0.44)<br>S2 (3.10±0.41)<br>S3 (2.32±0.51)<br>S4 (2.00±0.32)<br>Day 3<br>S1 (1.93±0.38)<br>S2 (2.58±0.41)<br>S3 (2.16±0.39)<br>S4 (2.33±0.33)<br>Day 6<br>S1 (2.36±0.48)<br>S2 (2.65±0.60)<br>S3 (2.57±0.55)<br>S4 (2.76±0.55)	 <br><br><br>p = 0.242<br>p = 0.008<br>p = 0.005<br>p = 0.001<br><br>p = 0.029<br>p < 0.001<br>p = 0.009<br>p = 0.043<br><br>p = 0.037<br>p < 0.001<br>p = 0.011<br>p = 0.003<br><br>p = 0.024<br>p = 0.010<br>p = 0.022<br>p = 0.002	
	Calcium-mediated neuronal activity ($\Delta F/F_0/s$) – comparison between 3m and 12m	Two-Way ANOVA with Holm-Šidák test	BL (0):<br>S1: 3m (1.48±0.25) vs 12m (1.13±0.21)<br><br>S2: 3m (1.43±0.18) vs 12m (1.11±0.23)<br><br>S3: 3m (1.41±0.16) vs 12m (1.04±0.21)<br><br>S4: 3m (1.46±0.22) vs 12m (0.95±0.09)	p = 0.963	

			Day 1<br>S1: 3m (1.28±0.43) vs 12m (1.44±0.22)<br><br>S2: 3m (1.74±0.51) vs 12m (2.07±0.41)<br><br>S3: 3m (2.93±0.45) vs 12m (2.13±0.30)<br><br>S4: 3m (0.70±0.15) vs 12m (1.99±0.32)	p = 0.808<br><br>p = 0.578<br><br>p = 0.243<br><br>p = 0.006	
			Day 2<br>S1: 3m (0.82±0.16) vs 12m (2.06±0.44)<br><br>S2: 3m (0.76±0.26) vs 12m (3.10±0.41)<br><br>S3: 3m (0.92±0.21) vs 12m (2.32±0.51)<br><br>S4: 3m (0.71±0.16) vs 12m (2.00±0.32)	p = 0.097<br><br>p = 0.005<br><br>p = 0.038<br><br>p = 0.039	
			Day 3<br>S1: 3m (1.23±0.15) vs 12m (1.93±0.38)<br><br>S2: 3m (1.11±0.24) vs 12m (2.58±0.41)<br><br>S3: 3m (0.84±0.16) vs 12m (2.16±0.39)<br><br>S4: 3m (0.94±0.21) vs 12m (2.33±0.33)	p = 0.307<br><br>p = 0.037<br><br>p = 0.036<br><br>p = 0.013	
			Day 6<br>S1: 3m (1.20±0.29) vs 12m (2.36±0.48)<br><br>S2: 3m (1.11±0.39) vs 12m (2.65±0.60)<br><br>S3: 3m (1.25±0.17) vs 12m (1.06±0.14)<br><br>S4: 3m (1.26±0.40) vs 12m (2.76±0.55)	p = 0.246<br><br>p = 0.097<br><br>p = 0.071<br><br>p = 0.110	
2k	Average AUC for spontaneous activity in 3 m and 12 m stimulated animals	One-Way ANOVA	3m<br>BL<br>S1 (1.24±0.21)<br>S2 (1.14±0.19)<br>S3 (1.15±0.18)<br>S4 (1.27±0.27)	p = 0.968	

	Calcium-mediated neuronal activity ($\Delta F/F_0/s$) – comparison of each session with the average BL (S1-4) within each age group	One-Way ANOVA with Holm-Šidák	3m<br>BL S1-4 (1.15±0.10)<br>vs<br>Day 1<br>S1 (0.99±0.19)<br>S2 (1.60±0.27)<br>S3 (2.27±0.28)<br>S4 (0.64±0.12)<br>Day 2<br>S1 (0.58±0.13)<br>S2 (1.27±0.36)<br>S3 (1.04±0.20)<br>S4 (0.61±0.11)<br>Day 3<br>S1 (0.69±0.14)<br>S2 (0.44±0.14)<br>S3 (0.54±0.12)<br>S4 (0.58±0.10)<br>Day 6<br>S1 (1.39±0.24)<br>S2 (0.99±0.18)<br>S3 (1.43±0.16)<br>S4 (1.07±0.25)	p = 0.428<br>p = 0.150<br>p < 0.001<br>p = 0.027<br><br>p = 0.028<br>p = 0.755<br>p = 0.710<br>p = 0.017<br><br>p = 0.011<br>p = 0.009<br>p = 0.007<br>p = 0.001<br><br>p = 0.346	
		One-Way ANOVA with Holm-Šidák	12m<br>BL<br>S1 (1.13±0.20)<br>S2 (0.72±0.16)<br>S3 (1.08±0.19)<br>S4 (0.75±0.09)	p = 0.132	
		One-Way ANOVA with Holm-Šidák	12m<br>BL S1-4 (0.86±0.07)<br>vs<br>Day 1<br>S1 (1.13±0.19)<br>S2 (2.07±0.43)<br>S3 (2.90±0.38)<br>S4 (1.77±0.27)<br>Day 2<br>S1 (1.85±0.32)<br>S2 (1.87±0.32)<br>S3 (1.88±0.35)<br>S4 (1.58±0.19)<br>Day 3<br>S1 (1.32±0.26)<br>S2 (1.64±0.26)<br>S3 (1.21±0.19)<br>S4 (2.09±0.24)<br>Day 6<br>S1 (1.88±0.26)<br>S2 (1.91±0.36)<br>S3 (1.81±0.28)<br>S4 (1.91±0.44)	p = 0.508<br>p = 0.018<br>p < 0.001<br>p = 0.031<br><br>p = 0.014<br>p = 0.008<br>p = 0.004<br>p = 0.016<br><br>p = 0.273<br>p = 0.020<br>p = 0.247<br>p < 0.001<br><br>p = 0.027<br>p = 0.033<br>p = 0.021<br>p = 0.011	
	Calcium-mediated neuronal activity ($\Delta F/F_0/s$)– comparison between 3m and 12m	Two-Way ANOVA with Holm-Šidák test	BL (0)<br>S1: 3m (1.24±0.21) vs 12m (1.13±0.20)<br><br>S2: 3m (1.14±0.19) vs 12m (0.72±0.16)<br><br>S3: 3m (1.15±0.18) vs 12m (1.08±0.19)<br><br>S4: 3m (1.27±0.27) vs 12m (0.75±0.09)	p = 0.498	

			Day 1<br>S1: 3m (0.99±0.19) vs 12m (1.13±0.19)<br><br>S2: 3m (1.60±0.27) vs 12m (2.07±0.43)<br><br>S3: 3m (2.27±0.28) vs 12m (2.90±0.38)<br><br>S4: 3m (0.64±0.12) vs 12m (1.77±0.27)	p = 0.827<br><br>p = 0.447<br><br>p = 0.279<br><br>p = 0.032	
			Day 2<br>S1: 3m (0.58±0.13) vs 12m (1.85±0.32)<br><br>S2: 3m (1.27±0.36) vs 12m (1.87±0.32)<br><br>S3: 3m (1.04±0.20) vs 12m (1.88±0.35)<br><br>S4: 3m (0.61±0.11) vs 12m (1.58±0.19)	p = 0.028<br><br>p = 0.330<br><br>p = 0.085<br><br>p = 0.036	
			Day 3<br>S1: 3m (0.69±0.14) vs 12m (1.32±0.26)<br><br>S2: 3m (0.44±0.14) vs 12m (1.64±0.26)<br><br>S3: 3m (0.54±0.12) vs 12m (1.21±0.19)<br><br>S4: 3m (0.58±0.10) vs 12m (2.09±0.24)	p = 0.214<br><br>p = 0.064<br><br>p = 0.213<br><br>p < 0.001	
			Day 6<br>S1: 3m (1.39±0.24) vs 12m (1.88±0.26)<br><br>S2: 3m (0.99±0.18) vs 12m (1.91±0.36)<br><br>S3: 3m (1.43±0.16) vs 12m (1.81±0.28)<br><br>S4: 3m (1.07±0.25) vs 12m (1.91±0.44)	p = 0.428<br><br>p = 0.108<br><br>p = 0.407<br><br>p = 0.248	
The data for Fig. 2 were obtained from 19 animals and 31 regions for panels d-f, and 6 animals for panels g-k. Data in panels f, g and h were normalized to Day 0. Two-sided tests were used throughout. Data are presented as mean ± S.E.M.					

Table S3. Statistical comparisons for Fig. 3.

Statistical comparisons for Fig. 3			Comparisons	Result	
**Panel**	**Description**	**Test**		**p value**	**n value**
3a	E:I ratio (the integral of mEPSP to mIPSP) for stimulated vs control groups at each age	Welch's t-test	3m: Con (1.04±0.15) vs<br>Stim (0.62±0.07)	p = 0.022	Neurons #<br><br>3m<br>Con = 16<br>Stim = 12<br><br>8m<br>Con = 15<br>Stim = 11<br><br>12m<br>Con = 10<br>Stim = 14
		Mann-Whitney rank sum test	8m: Con (0.93±0.15) vs<br>Stim (1.19±0.28)	p = 0.795	
			12m: Con (0.73±0.19) vs<br>Stim (1.35±0.29)	p = 0.038	
	E:I ratio comparison between stimulated age groups	KW One-Way ANOVA with Dunn's test	3m vs<br>8m<br>12m	 p = 0.204<br>p = 0.009	
	E:I ratio comparison between control age groups	KW One-Way ANOVA with Dunn's test	3m vs<br>8m<br>12m	p = 0.331	
3b	mIPSP frequency (Hz) for stimulated vs control groups at each age	t-test	3m: Con (1.79±0.20) vs<br>Stim (2.56±0.27)	p = 0.030	
		Mann-Whitney rank sum test	8m: Con (1.76±0.29) vs<br>Stim (1.77±0.31)	p = 0.959	
		t-test	12m: Con (2.40±0.18) vs<br>Stim (1.22±0.17)	p < 0.001	
	mIPSP frequency (Hz) comparisons between stimulated groups	One-Way ANOVA with Holm-Šidák test	3m vs<br>8m<br>12m	 p = 0.035<br>p < 0.001	
3c main	mEPSP amplitude (mV) comparisons between stimulated age groups and pooled control groups	KW One-Way ANOVA with Dunn's test	All groups<br><br>Stim 3m (0.22; 0.18-0.28) vs<br>Stim 8m (0.24; 0.19-0.32)<br>Stim 12m (0.27; 0.22-0.37)<br><br>Con (pooled ages) (0.23; 0.18-0.31)	p < 0.001<br><br><br>p < 0.001<br>p < 0.001	
3c inset	mEPSP amplitude (mV) for stimulated vs control groups at each age	Mann-Whitney rank sum test	3m: Con (0.26±0.04) vs<br>Stim (0.24±0.02)	p < 0.001	
			8m: Con (0.24±0.04) vs<br>Stim (0.27±0.05)	p < 0.001	
			12m: Con (0.27±0.04) vs<br>Stim (0.31±0.05)	p < 0.001	
3e main	Normalized (to control) VGAT puncta density between stimulated age groups and pooled control groups	KW One-Way ANOVA with Dunn's test	All groups<br><br>Stim 3m (1.08; 0.77-1.59) vs<br>Stim 8m (0.90; 0.71-1.18)<br>Stim 12m (0.79; 0.65-1.17)<br><br>Con (pooled ages) (0.94; 0.62-1.33)	p < 0.001<br><br><br>p < 0.001<br>p < 0.001	Neurons #<br>3m<br>Con = 249<br>Stim = 122<br>8m<br>Con = 132<br>Stim = 225<br>12m<br>Con = 263<br>Stim = 149
3e inset	Normalized (to control) VGAT puncta density on c-Fos+ neurons for stimulated vs control groups at each age	Mann-Whitney rank sum test	3m: Con (1.00±0.04) vs<br>Stim (1.22±0.05)	p < 0.001	
			8m: Con (1.00±0.04) vs<br>Stim (0.98±0.03)	p = 0.497	
		t-test	12m: Con (1.00±0.03) vs<br>Stim (0.92±0.03)	p = 0.043	
3f main	Normalized (to control) dendritic spine size from c-Fos+ neurons for stimulated groups (average per dendritic branch) and pooled control groups	One-Way ANOVA with Holm-Šidák test	All groups<br><br>Stim 3m (0.94; 0.76-1.05) vs<br>Stim 8m (1.05; 0.89-1.16)<br>Stim 12m (1.11; 1.03-1.19)<br><br>Con (pooled ages) (0.98; 0.86-1.10))	p = 0.002<br><br><br>p = 0.007<br>p = 0.002	Dendrites #<br>3m<br>Con = 84<br>Stim = 25<br>8m<br>Con = 26<br>Stim = 27<br>12m<br>Con = 91<br>Stim = 15
3f inset	Normalized (to control) dendritic spine size from c-Fos+ neurons for stimulated vs control groups at each age. (average per dendritic branch)	t-test	3m: Con (1.00±0.02) vs<br>Stim (0.92±0.03)	p = 0.032	
			8m: Con (1.00±0.04) vs<br>Stim (1.04±0.03)	p = 0.495	
			12m: Con (1.00±0.01) vs<br>Stim (1.11±0.03)	p = 0.015	

3h	Correlation between dendritic structural E:I ratio and normalized c-Fos intensity in the stimulated groups	Pearson correlation	(E:I; c-Fos)<br>3m<br>(0.59±0.04; 0.41±0.03)<br>(0.83±0.08; 0.51±0.04)<br>(0.76±0.05: 0.76±0.07)<br>8m<br>(1.22±0.08; 1.15±0.18)<br>(0.95±0.07; 1.25±0.08)<br>(0.83±0.08; 0.97±0.04)<br>(0.81±0.06; 0.89±0.06)<br>12m<br>(1.12±0.13; 1.48±0.12)<br>(1.49±0.15; 1.10±0.09)<br>(1.45±0.13; 1.23±0.08)	r = 0.70<br>p = 0.025	Animals #, Dendrites #<br>3m = 3, 55<br>8m = 4, 92<br>12m = 3, 45
The data for Fig. 3 were obtained from 54 animals for panels a-c, 18 animals for panels e-f and 10 animals for panel h. Data in panels e-f and h were normalized to the control groups. Two-sided tests were used throughout. Data presented as mean ± S.E.M, or as median and IQR.					

Table S4. Statistical comparisons for Fig. 4.

Statistical comparisons for Fig. 4			Comparisons	Result	
Panel	Description	Test		p value	n value
4f	Normalized (to baseline) spine activity (*$\Delta F/F_0$* integral) before and after overstimulation for 3 m and 12 m groups	Wilcoxon signed rank test	3m: BL (1.00±0.08) vs Post-stim (0.85±0.09)	p < 0.001	Spines # 3m = 539 12m = 434
			12m: BL (1.00±0.09) vs Post-stim (1.34±0.10)	p < 0.001	
4g	Normalized (to baseline) dendrite vs spine activity (*$\Delta F/F_0$* integral) for branches showing increased activity, in stimulated 3 m vs 12 m groups	t-test	Dendrite 1.1: 3m (0.89±0.19) vs 12m (0.92±0.15)	p = 0.897	Spines # 3m = 448 12m = 226
			Dendrite 1.2: 3m (0.78±0.15) vs 12m (1.47±0.20)	p = 0.009	
			Dendrite 1.3: 3m (0.62±0.09) vs 12m (1.86±0.38)	p < 0.001	
		Pearson correlation	3m correlation: r = -0.99	p = 0.012	
			12m correlation: r = 0.98	p = 0.021	
4i	Correlation between visual responsivity (%) and normalized (to baseline) spine activity (*$\Delta F/F_0$* integral) after overstimulation in 3 m and 12 m groups	Pearson correlation	3m correlation: r = 0.48	p < 0.001	Spines # 3m = 531 12m = 428
			12m correlation: r = 0.61	p < 0.001	
4j	Normalized (to baseline) spine activity after overstimulation in 3 m and 12 m groups	Mann-Whitney rank sum test	Visual: 3m (0.95; 0.47-1.54) vs 12m (1.68; 1.12-2.16)	p < 0.001	Spine # Visual 3m = 119 12m = 94 Non-visual 3m = 420 12m = 340
			Non-visual: 3m (0.35; 0.12-0.71) vs 12m (0.90; 0.56-1.12)	p < 0.001	
4k	Percentage (%) of visual and non-visual spines in stimulated 3 m and 12 m groups	Chi-square	Visual: 3m (22.1%) vs 12m (21.7%)	p = 0.976	
			Non-visual: 3m (77.9%) vs 12m (78.3%)	p = 0.963	
The data for Fig. 4 were obtained from 8 animals (13 regions) for panels a-k. Animals were imaged at baseline (BL) and after overstimulation (post-stim). Data in panels f, g, i and j were normalized to the baseline imaging session. Two-sided tests were used throughout. Data presented as mean ± S.E.M, except for panels j, where median and interquartile range (IQR) are reported.					

Table S5. Statistical comparisons for Fig. 5.

Statistical comparisons for Fig. 5			Comparisons	Result	
**Panel**	**Description**	**Test**		**p value**	**n value**
5c	Normalized (to baseline) activity (*$\Delta F/F_0$* integral) of inhibitory neurons at baseline and after stimulation in 3 m animals	Wilcoxon signed rank test	Inhibitory: BL (1.00±0.05) vs Post-stim (0.98±0.06)	p = 0.732	Neurons # 3m = 39 12m = 95
	Normalized (to baseline) activity (*$\Delta F/F_0$* integral) of inhibitory neurons at baseline and after stimulation in 12 m animals	Paired t-test	Inhibitory: BL (1.00±0.05) vs Post-stim (1.34±0.04)	p < 0.001	
5f	Normalized (to baseline) correlation between Exc-Exc cells at baseline and after stimulation in 3m animals	Wilcoxon signed rank test	Exc-Exc: BL (1.00±0.25) vs Post-stim (1.00±0.13)	p = 0.465	Exc-Exc # 42
	Normalized (to baseline) correlation between Exc-Inh cells at baseline and after stimulation in 3m animals	Paired t-test	Exc-Inh: BL (1.00±0.26) vs Post-stim (1.5±0.23)	p = 0.011	Exc-Inh # 42
5h	Normalized (to baseline) correlation between Exc-Exc cells at baseline and after stimulation in 12m animals	Wilcoxon signed rank test	Exc-Exc: BL (1.00±0.24) vs Post-stim (1.56±0.22)	p = 0.046	Exc-Exc # 45
	Normalized (to baseline) correlation between Exc-Inh cells at baseline and after stimulation in 12m animals	Paired t-test	Exc-Inh: BL (1.00±0.19) vs Post-stim (1.11±0.18)	p = 0.680	Exc-Inh # 45
The data for Fig. 5 were obtained from 8 animals imaged at baseline (BL) and after overstimulation (Post-stim). Data in panels c, f and h were normalized to the baseline imaging session. Two-sided tests were used throughout. Data are presented as mean ± S.E.M.					

Table S6. Statistical comparisons for Fig. 6.

Statistical comparisons for Fig. 5			Comparisons	Result	
**Panel**	**Description**	**Test**		**p value**	**n value**
6c	Learning rate for control vs stimulated mice at each age group	Two-Way ANOVA with Holm-Šidák<br><br>Group comparison within each age category	3m: Con (0.11; 0.07-0.12) vs Stim (0.11; 0.08-0.12)<br><br>8m: Con (0.12; 0.08-0.15) vs Stim (0.06; 0.05-0.11)<br><br>12m Con (0.08; 0.05-0.13) vs Stim (0.02; 0.01-0.06)	p = 0.951<br><br>p = 0.019<br><br>p = 0.002	Mice #<br>3m<br>Con = 12<br>Stim = 8<br>8m<br>Con = 11<br>Stim = 8<br>12m<br>Con = 8<br>Stim = 7
		Age comparison within each group category	Stim:<br>3m vs 8m<br>3m vs 12m<br>8m vs 12m	 p = 0.115<br>p < 0.001<br>p = 0.011	
			Con:<br>3m vs 8m<br>3m vs 12m<br>8m vs 12m	 p = 0.480<br>p = 0.309<br>p = 0.142	
	Correlation between learning rate and age	Pearson's correlation	Stim: 3m vs 8m vs 12m<br>r = -0.757<br><br>Con: 3m vs 8m vs 12m<br>r = -0.176	p < 0.001<br><br>p = 0.345	Mice #<br>Stim = 23<br><br>Con = 31
6d	Percentage (%) of c-Fos+ neurons in the PFC of stimulated animals and pooled control groups	KW One-Way ANOVA with Dunn's test	Con (40; 30-50) vs<br>3m (30; 20-40)<br>12m (50; 40-60)	 p < 0.001<br>p < 0.001	Neurons #<br>500 per group
6e	Comparisons of learning rate on the rCPT task for 3m intervention groups	Two-Way ANOVA with Holm-Šidák	Con (0.12; 0.08-0.13)<br>vs<br>Stim (0.11; 0.08-0.12)<br><br>MTEP + Con (0.11; 0.09-0.11)<br>vs<br>MTEP + Stim (0.04; 0.03-0.08)<br><br>Con (0.12; 0.08-0.13)<br>vs<br>MTEP + Con (0.11; 0.09-0.11)<br><br>Stim (0.11; 0.08-0.12)<br>vs<br>MTEP + Stim (0.04; 0.03-0.08)	p = 0.369<br><br>p = 0.025<br><br>p = 0.416<br><br>p = 0.009	Mice #<br>V+C = 8<br>V+S = 8<br>M+C = 4<br>M+S = 4
6f	Comparisons of learning rate on the rCPT task for 12m intervention groups	Two-Way ANOVA with Holm-Šidák	Con (0.08; 0.05-0.13)<br>vs<br>Stim (0.02; 0.01-0.06)<br><br>PAM + Con (0.08; 0.02-0.11)<br>vs<br>PAM + Stim (0.06; 0.05-0.08)<br><br>Con (0.08; 0.05-0.13)<br>vs<br>PAM + Con (0.08; 0.02-0.11)<br><br>Stim (0.02; 0.01-0.06)<br>vs<br>PAM + Stim (0.06; 0.05-0.08)	p = 0.010<br><br>p = 0.960<br><br>p = 0.517<br><br>p = 0.047	Mice #<br>C = 8<br>S = 7<br>P+C = 8<br>P+S = 8
		t-test	Con (0.08; 0.05-0.13)<br>vs<br>PAM + Stim (0.06; 0.05-0.08)	p = 0.473	
The data for Fig. 6 were obtained from 54 animals for panel c, 18 animals for panel d, 24 animals for panel e and 29 animals for panel f. Two-sided tests were used throughout. Data reported as median and interquartile range (IQR).					

Supplementary Figures

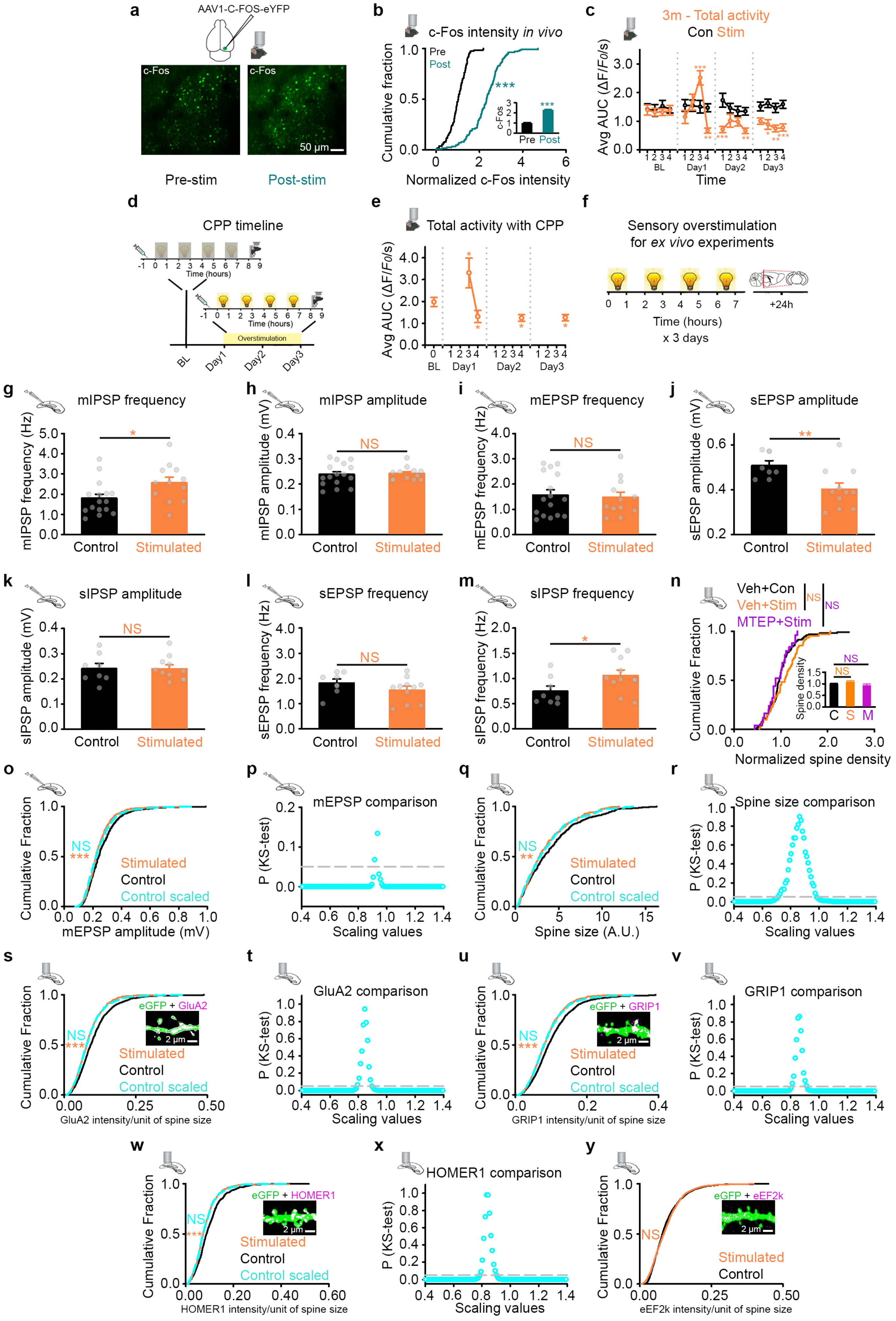
a
AAV1-C-FOS-eYFP
c-Fos
c-Fos
50 µm
Pre-stim
Post-stim
b
c-Fos intensity in vivo
Pre
Post
Cumulative fraction
Normalized c-Fos intensity
c-Fos
c
3m - Total activity
Con Stim
Avg AUC (ΔF/Fo/s)
BL
Day1
Day2
Day3
Time
d
CPP timeline
Time (hours)
Overstimulation
BL
Day1
Day2
Day3
e
Total activity with CPP
f
Sensory overstimulation
for ex vivo experiments
+24h
Time (hours)
x 3 days
g
mIPSP frequency
mIPSP frequency (Hz)
Control
Stimulated
h
mIPSP amplitude
mIPSP amplitude (mV)
i
mEPSP frequency
mEPSP frequency (Hz)
j
sEPSP amplitude
sEPSP amplitude (mV)
k
sIPSP amplitude
sIPSP amplitude (mV)
l
sEPSP frequency
sEPSP frequency (Hz)
m
sIPSP frequency
sIPSP frequency (Hz)
n
Veh+Con
Veh+Stim
MTEP+Stim
Cumulative Fraction
Spine density
Normalized spine density
o
Stimulated
Control
Control scaled
mEPSP amplitude (mV)
p
mEPSP comparison
P (KS-test)
Scaling values
q
Spine size (A.U.)
r
Spine size comparison
s
eGFP + GluA2
2 µm
GluA2 intensity/unit of spine size
t
GluA2 comparison
u
eGFP + GRIP1
GRIP1 intensity/unit of spine size
v
GRIP1 comparison
w
eGFP + HOMER1
HOMER1 intensity/unit of spine size
x
HOMER1 comparison
y
eGFP + eEF2k
eEF2k intensity/unit of spine size

Figure S1

Supplementary Fig. 1 | Overstimulation drives functional, structural and molecular plasticity in young adult animals.

a, Diagram showing injection of AAV1-C-FOS-eYFP in V1 and 2-P *in vivo* images of c-Fos labelled L2/3 pyramidal neurons pre-stimulation (left) and after 2x1h bouts of overstimulation (right). Scale bar: 50µm. **b**, Normalized c-Fos intensity scores pre-stimulation (black) and after 2x1h bouts of overstimulation (cyan) (MWRST, $P<0.001$). Inset shows average values. **c**, Calcium-mediated neuronal activity from overstimulated (orange) and control (black) mice across more fine-scale imaging time-course. Absence of asterisks denote no significant differences between control and stimulated animals. **d**, Timeline depicting CPP delivery relative to baseline (BL) or overstimulation (Days 1-3). **e,** Activity from overstimulated mice receiving CPP injections as in (**d**). **f**, Timeline of overstimulation and tissue collection for *ex vivo* experiments. **g-m**, mIPSP frequency (Hz; t-test, $P=0.030$) (**g**), mIPSP amplitude (mV; t-test, $P=0.808$) (**h**), mEPSP frequency (Hz; MWRST, $P=0.871$) (**i**), sEPSP (**j,l**) and sIPSP (**k,m**) amplitude (mV; t-tests; sEPSP, $P=0.009$; sIPSP, $P=0.995$) (**j,k**), and frequency (Hz; t-tests; sEPSP, $P=0.215$; sIPSP, $P=0.049$) (**l,m**) for electrophysiology recordings of L2/3 excitatory neurons in stimulated (orange) and control (black) animals. **n**, *Thy1*-eGFP spine density for vehicle (control, black, ‘C’), vehicle and stimulation (orange, ‘S’), or MTEP and stimulation (purple, ‘M’) normalized to control (black, ‘C’) (Kruskal-Wallis (KW) One-Way ANOVA, $P=0.097$). **o-x**, Cumulative fraction of mEPSP amplitude (KW One-Way ANOVA with Dunn’s test, Con vs Stim, $P<0.001$) (**o**), spine size (One-Way ANOVA with Holm-Šidák, Con vs Stim, $P=0.009$) (**q**), GluA2 (KW One-Way ANOVA with Dunn’s test, Con vs Stim, $P<0.001$) (**s**), GRIP1 (KW One-Way ANOVA with Dunn’s test, Con vs Stim, $P<0.001$) (**u**), and HOMER1 (KW One-Way ANOVA with Dunn’s test, Con vs Stim, $P<0.001$) (**w**). Protein values per unit of GFP positive spine size in overstimulated (orange) and control (black) 3m mice. For each plot, cyan dashed line depicts control values scaled to stimulated distributions (**p,r,t,v,x**). Insets show representative *Thy1*-eGFP+ dendrites (green) from a V1 L2/3 excitatory neuron with immunofluorescence labelled GluA2 (purple, **s**), GRIP1 (purple, **u**), and HOMER1 (purple, **w**) colocalization (white). Scale bars: 2µm. **y**, Cumulative fraction of eEf2k intensity

values per unit of spine size from overstimulated (orange) and control (black) mice (MWRST, $P=0.465$). Inset shows representative *Thy1*-eGFP+ dendrite (green) from a V1 L2/3 excitatory neuron and eEF2k puncta (purple). Scale bar: 2µm. Data from 3 animals for panels a-b and d-e, 6 animals for panel c, 19 animals for panels g-i, o and p, 8 animals for panels j-m, 9 animals for panels n, q and r, and 8 animals for panels s-y. In all panels, *$P<0.05$, **$P<0.01$, ***$P<0.001$, and NS. Two-sided tests were used throughout, for detailed statistical reporting see Table S7. Data are presented as mean ± s.e.m., with individual data points (gray dots), or as a cumulative distribution.

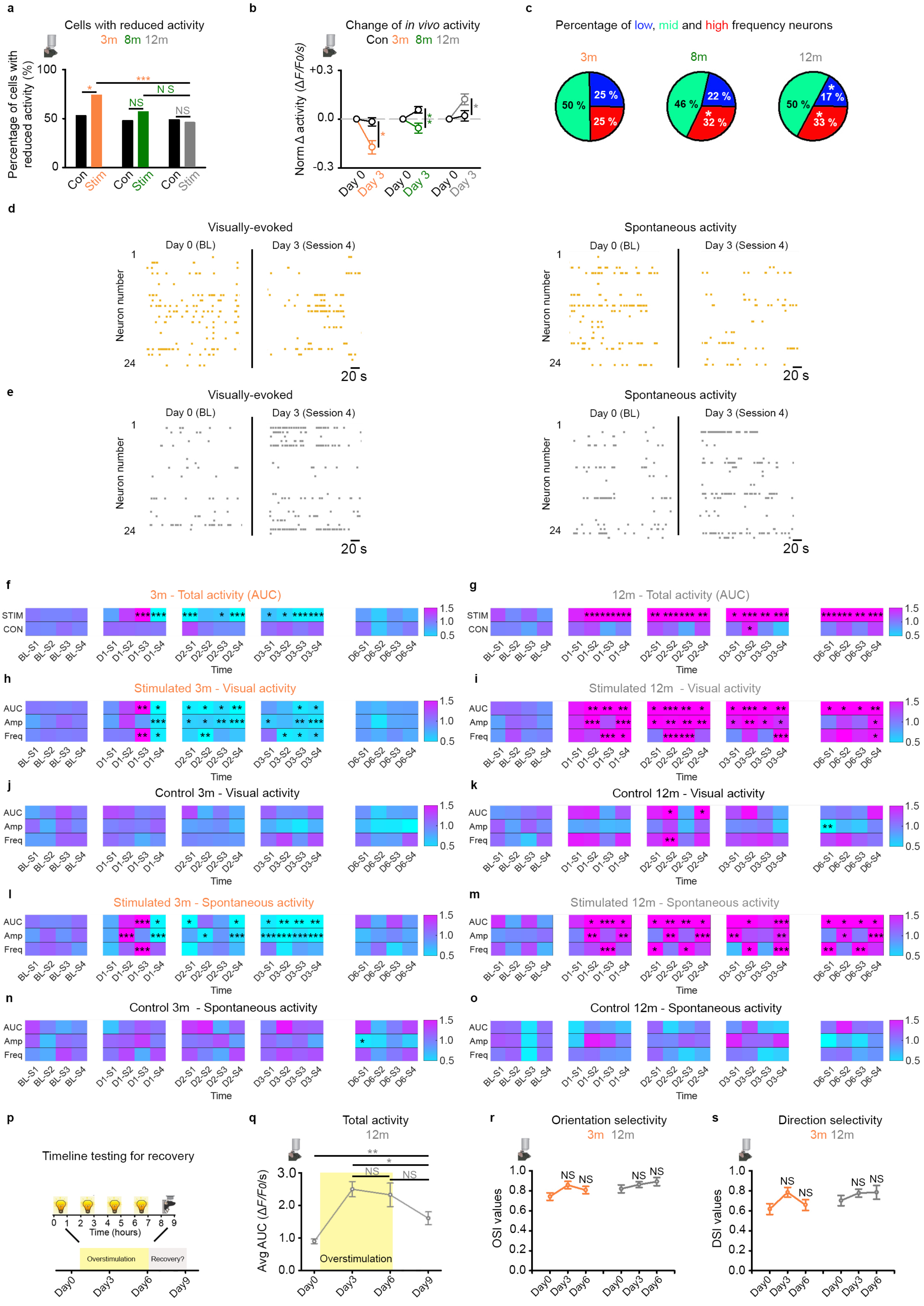
a
Cells with reduced activity
3m 8m 12m
Percentage of cells with reduced activity (%)
Con Stim Con Stim Con Stim
b
Change of in vivo activity
Con 3m 8m 12m
Norm Δ activity (ΔF/F0/s)
Day 0 Day 3
c
Percentage of low, mid and high frequency neurons
3m 8m 12m
d
Visually-evoked
Spontaneous activity
Day 0 (BL)
Day 3 (Session 4)
Neuron number
20 s
e
f
3m - Total activity (AUC)
g
12m - Total activity (AUC)
STIM
CON
Time
h
Stimulated 3m - Visual activity
i
Stimulated 12m - Visual activity
j
Control 3m - Visual activity
k
Control 12m - Visual activity
l
Stimulated 3m - Spontaneous activity
m
Stimulated 12m - Spontaneous activity
n
Control 3m - Spontaneous activity
o
Control 12m - Spontaneous activity
AUC
Amp
Freq
p
Timeline testing for recovery
Time (hours)
Overstimulation
Recovery?
q
Total activity
12m
Avg AUC (ΔF/F0/s)
r
Orientation selectivity
3m 12m
OSI values
s
Direction selectivity
3m 12m
DSI values

Figure S2

Supplementary Fig. 2 | Age-related changes in the functional neuronal activity response to overstimulation.

a, Percentage of V1 L2/3 neurons in anaesthetized animals showing reduced activity after overstimulation at 3m (orange), 8m (green) and 12m (gray) compared to age-matched controls (black) (Chi-square, Con vs Stim, 3m, P=0.023; 8m, P=0.052; 12m, P=0.663; Chi-Square, Stim: 3m vs 12m, P<0.001). **b**, Normalized (to Day0) change in calcium-mediated activity ($\Delta F/F_0$ integral/sec) compared to Day0 (before overstimulation, BL) for 3m (orange), 8m (green) and 12m (gray) overstimulated mice and age-matched controls (black) (Two-Way ANOVA, Con vs Stim, 3m, P=0.010; 8m, P=0.002; 12m, P=0.032). **c**, Pie charts for 3m, 8m and 12m non-stimulated animals showing percentage of neurons with low-(blue, min-Q1), mid-(green, Q2-Q3) and high-(red, >Q3) frequency calcium transients. **d,e,** Example raster plots of visually-evoked and spontaneous activity at Day0 (BL) and Day3 (S4) for 3m (orange, **d**) and 12m (gray, **e**) mice. Points denote calcium transient peak. Scale bar: 20s. **f,g**, Heatmaps from anaesthetized animals showing total activity (AUC) changes for 3m (orange, **f**), and 12m (gray, **g**) overstimulated (stimulated, top) and control (control, bottom) animals in relation to the average BL activity. **h-o**, AUC, amplitude and frequency heatmaps of calcium-mediated transients over the fine-scale imaging time-course during conditions of visually-evoked (**h-k**) or spontaneous resting-state (**l-o**) activity in 3m (orange, stimulated, **h,l**; black, control; **j,n**), and 12m (gray, stimulated, **i,m**; black, control, **k,o**) animals. Colour bar denotes normalized change in activity in relation to BL (**f-o**). See Table S8 for additional comparisons and raw values. Asterisks show significant differences between each session and the average BL (S1-4), while absence of asterisks signifies NS (**f-o**). **p**, Timeline testing for recovery of neuronal activity levels in 12m mice after overstimulation, with timepoints at Day0, during the overstimulation period (Day3 and Day6), and 3 days after overstimulation (Day9). **q**, Average AUC ($\Delta F/F_0$ integral/sec) for 12m mice at S4 of Day 0, 3, 6 and 9 (t-tests, Day9 vs Day0, P=0.001; vs Day3, P=0.010; vs Day6, P=0.111; Day3 vs Day6, P=0.676). **r,s**, Average orientation (OSI, **r**) and direction (DSI, **s**) selectivity indices measured before (Day0) and after overstimulation on Day3 and Day6 in 3m (orange) and 12m (gray) adult anaesthetized mice

(One-way ANOVA, OSI: 3m, $P=0.091$; 12m, $P=0.439$; DSI: 3m, $P=0.073$; 12m, $P=0.458$). Data from 19 animals and 31 regions for panels a-c, 12 animals for panels f-o, 3 animals for panel q, and 6 animals for panels r-s. In all panels, *$P<0.05$, **$P<0.01$, ***$P<0.001$, and NS, not significant. Two-sided tests were used throughout, for detailed statistical reporting see Table S8. Data are presented as percentage, as mean ± s.e.m., or as heatmaps.

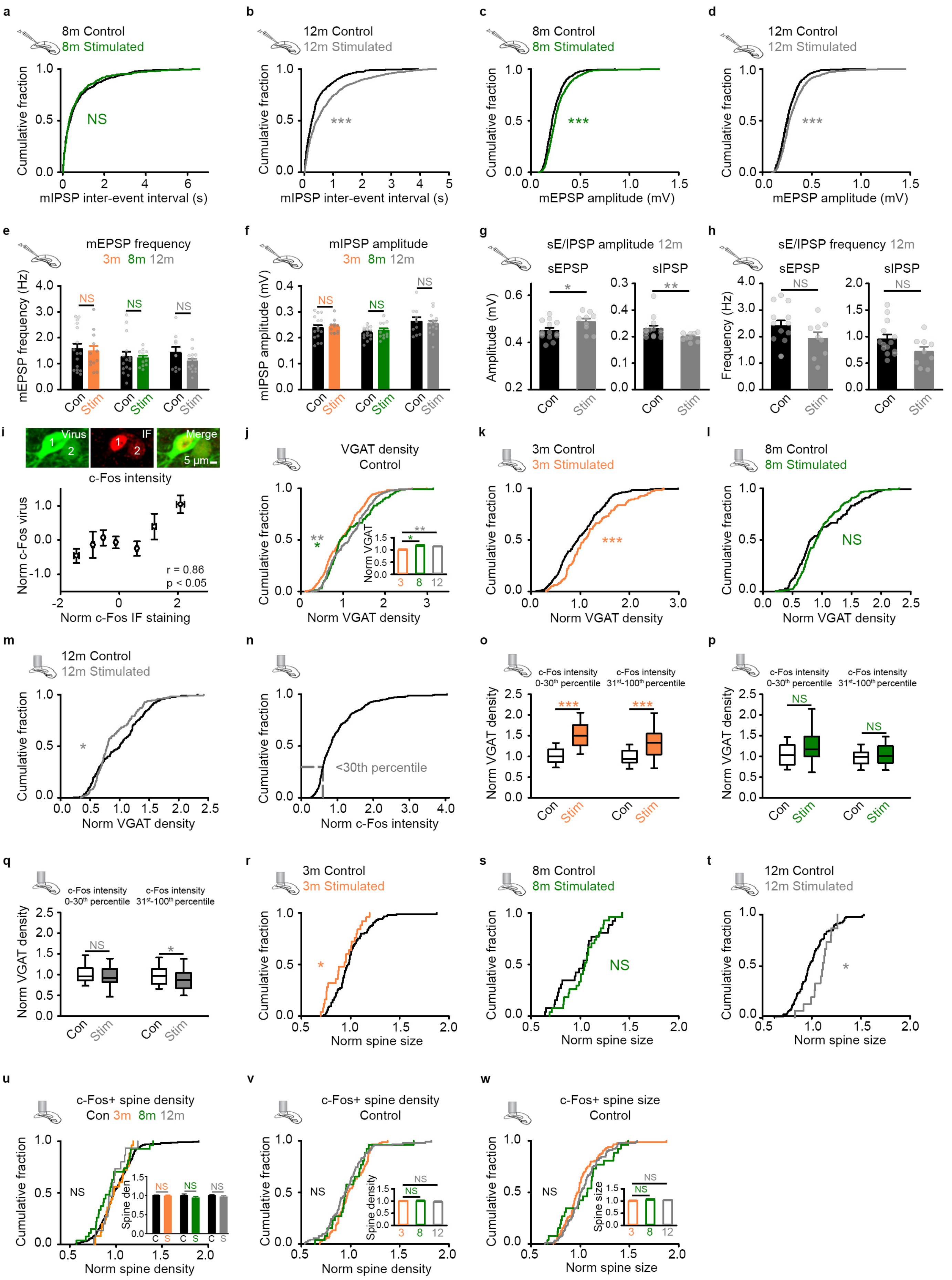

a
8m Control
8m Stimulated
Cumulative fraction
NS
mIPSP inter-event interval (s)
b
12m Control
12m Stimulated
***
mIPSP inter-event interval (s)
c
8m Control
8m Stimulated
***
mEPSP amplitude (mV)
d
12m Control
12m Stimulated
***
mEPSP amplitude (mV)
e
mEPSP frequency
3m 8m 12m
mEPSP frequency (Hz)
Con Stim
f
mIPSP amplitude
3m 8m 12m
mIPSP amplitude (mV)
g
sE/IPSP amplitude 12m
sEPSP
sIPSP
Amplitude (mV)
h
sE/IPSP frequency 12m
Frequency (Hz)
i
Virus
IF
Merge
5 μm
c-Fos intensity
Norm c-Fos virus
r = 0.86
p < 0.05
Norm c-Fos IF staining
j
VGAT density
Control
Norm VGAT
Norm VGAT density
k
3m Control
3m Stimulated
l
8m Control
8m Stimulated
m
12m Control
12m Stimulated
n
<30th percentile
Norm c-Fos intensity
o
c-Fos intensity 0-30th percentile
c-Fos intensity 31st-100th percentile
Norm VGAT density
p
q
r
3m Control
3m Stimulated
Norm spine size
s
8m Control
8m Stimulated
t
12m Control
12m Stimulated
u
c-Fos+ spine density
Con 3m 8m 12m
Spine den
Norm spine density
v
c-Fos+ spine density
Control
Spine density
w
c-Fos+ spine size
Control
Spine size
Norm spine size

Figure S3

Supplementary Fig. 3 | Age-related changes in synaptic and neuronal plasticity response following overstimulation.

a-b, Cumulative distribution of mIPSP inter-event interval (IEI, in seconds) for 8m (**a**, green; MWRST, $P=0.569$) and 12m (**b**, gray; MWRST, $P<0.001$) overstimulated animals and age-matched controls (black). **c-d**, Cumulative distribution of mEPSP amplitude (mV) for 8m (**c**, green; MWRST, $P<0.001$) and 12m (**d**, gray; MWRST, $P<0.001$) overstimulated animals and age-matched controls (black). See **Fig.1e-f** for 3m control and overstimulated comparisons. **e-f**, mEPSP frequency (Hz) (**e**) and mIPSP amplitude (mV) (**f**) for 3m (orange), 8m (green) and 12m (gray) overstimulated and age-matched controls (black) (mEPSP: MWRST, Con vs Stim, 3m: $P=0.871$; 8m: $P=0.678$; t-test, 12m: $P=0.138$; mIPSP: t-tests, Con vs Stim, 3m: $P=0.808$; 8m: $P=0.397$; 12m: $P=0.699$). **g-h**, sE/IPSP amplitude (mV, **g**) and sE/IPSP frequency (Hz, **h**) in overstimulated (gray) and control (black) 12m animals (amplitude: sEPSP, t-test, $P=0.044$; sIPSP, MWRST, $P=0.007$; frequency: sEPSP, t-test, $P=0.132$; sIPSP, t-test, $P=0.076$). **i**, Correlation between c-Fos intensity for neurons labelled with the c-Fos antibody (x-axis) and neurons expressing eYFP under the c-Fos promoter via viral labelling (y-axis) (Pearson correlation, r=0.86, $P=0.014$). Top: *ex vivo* cortical slice showing neurons expressing either high (#1) or low (#2) levels of c-Fos using the viral labelling strategy (green, left), immunofluorescence labelling (red, middle), or both (right). Scale bar: 5µm. **j**, Distribution (main) and average (inset) VGAT density at c-Fos positive neurons for 3m (orange, open), 8m (green, open) and 12m (gray, open) control mice (normalized to 3m; Kruskal-Wallis One-Way ANOVA, 3m vs 8m, $P=0.020$; 3m vs 12m, $P=0.003$). **k-m**, Distribution of normalized VGAT density at c-Fos positive neurons for 3m (**k**, orange; MWRST, $P<0.001$), 8m (**l**, green; MWRST, $P=0.497$) and 12m (**m**, gray**;** t-test, $P=0.043$) overstimulated animals normalized to age-matched controls (black). **n**, Normalized c-Fos intensity values with dashed gray line depicting values $<30^{th}$ percentile. **o-q**, Normalized VGAT density for neurons expressing c-Fos at levels lower (left) and greater (right) than the 30th percentile for 3m (**o**, orange), 8m (**p**, green) and 12m (**q**, gray) overstimulated mice and controls (white, open) (MWRST, Con vs Stim, $<30^{th}$, 3m: $P<0.001$; 8m: $P=0.114$; 12m: $P=0.461$; $>30^{th}$, 3m: $P<0.001$; 8m: $P=0.226$;

12m: *P*=0.026). **r-t**, Dendritic spine size at c-Fos positive neurons for 3m (**r**, orange; t-test, *P*=0.032), 8m (**s**, green; t-test, *P*=0.495) and 12m (**t**, gray; t-test, *P*=0.015) overstimulated animals normalized to age-matched controls (black). **u**, Normalized dendritic spine density at c-Fos positive neurons for every overstimulated age group (3, orange; 8, green; 12, gray) and pooled controls (black) (One-Way ANOVA, *P*=0.363); inset shows average for each overstimulated group (inset, 'S') and age-matched controls (inset, 'C'). **v,w**, Distribution (main) and average (inset) of normalized dendritic spine density (**v**) and size (**w**) at c-Fos positive neurons for 3m (orange), 8m (green) and 12m (gray) control mice (normalized to 3m) (Kruskal-Wallis One-Way ANOVA, density, *P*=0.169; size, *P*=0.259). Data from 54 animals for panels a-f, 10 animals for panels g and h, 3 animals for panel i, and 18 animals for panels j-w. In all panels, **P*<0.05, ***P*<0.01, ****P*<0.001, and NS, not significant. Two-sided tests were used throughout, for detailed statistical reporting see Table S9. Data are presented as mean ± s.e.m. with individual data points (gray dots), as a cumulative distribution, or as median and IQR (box plots, box = quartiles, whiskers = 10th and 90th percentiles).

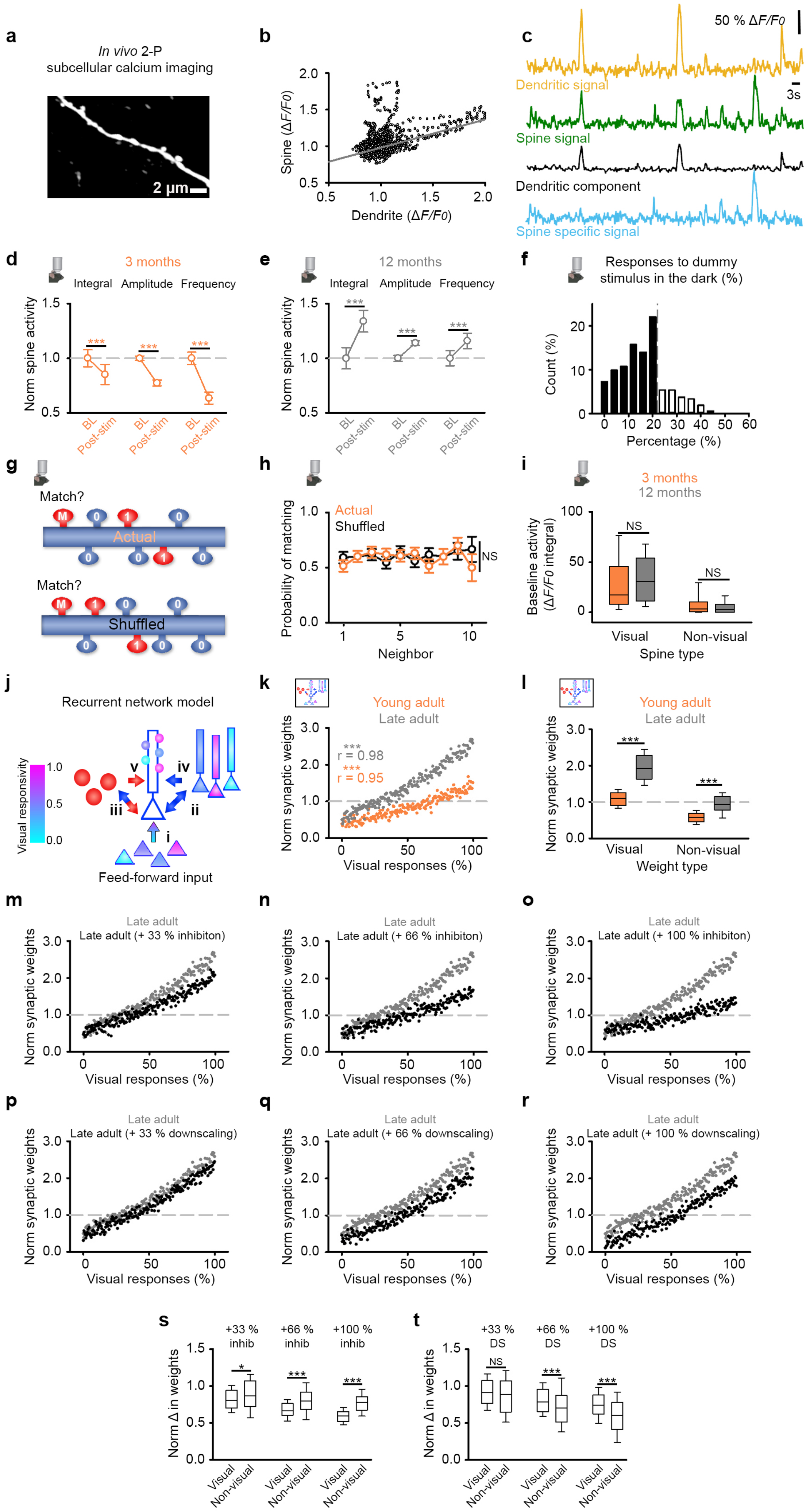
a
In vivo 2-P subcellular calcium imaging
2 µm
b
Spine (ΔF/F0)
Dendrite (ΔF/F0)
c
50 % ΔF/F0
3s
Dendritic signal
Spine signal
Dendritic component
Spine specific signal
d
3 months
Integral Amplitude Frequency
Norm spine activity
BL
Post-stim
e
12 months
f
Responses to dummy stimulus in the dark (%)
Count (%)
Percentage (%)
g
Match?
Actual
Shuffled
h
Probability of matching
Actual
Shuffled
NS
Neighbor
i
3 months
12 months
Baseline activity (ΔF/F0 integral)
NS
Visual
Non-visual
Spine type
j
Recurrent network model
Visual responsivity
Feed-forward input
k
Young adult
Late adult
r = 0.98
r = 0.95
Norm synaptic weights
Visual responses (%)
l
Weight type
m
Late adult (+ 33 % inhibiton)
n
Late adult (+ 66 % inhibiton)
o
Late adult (+ 100 % inhibiton)
p
Late adult (+ 33 % downscaling)
q
Late adult (+ 66 % downscaling)
r
Late adult (+ 100 % downscaling)
s
+33 % inhib
+66 % inhib
+100 % inhib
Norm Δ in weights
t
+33 % DS
+66 % DS
+100 % DS

Figure S4

Supplementary Fig. 4 | The functional dendritic spine response to sensory overstimulation is modified by age *in vivo*.

a, Representative image of *in vivo* 2-P subcellular calcium imaging in an anaesthetized animal showing an active stretch of dendrite from a GCaMP6s expressing neuron. Scale bar: 2µm. **b**, Correlation between recorded dendritic and spine activity ($\Delta F/F_0$ integral) for an example spine. **c**, Example of representative $\Delta F/F_0$ calcium trace for dendritic signal, spine signal, scaled dendritic component and spine specific signal. Scale bars: 50% $\Delta F/F_0$ and 3s. **d-e**, Normalized (to baseline) spine integral, amplitude and frequency in 3m (**d**, orange) and 12m (**e**, gray) animals at baseline (BL) and after overstimulation (Post-Stim) (Wilcoxon signed rank tests, Integral, 3m: $P<0.001$; 12m: $P<0.001$; Amplitude, 3m: $P<0.001$; 12m: $P<0.001$; Frequency, 3m: $P<0.001$; 12m: $P<0.001$). **f**, Histogram of time-locked responses to dummy visual stimulus in the dark (see Methods). Filled bars show 80 % of the distribution; open bars show false positive responses. Gray dashed line denotes false positive cut-off. **g**, Spatial clustering diagram depicting clustering analysis with example of actual (top) and shuffled (bottom) spine response positions. Spine of interest response (M) matching with red spines (match = 1), but not with blue spines (match = 0). **h**, Probability of response matching with up to 10 neighbours for actual (orange) and shuffled (black) spine positions (MWRST, $P=0.326$). **i**, Baseline spine activity ($\Delta F/F_0$ integral) prior to overstimulation for visual (left) and non-visual (right) spines from 3m (orange) and 12m (gray) animals (MWRST, visual, $P=0.073$; non-visual, $P=0.089$). **j**, Two-compartment model receiving sensory feedforward drive (i) and local recurrent inputs arising from excitatory (ii, iv) and inhibitory (iii, v) presynaptic sources on soma and dendrites. The model includes a two-term synaptic plasticity rule, incorporating both homeostatic and Hebbian plasticity. **k**, Synaptic weights (normalized to baseline) for synapses with different levels of visual responsivity after simulated overstimulation (Pearson correlation, young, $r=0.95$, $P<0.001$; late, $r=0.98$, $P<0.001$). Dashed gray line denotes no change in activity following *in silico* overstimulation. **l**, Synaptic weights for visual (>50% responsivity, left) and non-visual (<50% responsivity, right) inputs in response to simulated overstimulation (normalized to baseline session) (Welch's t-test, visual, $P<0.001$; non-visual, $P<0.001$). **m-r**,

Synaptic weight change (normalized to baseline) for synapses with different levels of visual responsivity after simulated overstimulation for late adult (gray dots), and late adult simulation where either inhibitory plasticity (**m-o**) or downscaling (**p-r**) were progressively reinstated (black; +33%, **m, p**; +66%, **n, q**; +100%, **o, r**). **s-t**, Normalized change (relative to late adult model) in the plasticity of synaptic weights for visual or non-visual responses that occurred following modulation of either inhibition (**s**) or downscaling (**t**) (MWRST; inhibition, +33%, P=0.036; +66%, P<0.001; +100%, P<0.001; downscaling, +33%, P=0.213; +66%, P<0.001; +100%, P<0.001). Data from 8 animals and 13 regions for panels a-i. In all panels, *P<0.05, ***P<0.001, and NS, not significant. Two-sided tests were used throughout, for detailed statistical reporting see Table S10. Data are presented as mean ± s.e.m., or as median and IQR (box plots, box = quartiles, whiskers = 10th and 90th percentiles).

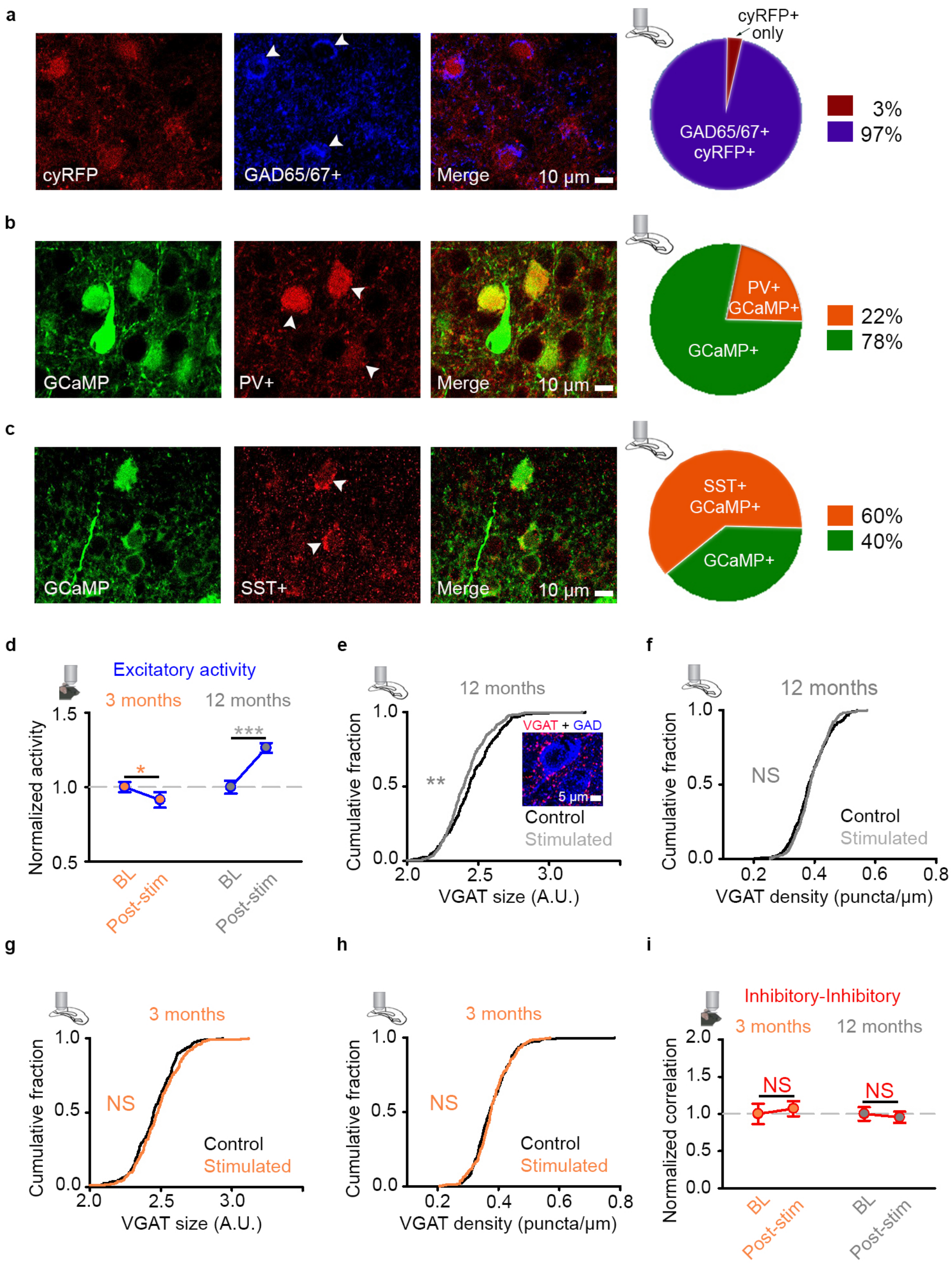
a
cyRFP
GAD65/67+
Merge
10 μm
cyRFP+ only
GAD65/67+ cyRFP+
3%
97%
b
GCaMP
PV+
Merge
10 μm
PV+ GCaMP+
GCaMP+
22%
78%
c
GCaMP
SST+
Merge
10 μm
SST+ GCaMP+
GCaMP+
60%
40%
d
Excitatory activity
3 months
12 months
Normalized activity
BL
Post-stim
e
12 months
Cumulative fraction
VGAT + GAD
5 μm
Control
Stimulated
VGAT size (A.U.)
f
12 months
NS
Control
Stimulated
VGAT density (puncta/μm)
g
3 months
NS
Control
Stimulated
VGAT size (A.U.)
h
3 months
NS
Control
Stimulated
VGAT density (puncta/μm)
i
Inhibitory-Inhibitory
3 months
12 months
Normalized correlation
NS
BL
Post-stim

Figure S5

Supplementary Fig. 5 | Age-related changes in the inhibitory response to overstimulation.

a-c, Inhibitory neurons labelled by the pAAV-mDlx-cyRFP1-GCaMP6S construct and co-labelling using immunofluorescence staining (see Methods). Representative images and pie charts showing fraction of double labelled cyRFP+ and GAD65/67+ positive neurons (~97%, **a**), GCaMP+ and parvalbumin positive (PV+) neurons (~22%, **b**), and GCaMP+ and somatostatin positive (SST+) neurons (~60%, **c**). Scale bars: 10µm. **d**, Normalized (to baseline) calcium-mediated neuronal activity ($\Delta F/F_0$ integral) from excitatory (blue) neurons in 3m (left, filled orange) and 12m (right, filled gray) animals at baseline (BL) and following overstimulation (Post-stim) (Paired t-tests, excitatory activity, BL vs Post-stim, 3m: P=0.040; 12m: P<0.001). **e-h**, Size (**e**,**g**) and density (**f**,**h**) of VGAT puncta at GAD+ neurons in 12m (gray, **e**,**f**) and 3m (orange, **g**,**h**) overstimulated and control (black) animals (VGAT size, MWRST, 12m: P=0.005; 3m: P=0.083; VGAT density, Welch's t-test, 12m: P=0.599; MWRST, 3m: P=0.562). Inset shows example of VGAT puncta (red) and GAD+ neuron (blue). Scale bar: 5µm. **i**, Normalized (to baseline) change in average correlation coefficients between inhibitory-with-inhibitory (red) assemblies in 3m (left, filled orange) and 12m (right, filled gray) adult mice at baseline (BL) and following overstimulation (Post-stim) (Wilcoxon signed rank test, Inh-Inh norm correlations, BL vs Post-stim, 3m: P=0.234; 12m: P=0.974). Data from 3 animals for panels a-c and 8 animals for panels d-i. In all panels, *P<0.05, **P<0.01, ***P<0.001, and NS, not significant. Two-sided tests were used throughout, for detailed statistical reporting see Table S11. Data are presented as percentages, cumulative distribution, or as mean ± s.e.m.

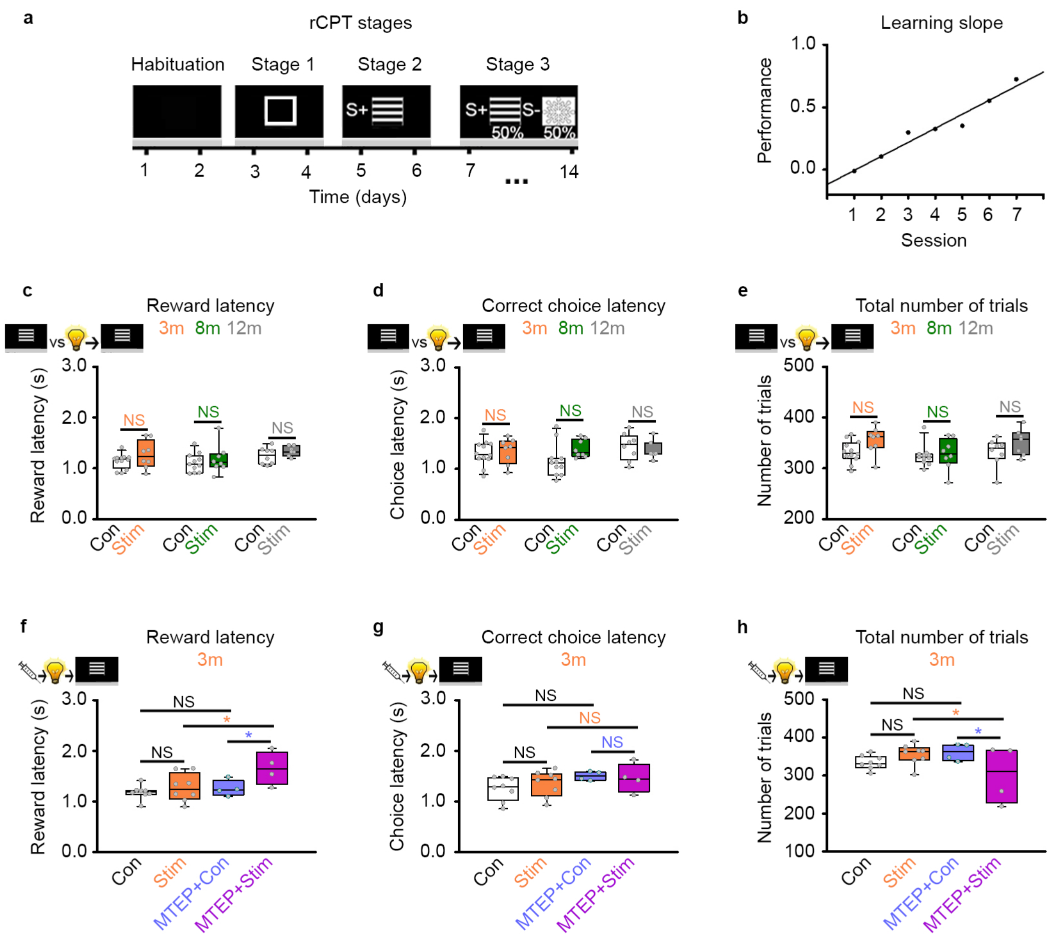

a
rCPT stages
Habituation
Stage 1
Stage 2
Stage 3
S+
S+
S-
50%
50%
Time (days)
b
Learning slope
Performance
Session
c
Reward latency
3m 8m 12m
Reward latency (s)
d
Correct choice latency
3m 8m 12m
Choice latency (s)
e
Total number of trials
3m 8m 12m
Number of trials
Con
Stim
NS
f
Reward latency
3m
Reward latency (s)
g
Correct choice latency
3m
Choice latency (s)
h
Total number of trials
3m
Number of trials
Con
Stim
MTEP+Con
MTEP+Stim
NS
*

Figure S6

Supplementary Fig. 6 | Overstimulation drives an age-related disruption of cognitive performance on the rCPT task.

a, Timeline of the rCPT stages (see Methods). **b,** Representative performance over 7 sessions during stage 3. To estimate animals' overall performance, a linear fit was used to calculate the learning rate across sessions (slope = 0.11 * performance/session, see Methods). **c-e**, Reward latency (**c**), correct choice latency (**d**) and total number of trials (**e**) for overstimulated mice at 3m (orange), 8m (green) and 12m (gray) and age-matched control (white) mice (Two-Way ANOVA, Reward latency, Con vs Stim, 3m: P=0.155; 8m: P=0.436; 12m: P=0.380; Choice latency, Con vs Stim, 3m: P=0.728; 8m: P=0.054; 12m: P=0.601; Trials, Con vs Stim, 3m: P=0.055; 8m: P=0.731; 12m: P=0.152). **f-h**, Reward latency (**f**), correct choice latency (**g**) and total trials (**h**) for mice that received either no stimulation (Con, white), overstimulation (Stim, orange), MTEP and control (MTEP+Con, blue), or MTEP and overstimulation (MTEP+Stim, purple) (Two-Way ANOVA, reward latency, Con vs Stim: P=0.414; MTEP+Con vs MTEP+Stim: P=0.025; Con vs MTEP+Con: P=0.572; Stim vs MTEP+Stim: P=0.014; choice latency, Con vs Stim: P=0.407; MTEP+Con vs MTEP+Stim: P=0.797; Con vs MTEP+Con: P=0.096; Stim vs MTEP+Stim: P=0.461; trials, Con vs Stim: P=0.248; MTEP+Con vs MTEP+Stim: P=0.032; Con vs MTEP+Con: P=0.237; Stim vs MTEP+Stim: P=0.026). Data from 54 animals for panels c-e, and 24 animals for panels f-h. In all panels, *P<0.05, and NS, not significant. Two-sided tests were used throughout, for detailed statistical reporting see Table S12. Data are presented as median and IQR (box plots, box = quartiles, whiskers = 10th and 90th percentiles) with individual data points (gray dots).

Table S13. List of reagents.

Reagent	Source	Identifier	Concentration
AF405 (goat anti-rabbit)	Abcam	ab175654	1:500
AF488 (goat anti-rabbit)	Life Technologies	A11034	1:500
AF568 (goat anti-guinea-pig)	Life Technologies	A11075	1:500
AF647 (goat anti-chicken)	Life Technologies	A21449	1:500
AF647 (goat anti-mouse)	Life Technologies	A11002	1:500
AF647 (goat anti-rat)	Life Technologies	A21247	1:500
c-Fos (rabbit polyclonal)	Abcam	ab190289	1:1000
DAPI	Life Technologies	D1306	300nM
eEF2k (rabbit polyclonal)	Thermo Fisher	PA5-22175	1:500
GAD65/67 (rabbit polyclonal)	Sigma	G5163	1:2000
GluA2 (guinea-pig polyclonal)	Synaptic Systems	182105	1:500
GRIP1 (mouse monoclonal, clone #H-4)	Santa Cruz	sc-365937	1:500
Homer1 (chicken polyclonal)	Synaptic Systems	160006	1:500
Parvalbumin (mouse monoclonal; clone #235)	Swant	PV235	1:2000
Somatostatin (rat monoclonal, clone #M09204)	Abcam	ab30788	1:200
VGAT (mouse monoclonal; clone #117G4)	Synaptic Systems	131 011	1:1000
