# Supplementary material for: Age-related dysregulation of homeostatic control in neuronal microcircuits
Source: Nat Neurosci. 2023 Nov 2;26(12):2158–70. doi: 10.1038/s41593-023-01451-z (PMC10689243; doi:10.1038/s41593-023-01451-z)
Supplement: Supplementary file 2 — Reporting Summary [file 41593_2023_1451_MOESM2_ESM.pdf]

nature portfolio

Corresponding author(s): Samuel J Barnes

Last updated by author(s): 06/07/2023

Reporting Summary

Nature Portfolio wishes to improve the reproducibility of the work that we publish. This form provides structure for consistency and transparency in reporting. For further information on Nature Portfolio policies, see our Editorial Policies and the Editorial Policy Checklist.

Statistics

For all statistical analyses, confirm that the following items are present in the figure legend, table legend, main text, or Methods section.

n/a	Confirmed	
☐	☒	The exact sample size (*n*) for each experimental group/condition, given as a discrete number and unit of measurement
☐	☒	A statement on whether measurements were taken from distinct samples or whether the same sample was measured repeatedly
☐	☒	The statistical test(s) used AND whether they are one- or two-sided<br>*Only common tests should be described solely by name; describe more complex techniques in the Methods section.*
☐	☒	A description of all covariates tested
☐	☒	A description of any assumptions or corrections, such as tests of normality and adjustment for multiple comparisons
☐	☒	A full description of the statistical parameters including central tendency (e.g. means) or other basic estimates (e.g. regression coefficient) AND variation (e.g. standard deviation) or associated estimates of uncertainty (e.g. confidence intervals)
☐	☒	For null hypothesis testing, the test statistic (e.g. *F*, *t*, *r*) with confidence intervals, effect sizes, degrees of freedom and *P* value noted<br>*Give P values as exact values whenever suitable.*
☒	☐	For Bayesian analysis, information on the choice of priors and Markov chain Monte Carlo settings
☐	☒	For hierarchical and complex designs, identification of the appropriate level for tests and full reporting of outcomes
☐	☒	Estimates of effect sizes (e.g. Cohen's *d*, Pearson's *r*), indicating how they were calculated

Our web collection on statistics for biologists contains articles on many of the points above.

Software and code

Policy information about availability of computer code

Data collection	All in vivo imaging and electrophysiology data were collected using either Matlab R2019b (MathWorks), Multiclamp (700B, Molecular Devices) and/or Wavesurfer (v2.1.0 Janelia). All confocal imaging data were collected using Leica Application Suite X (LAS X, v3.5.7). All behavioral experiments data were collected using Campden Instruments ABET Cognition Software.
Data analysis	Analysis was performed using: Matlab R2019b (MathWorks), SigmaPlot v.14 (Systat Software Inc.), ImageJ v1.53t (Fiji), or the MiniAnalysis programme (Synaptosoft, v6.0.7).

For manuscripts utilizing custom algorithms or software that are central to the research but not yet described in published literature, software must be made available to editors and reviewers. We strongly encourage code deposition in a community repository (e.g. GitHub). See the Nature Portfolio guidelines for submitting code & software for further information.

Data

Policy information about availability of data

All manuscripts must include a data availability statement. This statement should provide the following information, where applicable:

- Accession codes, unique identifiers, or web links for publicly available datasets
- A description of any restrictions on data availability
- For clinical datasets or third party data, please ensure that the statement adheres to our policy

Raw data used in this study and requests for resources and reagents are available from the corresponding author upon reasonable request. All raw data necessary

to reproduce all figures are available within the Source data provided with this paper.

Research involving human participants, their data, or biological material

Policy information about studies with human participants or human data. See also policy information about sex, gender (identity/presentation), and sexual orientation and race, ethnicity and racism.

Reporting on sex and gender	No human data was used in this study.
Reporting on race, ethnicity, or other socially relevant groupings	No human data was used in this study.
Population characteristics	No human data was used in this study.
Recruitment	No human data was used in this study.
Ethics oversight	No human data was used in this study.

Note that full information on the approval of the study protocol must also be provided in the manuscript.

Field-specific reporting

Please select the one below that is the best fit for your research. If you are not sure, read the appropriate sections before making your selection.

☒ Life sciences ☐ Behavioural & social sciences ☐ Ecological, evolutionary & environmental sciences

For a reference copy of the document with all sections, see nature.com/documents/nr-reporting-summary-flat.pdf

Life sciences study design

All studies must disclose on these points even when the disclosure is negative.

Sample size	Sample size was based on pilot data used to estimate the impact of sensory overstimulation on c-Fos activity.
Data exclusions	Electrophysiological data were excluded if they did not meet standard inclusion criteria relating to recording parameters such as access resistance. In vivo cellular imaging data were excluded if motion artifacts could not be corrected or if cells exhibited filled nuclei due to GCaMP overexpression.
Replication	Findings were replicated using both technical and biological replicates. Furthermore major biological results were reproduced using additional techniques, such as changes in synaptic strength being measured both ex vivo with electrophysiology and in vivo with synaptic calcium imaging. Biological and technical replicates are reported in the figure legends and the statistics tables.
Randomization	For all experiments animals were randomly assigned to control, overstimulation or intervention groups.
Blinding	All experiments and analysis were conducted blind to the condition and/or temporal order of the acquired data.

Reporting for specific materials, systems and methods

We require information from authors about some types of materials, experimental systems and methods used in many studies. Here, indicate whether each material, system or method listed is relevant to your study. If you are not sure if a list item applies to your research, read the appropriate section before selecting a response.

Materials & experimental systems

n/a	Involved in the study	
☐	☒	Antibodies
☒	☐	Eukaryotic cell lines
☒	☐	Palaeontology and archaeology
☐	☒	Animals and other organisms
☒	☐	Clinical data
☒	☐	Dual use research of concern
☒	☐	Plants

Methods

n/a	Involved in the study	
☒	☐	ChIP-seq
☒	☐	Flow cytometry
☒	☐	MRI-based neuroimaging

Antibodies

Antibodies used	Primary antibodies used: anti-c-Fos (rabbit polyclonal, [1:1000], Abcam cat #ab190289); anti-VGAT (mouse monoclonal, clone #117G4, [1:1000], Synaptic Systems, cat #131 011); anti-GAD65/67 (rabbit polyclonal, [1:2000], Sigma, cat #G5163); anti-Parvalbumin (mouse monoclonal, clone #235, [1:2000], Swant, cat #PV235); anti-Somatostatin (rat monoclonal, clone #M09204, [1:200], Abcam, cat #ab30788), anti-Homer1 (chicken polyclonal, [1:500], Synaptic Systems, cat #160006), anti-GluA2 (guinea-pig polyclonal, [1:500], Synaptic Systems, cat #182105), anti-GRIP1 (mouse monoclonal, clone #H-4, [1:500], Santa Cruz, cat #sc-365937), anti-eEF2k (rabbit polyclonal, [1:500], Thermo Fisher, cat #PA5-22175). Secondary antibodies were used: AF405 (goat anti-rabbit, [1:500], Abcam, ab175654-500ug), AF488 (goat anti-rabbit [1:500], Life Technologies, cat #A11034), AF568 (goat anti-guinea-pig, [1:500], Life Technologies, #A11075), AF647 (goat anti-mouse, [1:500], Life Technologies #A11002), AF647 (goat anti-rat, [1:500], Thermo Fisher #A21247) and AF647 (goat anti-chicken, [1:500], Life Technologies, #A21449).
Validation	Quality control information and relevant citations are available at manufacturer's website. For anti-c-Fos: https://www.abcam.com/c-fos-antibody-bsa-free-ab190289.html; for anti-VGAT: https://sysy.com/product/131011; for anti-GAD65/67: https://www.sigmaaldrich.com/GB/en/product/sigma/g5163; for anti-Parvalbumin: https://www.labome.com/product/SWant/PV235.html; for anti-Somatostatin: https://www.abcam.com/somatostatin-antibody-m09204-ab30788.html; for anti-Homer1: https://sysy.com/product/160006; for anti-GluA2: https://sysy.com/product/182105; for anti-GRIP1: https://www.scbt.com/p/grip1-antibody-h-4; for anti-eEF2k: https://www.thermofisher.com/antibody/product/EEF2K-Antibody-Polyclonal/PA5-22175.

Animals and other research organisms

Policy information about studies involving animals; ARRIVE guidelines recommended for reporting animal research, and Sex and Gender in Research

Laboratory animals	We used adult (P60-360) male and female mice (C57BL/6J) for all experiments. The exception was a subset of experiments where we used young (P60-P100) Thy1-eGFP on a C57BL/6J background (JAX stock #007788, The Jackson Laboratory, Bar Harbor, ME). Mice were housed (temp. 21±2°C; humidity, 55±10%) with littermates on a 12h light-dark cycle with access to water and food ad libitum.
Wild animals	We did not use wild animals.
Reporting on sex	All mice were sex and age matched between experimental groups.
Field-collected samples	The study did not involve samples from the field.
Ethics oversight	Experiments were conducted according to the United Kingdom Animals (Scientific Procedures) Act 1986.

Note that full information on the approval of the study protocol must also be provided in the manuscript.
